# BacArena: Individual-based metabolic modeling of heterogeneous microbes in complex communities

**DOI:** 10.1371/journal.pcbi.1005544

**Published:** 2017-05-22

**Authors:** Eugen Bauer, Johannes Zimmermann, Federico Baldini, Ines Thiele, Christoph Kaleta

**Affiliations:** 1 Luxembourg Centre for Systems Biomedicine, Université du Luxembourg, Esch-sur-Alzette, Luxembourg; 2 Research Group Medical Systems Biology, Institute for Experimental Medicine, Christian-Albrechts-University Kiel, Kiel, Germany; The Pennsylvania State University, UNITED STATES

## Abstract

Recent advances focusing on the metabolic interactions within and between cellular populations have emphasized the importance of microbial communities for human health. Constraint-based modeling, with flux balance analysis in particular, has been established as a key approach for studying microbial metabolism, whereas individual-based modeling has been commonly used to study complex dynamics between interacting organisms. In this study, we combine both techniques into the R package BacArena (https://cran.r-project.org/package=BacArena) to generate novel biological insights into *Pseudomonas aeruginosa* biofilm formation as well as a seven species model community of the human gut. For our *P. aeruginosa* model, we found that cross-feeding of fermentation products cause a spatial differentiation of emerging metabolic phenotypes in the biofilm over time. In the human gut model community, we found that spatial gradients of mucus glycans are important for niche formations which shape the overall community structure. Additionally, we could provide novel hypothesis concerning the metabolic interactions between the microbes. These results demonstrate the importance of spatial and temporal multi-scale modeling approaches such as BacArena.

## Author summary

In nature, organisms are typically found in near proximity to each other, forming symbiotic relationships. Particularly bacteria are often part of highly organized communities such as biofilms. In this study, we integrate the detailed knowledge about the metabolic capabilities of individual organisms into an individual-based modeling approach for simulating the dynamics of local interactions. We provide a fast and flexible framework, in which established computational models for individual organisms can be simulated in communities. Nutrients can diffuse in an area where cells move, divide, and die. The resulting spatial as well as temporal dynamics and metabolic interactions can be analyzed as well as visualized and subsequently compared to experimental findings. We demonstrate how our approach can be used to gain novel insights on dynamics in single species biofilm formation and multi-species intestinal microbial communities.

## Introduction

A major goal in microbial systems biology is to understand metabolic mechanisms underlying the emergence and organization of microbial communities [1]. Metabolic processes have been suggested to modulate and organize complex community structures by cross-feeding interactions (exchange of nutrients) [2, 3]. The human gut microbiota, for instance, consists of hundreds of species [4], whose compositions is strongly influenced by metabolic factors such as diet and microbial physiology [5]. Especially the metabolic interactions of multi-species communities within the gut have been found to support human well-being by the supplementation of nutrients via fermentation of otherwise indigestible dietary components [6]. One of the most important hallmarks in determining a healthy gut structure is the integrity of the mucus layer, which covers the epithelium, acts as a protective barrier against intruding pathogens, and enriches beneficial bacteria by providing nutritional compounds such as glycans [7]. Therefore, concentration gradients of substrates induce a spatial differentiation of the microbial community.

In biofilms, spatial concentration gradients of metabolites lead to a differential nutrient availability and therefore govern the distribution of species and phenotypes [2, 3]. Therefore, considering the processes that generate chemical gradients is essential when studying physiological heterogeneity in biofilms [3, 8, 9]. Individuals of the same or different species can support each other’s growth by metabolic cross-feeding interactions [10]. Conversely, competition for nutrients can induce a division of metabolic tasks within the community which spatially differentiates the population in different sections, e.g. metabolically active and inactive microbe cells [11]. Such self-organizing processes have important implications in biomedical applications since single-species biofilms of pathogens are associated with a higher resistance against antibiotics [12] and thus obstructing potential treatments for diseases. In particular, most antibiotics are targeted at growing bacteria and not metabolically inactive dormant cell, which could re-initiate the biofilm after antibiotic treatment [11]. Furthermore, due to the physical structure of biofilms, antibiotics could poorly penetrate and often remain ineffective [13].

Constraint-based reconstruction and analysis (COBRA) is a key approach for the *in silico* study of microbial metablism [14]. Metabolic reconstructions comprise the complete set of biochemical reactions derived from a genome annotation in a stoichiometric accurate manner [15]. Through the application of specific constraints (e.g. nutrient availability) they can be converted into condition-specific models. With flux balance analysis (FBA), these models are used to optimize a given objective, such as the growth yield under a metabolic steady state [16]. To model metabolic interactions within microbial communities, different COBRA-based approaches have been developed [17]. First approaches modeled bacterial communities by combining the reconstructions of single microbes into a metabolic model, where metabolites can be exchanged and community growth is maximized using FBA [18, 19]. This concept has been recently expanded to allow integration of experimental data and modeling of distributed community growth [20]. Additional approaches have included temporal dynamics, in which microbial growth is simulated [21–23]. Recent advances incorporated spatial dynamics by enabling the distribution of microbes and metabolites, assuming homogeneous species populations [24]. Spatial environments were also used in an approach called MatNet [25] which combines FBA with individual-based modeling to simulate the metabolism of single species biofilms. Unlike population modeling, individual-based approaches allow to analyze populations as aggregations of autonomous individuals that interact by a set of rules. Accordingly, complex dynamics arise as emergent properties of locally interacting individuals [26–28].

In this study, we develop and apply BacArena, a community modeling tool which extends the integration of FBA and individual based modeling proposed by MatNet to model multi-species communities. Essentially, we model populations as aggregations of heterogeneous individuals that have their own metabolism and interact spatially as well as temporarily according to biologically relevant rules (e.g., movement, chemotaxis, and lysis). Furthermore, by modeling such metabolic heterogeneity, we can generate novel hypothesis concerning cross-feeding interactions within and between species. In particular, we applied BacArena to model *Pseudomonas aeruginosa* biofilm formation on the level of metabolic phenotypes. We could show how individuals are spatially arranged with different phenotypes according to nutrient availability. Furthermore, we identified phenotypes whose fermentation products contributed to growth of other biofilm members. In an application of a simplified human gut consortium consisting of seven species, we found that spatial gradients of mucus glycans are important to shape the community structure by forming a niche for glycan degrading bacteria. Additionally, short chain fatty acids were exchanged between the community members and contributed to concentration levels which were similar to published experimental values. These results underline the increasing relevance of multi-scale modeling tools such as BacArena.

## Results and discussion

**Fig 1:**
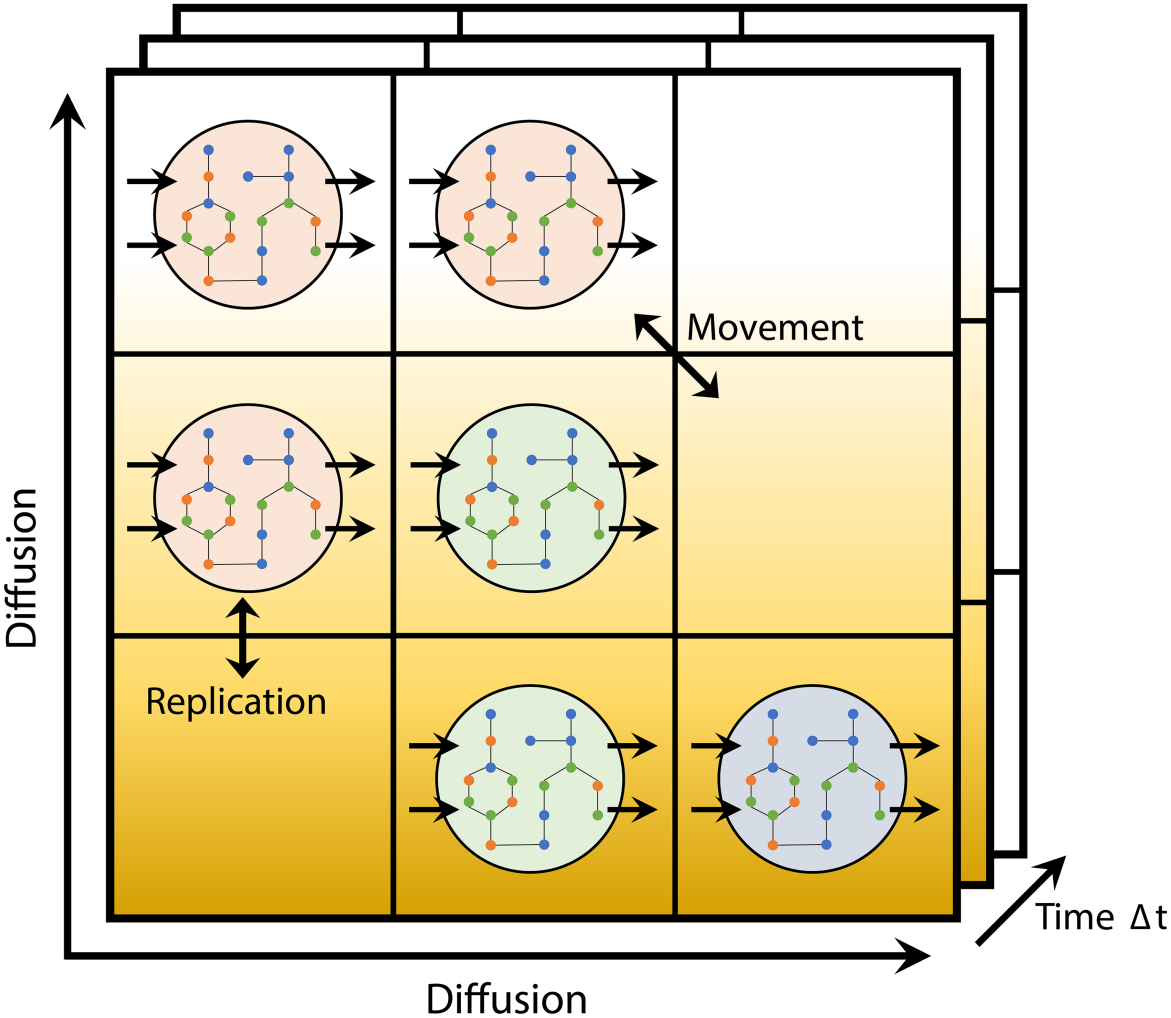
Schematic overview of BacArena. Microbial species are shown in different colors. Fluxes of exchange reactions are indicated as uni-directional arrows, movement and replication as bi-directional arrows.

With BacArena we provide a modular and extendable R package for modeling and analyzing microbial communities (S1 and S2 Text). In BacArena each organism is represented individually on a two-dimensional grid to model a spatial environment (Fig 1). Temporal dynamics are modeled by including time steps in which the state of each individual and the environment is updated. In each time step metabolites diffuse in the environment and can be exchanged between the individuals. Individuals can move to and duplicate within the neighboring grid positions. The metabolism of each individual is modeled by flux balance analysis on the underlying genome-scale metabolic model of the particular species. Using the biomass as an objective for the FBA and the metabolite concentrations in the corresponding grid position as constraints, the growth and metabolic turn over is determined. Accordingly, the duplication rate is obtained from the growth rate and the metabolite concentration is updated according to the secreted and consumed metabolites. Since a FBA is computed for each individual, every microbial cell can be heterogeneous in its metabolism and has therefore its own metabolic profile. These profiles are recorded as metabolic phenotypes in BacArena and can be used to infer cross-feeding interactions.

### Comparison to other methods

Established methods in community modeling can be roughly divided into two groups: Equation based, continuous methods modeling populations (e.g. COMETS, dOptCom) and rule-based methods focusing on individuals (MatNet, BacArena) (Table 1).

**Table 1:** Comparison of BacArena with other community modeling approaches involving metabolic models.

Method	Approach	Time	Kinetics	Space	Phenotypes	Parallel	GUI	Species
BacArena	FBA/ABM	✔	✔	✔	✔	✔		> 2
MatNet [25]	FBA/ABM	✔		✔			✔	1
COMETS [24]	dFBA	✔	✔	✔		✔	✔	> 2
dOptCom [23]	Multi-objective	✔	✔					> 2
MCM [22]	dFBA	✔	✔				✔	> 2
DyMMM [21]	dFBA	✔	✔					2

**Fig 2:**
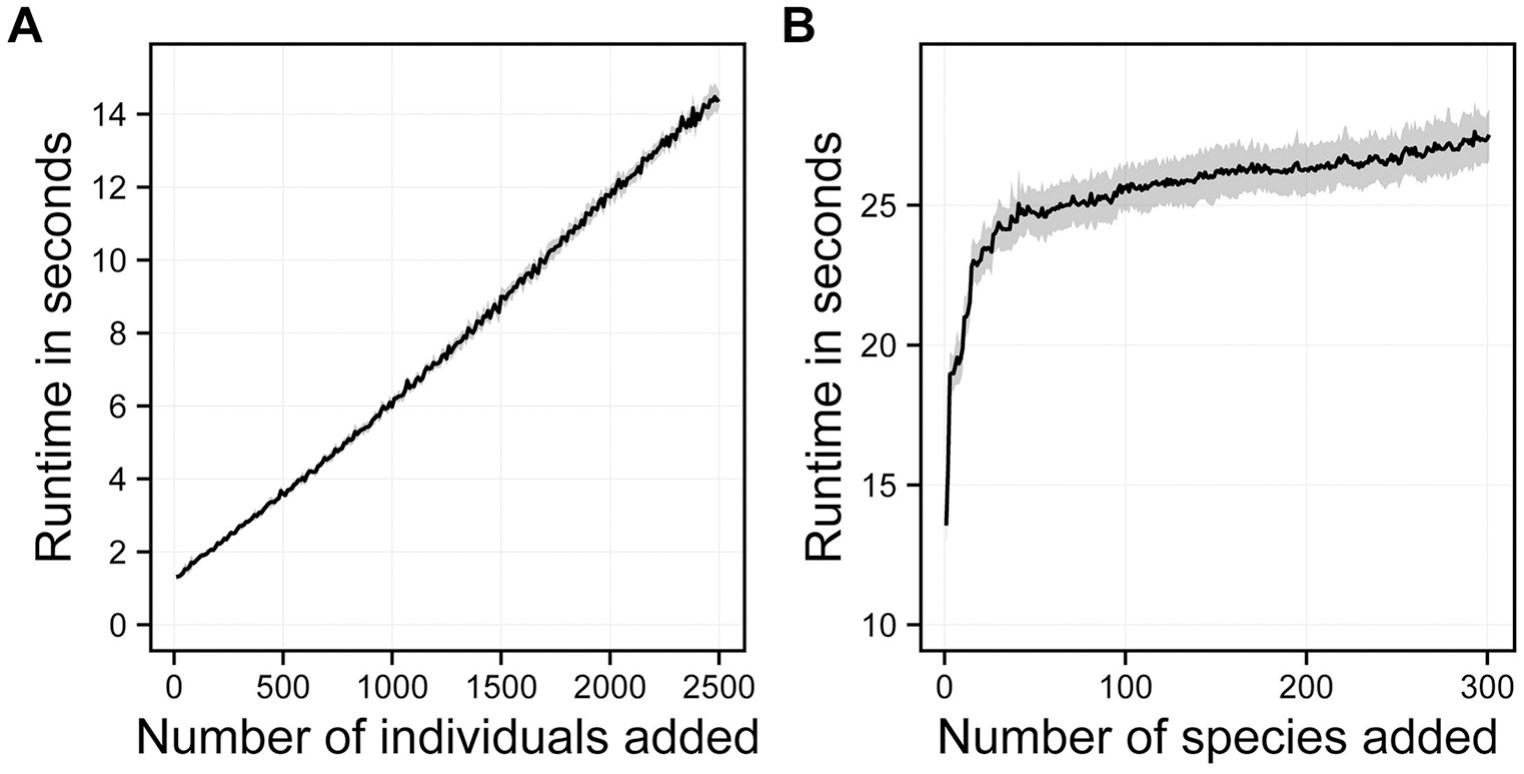
Runtime of BacArena in relation to the number of added individuals and species. **A** Runtime based on an example draft metabolic model (*Clostridium* sp. SY8519 model taken from [30]) with an increasing number of individuals added to an environment with a dimension of 50 times 50 grid cells. **B** Runtime based on an increasing number of species (301 draft metabolic models taken from [30]) added to an environment with a dimension of 50 times 50 grid cells and one simulation step. All simulations were run on a windows machine with 32GB of RAM and a 3.5GHz processor with four physical cores.

BacArena extends the individual-based modeling approach of MatNet [25] to include more features (Table 1) and simulation of up to hundreds species (Fig 2). The runtime of BacArena simulations is linearly dependent on the number of individuals (Fig 2A) and increases till an addition of about 50 species (Fig 2B). Afterwards the runtime remains approximately stable because the diffusion of metabolites is computationally expensive and if including more than 50 species only few new metabolites need to be added. BacArena was developed to run efficiently even with large data sets due to R’s capacity to integrate C++ code into time-consuming routines [29]. Additionally, computations can be executed in parallel to accelerate runtime.

**Fig 3:**
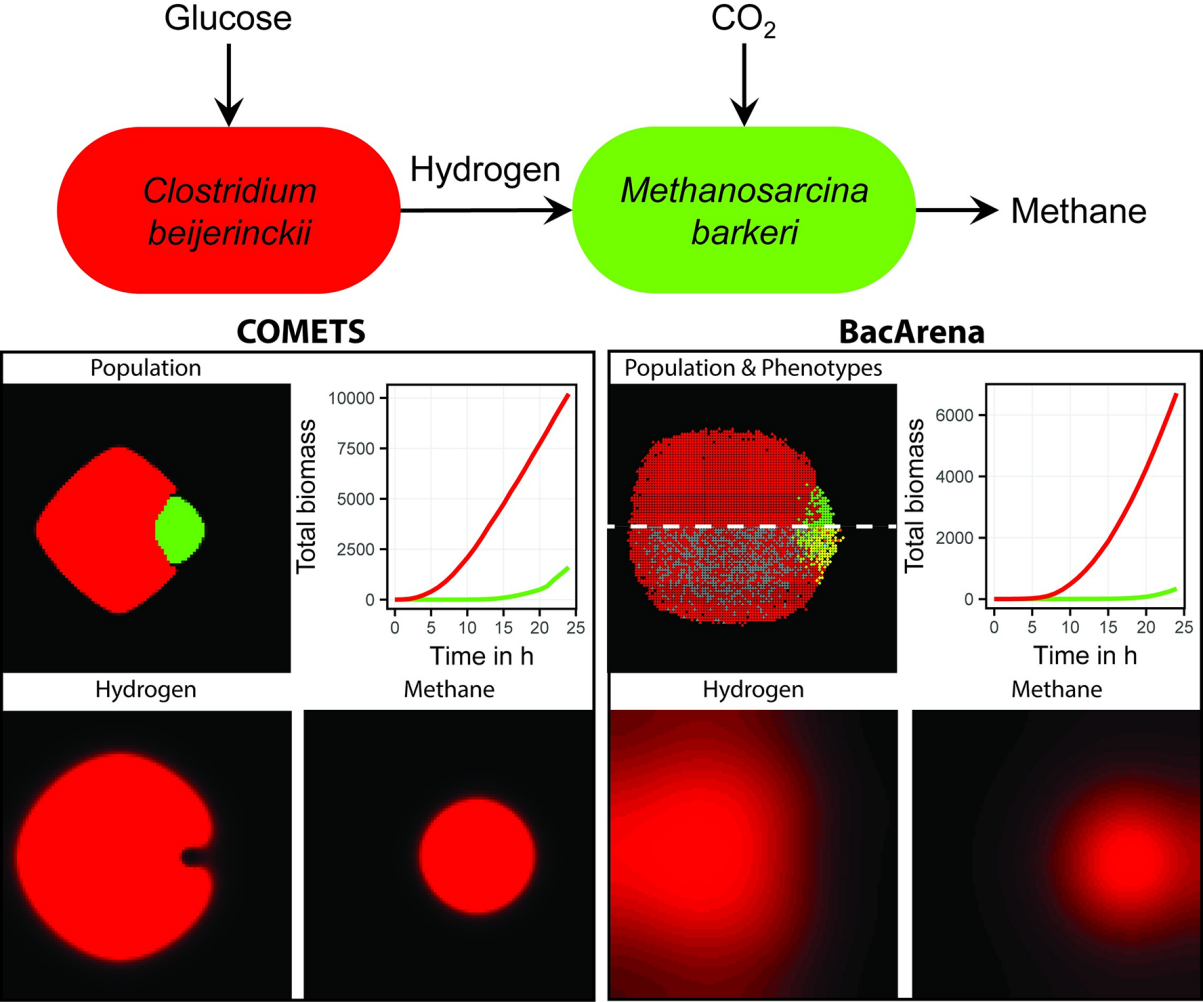
Comparison between COMETS and BacArena based on a simple two-species syntrophic community. The community is based on the published metabolic model for the hydrogen producing *Clostridium beijerinckii* [35] and the methanogen *Methanosarcina barkeri* [36]. Both simulations were carried out on a 100 times 100 grid environment. As initial concentrations, 1 mmol of glucose, carbon dioxide, and several co-factors were added per grid position. Grey cells in the phenotype plot of BacArena (lower half of the population plot) represent metabolically inactive cells.

To illustrate the difference between continuous and rule-based population modeling approaches, we compared BacArena and COMETS [24] in the context of a two-species syntrophic community of the methanogenic archeum *Methanosarcina barkeri* and the hydrogen producing bacterium *Clostridium beijerinckii* (Fig 3). The hydrogen produced by *C. beijerinckii* is taken up as an electron donor by *M. barkeri* to reduce carbon dioxide to methane, which is secreted into the environment. This is in concordance with experimental knowledge, showing the metabolic exchange between hydrogen producing bacteria and methanogenic archaea [31]. Notably, COMETS and BacArena produce similar results in terms of these predicted cross-feeding interactions and are therefore consistent. Based on the quantitative biomass production, both methods predict a smaller growth of *M.barkeri* compared to *C.beijerinckii*, however, the biomass production is higher in COMETS compared to BacArena. For the exponential phase of each simulation COMETS predicted a doubling time of 0.5h, BacArena predicted 1.1h, and the experimentally measured value is 4.3h [32]. The reason for this difference can be attributed to the underlying growth model of both methods. COMETS models colony growth as a 2D diffusion while BacArena models individual cell behavior and replication which causes the population to grow slower in the initial phase to reach a certain number of individuals. In BacArena populations consist of heterogeneous individuals (bottom-up) which have their own characteristics, e.g. movement and metabolic phenotypes. COMETS, on the other hand, is a top-down approach describing colonies on the population level (Fig 3). Both approaches differ concerning the representation of the spatial scale. In BacArena one individual is represented per grid position, whereas COMETS represents a population of multiple cells per position. Both, BacArena and COMETS, can predict heterogeneous growth rates according to spatial concentration gradients. By focusing on individuals, BacArena can be used to model additional heterogeneity of cells by accounting for their history and by integration of further rules such as cellular lysis. The explicit consideration of heterogeneous individuals has been regarded as especially helpful for addressing the complexity of biological systems, because local species interactions can represent biological systems more realistically [28, 33, 34]. In particular, the heterogenic movement in BacArena can be relevant when modeling an aqueous or viscose environment, such as the human gut, in which the movement is accelerated. Furthermore, by combining individual-based modeling with FBA, BacArena can model the metabolic state of each individual cell to investigate metabolic heterogeneity within a population of cells. This metabolic heterogeneity is captured by our definition of metabolic phenotypes, whose applicability and biological relevance we show in the next section on the basis of a biofilm model of *Pseudomonas aeruginosa*.

### *P. aeruginosa* single-species biofilm model

**Fig 4:**
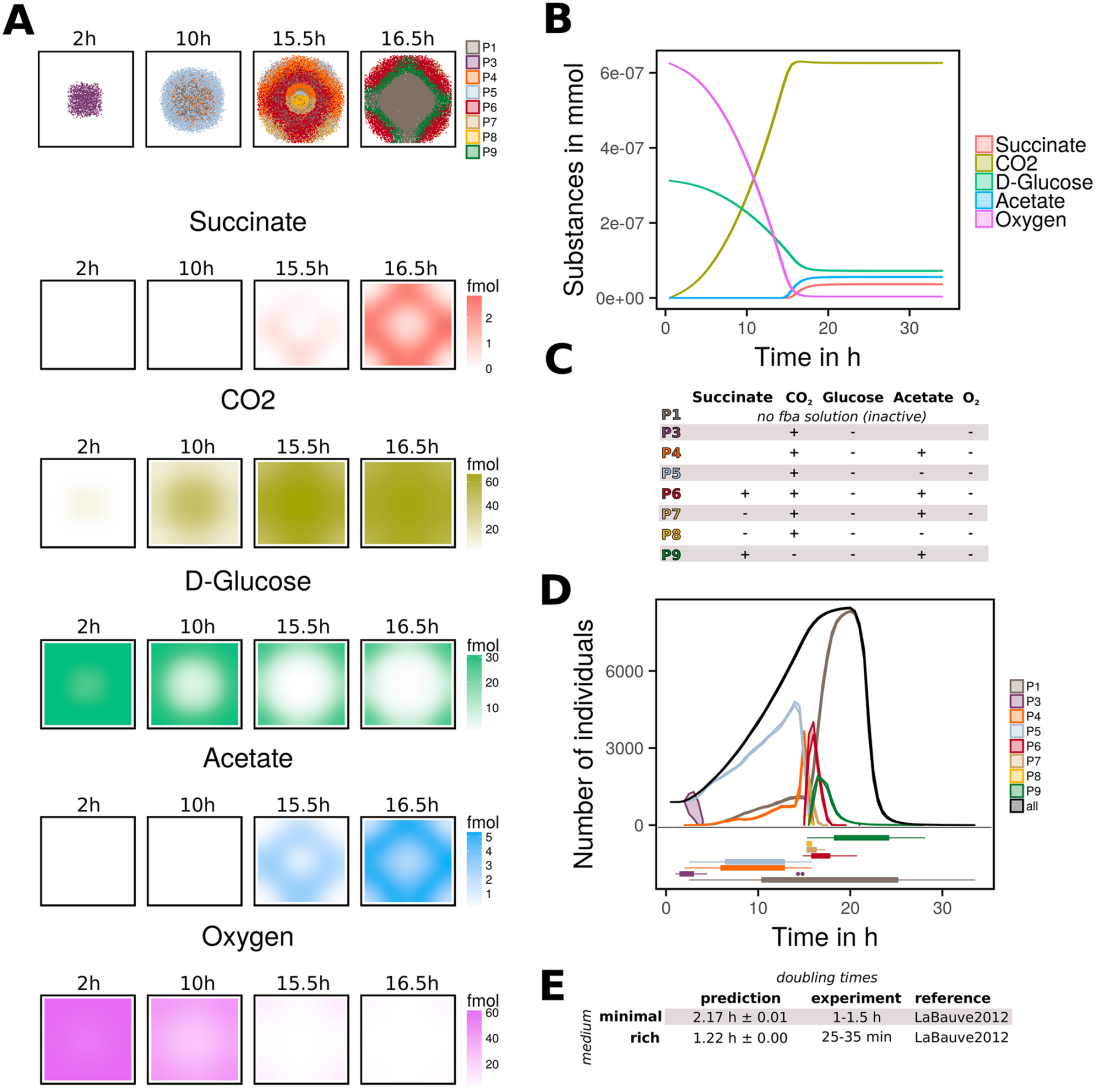
Single species biofilm model of *P. aeruginosa*. **A** Spatial distribution of individuals and key metabolites at different time points (2 h, 10 h, 15.5 h, 16.5 h). Different metabolic phenotypes are colored and represent community members with distinct production and consumption of metabolites. A metabolic inactive core was formed after 16 h and several fermentative phenotypes occurred in the outer layer of the biofilm (see subfigure C for description of phenotypes). Glucose and oxygen were consumed and CO_2_, acetate, succinate were produced. **B** Time curve of key Metabolites. Metabolites are given in mmol. Oxygen was consumed in total and some glucose remained in the end. Acetate and succinate levels increased after 15h. **C** Characterization of eight metabolic phenotypes (P1,P3-P9). Only phenotypes which occurred consistently in all replicates were considered. Therefore, P2 (growth with CO_2_ and acetate) and P10 (acetate and succinate production, glucose and oxygen consumption without CO_2_ release) were not considered. In the table, a plus sign ‘+’ indicates production and a minus sign ‘-’ indicates consumption of metabolites. **D** The growth curve of *P. aeruginosa* colored in black. Additionally, for all phenotypes (P1, P3-P9) the growth curve is shown. To distinguish the different times when a certain phenotype did occur, an integrated boxplot is given below. **E** Comparison of predicted doubling times with experimental findings. Minimal medium and rich medium doubling times were shown.

To demonstrate the applicability of BacArena to biofilm formation, we constructed a single species biofilm model of *P. aeruginosa*. We used a glucose-minimal medium with oxygen as electron acceptor to investigate the metabolic behavior of individual cells of the biofilm community. We found spatial and temporal differences within the community which could be attributed to distinct emergent metabolic phenotypes. The observed phenotypes (P1-P9) were classified according to the usage or production of glucose, oxygen, acetate, succinate, and *CO*_2_ (Fig 4C). The phenotypes occurred in all replicate simulations (n = 10) with similar temporal dynamics (S2 Fig). Additionally, the phenotype appearance was stable with respect to variations in initial glucose and oxygen levels (S3 Text). Finally, we validated the growth model with experimental data [37] and correctly predicted a higher population size under rich conditions compared to a minimal medium (Fig 4E).

In the beginning of the simulation, we observed only a glucose oxidation phenotype (P3) that constituted the whole population (Fig 4A). After two hours the individuals got more metabolically diverse and a division of metabolic tasks and cooperation between phenotypes occurred. Coupled with decreased oxygen levels in the center, a fermenting phenotype (P4) appeared and the produced acetate was consumed by phenotype P5, which appeared subsequently (Fig 4D). The core of the mature biofilm consisted mainly of acetate producers (P4) and metabolically inactive cells that had zero flux through the biomass reaction (P1). The next phase of biofilm formation was characterized by a highly dynamic cooperation and competition between phenotypes. Succinate was released, in addition to acetate, by a new phenotype P6. Fermenting phenotypes, P4 and P6, were most abundant and therefore quantities of acetate and succinate began to rise (Fig 4A and 4D). The newly available succinate was used again by the emerging phenotypes P7 and P8. The production and consumption of different amounts of acetate and succinate under varying oxygen conditions are due to difference in nutrient availability, as shown by independent FBA simulations (see S3 Text). Experimentally it has been shown that *P. aeruginosa* cultures are able to produce acetate, and succinate as fermentation products which also contribute to biofilm survival [38]. Additionally, it has been reported that *P. aeruginosa* is able to use succinate as a carbon source and that the addition of acetate or succinate increased the growth rate [39, 40]. In addition to experimental findings, our simulation identifies fermenting (P4, P6, P7) and absorbing phenotypes (P5, P7, P8) whose interactions contributes to community stability. After about 16.5 hours of simulation, only very small concentrations of oxygen remained and the mature biofilm could be divided into three layers: a metabolic inactive core and two fermenting outer layers (Fig 4A and 4B). Both fermenting layers consisted of acetate and succinate producers (P6, P9). First the outer fermenting layer was formed out of phenotype P6 which grew towards the edges in which the glucose concentration was still high. Afterwards the inner fermenting layer with phenotype P9 showed an additional fixation of *CO*_2_ by the anaplerotic pyruvate carboxylase reaction. In this context it is known that *CO*_2_ can exert both a positive or negative effect on growth of *Pseudomonas* [41, 42] and thus carbon fixation could be possible (more detailed discussion in S3 Text). Our simulation further suggests that *CO*_2_ fixation can have a positive effect on late phase biofilm survival.

Finally the inactive core increased in size and dominated the population after 20 hours with cell death and population decrease. We found oxygen to be the limiting factor (Fig 4B and 4D). Concerning anaerobic physiology, it has been reported that *P. aeruginosa* can grow in microaerobic and anoxic environments [43]. Anoxic growth has been shown either with nitrate or nitrite as alternative electron acceptors [44], or via arginine [45] and pyruvate [38] fermentation by which the former allowed only minor growth and the latter supported survival only [43]. We tested the influence of nitrate as alternative electron acceptor in an additional simulation. When the population consisted mostly of metabolic inactive cells after 20 hours, 0.1 *mM* nitrate was added. Shortly afterwards a new nitrate respiring phenotype P11 replaced the former dominant, metabolic inactive phenotype P1. Therefore, the almost dissolving biofilm culture could be reactivated by adding another terminal electron acceptor instead of oxygen (S3 Fig, [46]).

BacArena demonstrates how emergent metabolic phenotypes could contribute to community formation. We were able to make novel predictions on how these different phenotypes could contribute to biofilm integrity within a spatio-temporal context. Recently a role of metabolic co-dependence between interior and peripheral cells for community stability, resilience, and antibiotic resistance has been described for *B. subtilis* biofilms [11]. Our simulation shows that a similar metabolic cooperation could be possible in *P. aeruginosa* biofilms between micro-aerobically fermenting and aerobic phenotypes. Novel treatments could try to first eliminate the protective outer layer and then target the metabolic cross-feeding of the inner layer to disrupt the overall biofilm structure, by targeting specific metabolic pathways particular to the corresponding phenotypes.

### Integrated multi-species model of a human gut community

**Fig 5:**
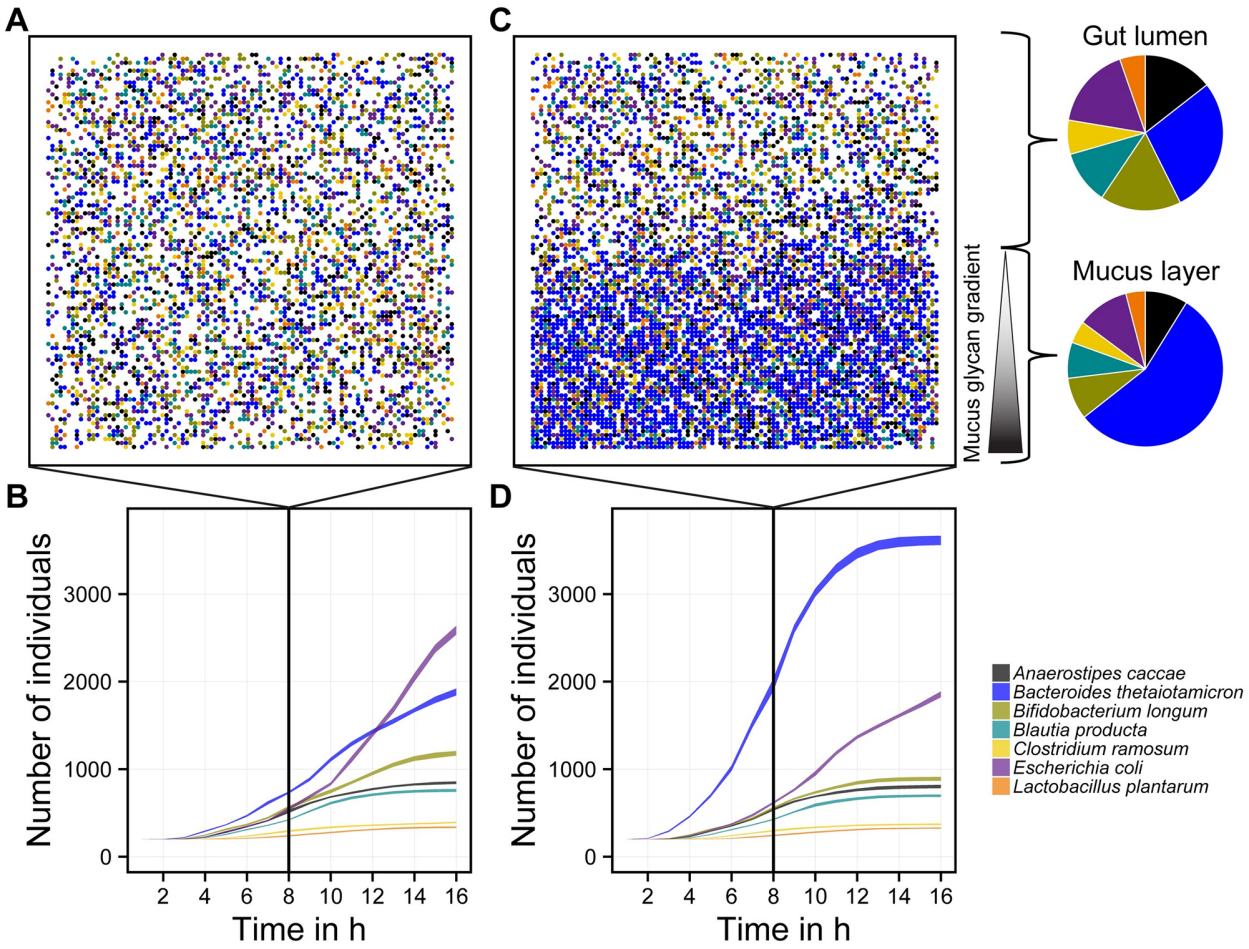
Multi-species community of a minimal human intestinal microbiota (SIHUMI) in rich medium. **A** Spatial population structure in the exponential phase after simulating 8 hours under a uniformly distributed rich medium (all possible metabolites that can be taken up are added to the environment), with **B** the growth curves of each species. **C** Spatial population structure in the exponential phase after 8 hours simulation time under a uniformly distributed rich medium with a spatial gradient of mucus glycans, with **D** the growth curves of each species. The curve range shows the standard deviation of 10 replicate simulations.

We used BacArena to model the multi-species community of the human gut (Fig 5). Since the human gut microbiota typically comprises 500-1000 species [4], we implemented a simplified human intestinal microbiota (SIHUMI) of seven species that has previously been characterized experimentally [47]. In a first condition (Fig 5A), we added all metabolites which can be consumed by at least one species to the environment except mucus glycans. *E. coli* dominated the community after the population reached a stable state at 16 h (Fig 5B). This condition could correspond to a dysbiotic gut environment with intestinal bacterial overgrowth, in which *E. coli* dominates the human gut flora [48]. Interestingly, by adding a more realistic mucus glycan gradient to our model, we could revert the *E. coli* dominance (Fig 5D) and a spatial differentiation of the community between gut lumen and mucus layer emerged. The mucus layer was mostly dominated by *B. thetaiotaomicron*, which is well known to degrade glycans [49]. This result is in accordance with experimental data, which showed the same niche separation between mucus degrading bacteria close to the gut epithelial layer and other microbes in the lumen [50]. Moreover, this spatial differentiation is indicative for a healthy gut microbiota since mucus degrading bacteria can occupy and defend the space close to the epithelium and consequently out-compete intruding pathogens [51]. An impaired mucus secretion can lead to inflammatory bowel disease, where the epithelial barrier is infiltrated by bacteria [52] which cause an inflammation of the gut wall. In this context, our results support recent evidence suggesting that metabolite secretion by the host may play a more important role in shaping the gut microbiome than the immune system itself [53]. Our model therefore predicts that metabolic gradients are relevant in shaping the gut community structure and ecology. This has some important implications in understanding the mucus barrier and indicates that dietary or metabolic treatments might be more relevant than immunosupressors in case of a disrupted mucosal microbiota.

**Fig 6:**
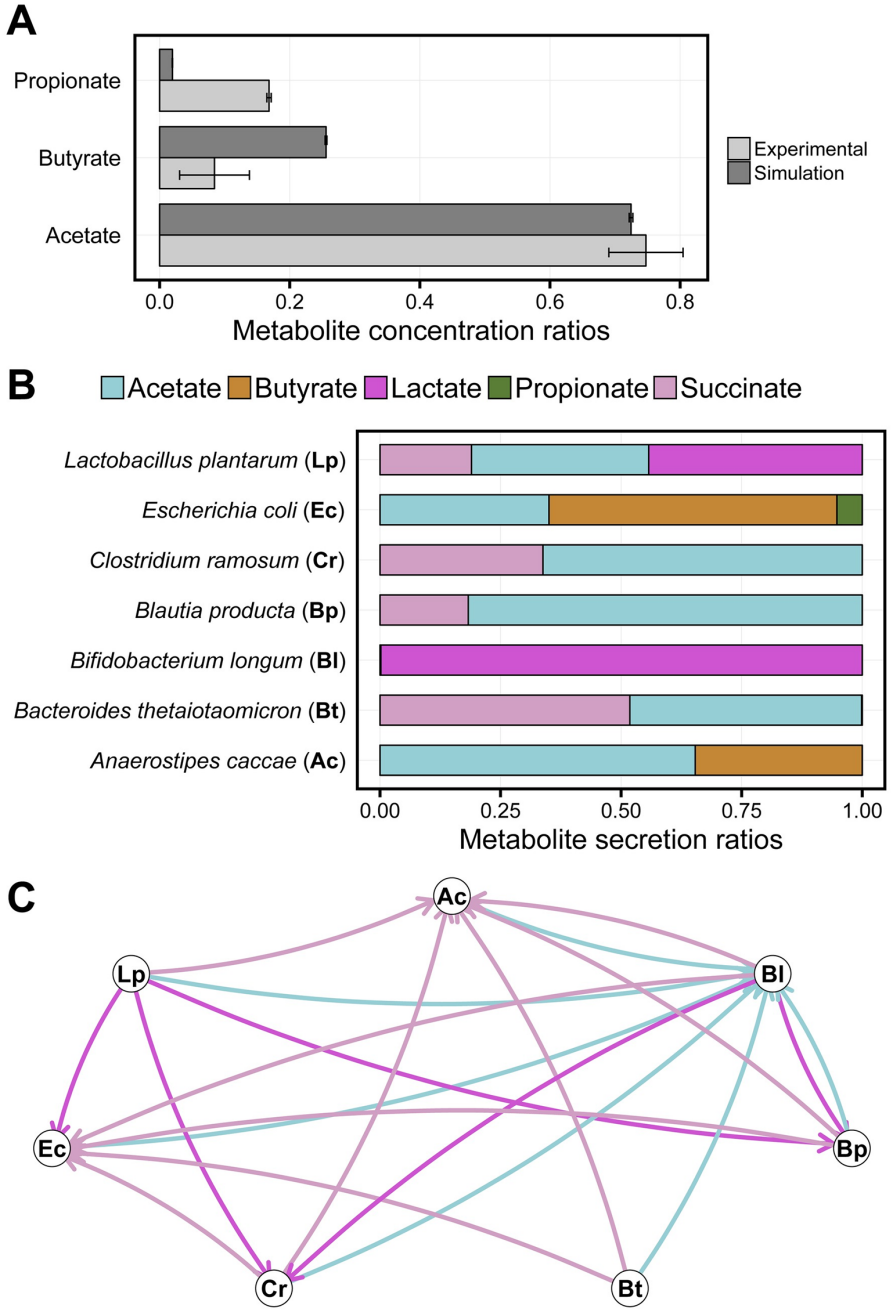
Influence of mucus glycan gradients on community dynamics. **A** Comparison of simulated metabolite concentrations with experimental values based on *in vitro* SIHUMI co-cultures [47]. **B** Metabolite secretion rates of different microbes in our SIHUMI model, determined by the overall metabolic secretion flux of the populations comprising all individuals. **C** Emerging metabolic interaction network of different fermentation products that can be exchanged between the microbe population in our SIHUMI model. Nodes represent species and edges represent exchanged metabolites, which are directed from the secreting species to the consuming species. The secretion and uptake was determined by the overall metabolic flux of the populations comprising all individuals.

Next, we focused on the underlying metabolic mechanisms influencing the overall ecological structure of our setup which includes the mucus glycans (Fig 5C). As expected from human gut studies [54], we found the fermentation products succinate, acetate, lactate, propionate, and butyrate (Fig 6B) to be produced and, in some cases, exchanged between the microbes (Fig 6C). As for propionate, butyrate, and acetate, we could compare our predictions (Fig 6A) to the initial experimental study which describes the SIHUMI microbiota and *in vitro* co-culture experiments [47]. We found that the metabolite concentration ratios are comparable to experimental values with minimal higher butyrate and lower propionate concentrations (Fig 6A). Since BacArena allows to assess the metabolic phenotype of individual cells, we are able to derive hypotheses concerning cross-feeding of fermentation products. In particular, succinate was the metabolite with the most diverse metabolic exchange among the present metabolites (Fig 6C). This observation is in concordance with experimental findings suggesting an importance of succinate cross-feeding between human gut microbes [55]. In addition to succinate, acetate was also a key component to cross-feeding interactions between the microbes of our simplified community (Fig 6C). Acetate was produced by all species except *B. longum* (Fig 6B). This might explain the experimentally observed high levels of acetate concentrations in the human large intestine [56], likely resulting from an over-production of acetate compared to its consumption. Furthermore, the relatively high concentration of acetate is also in concordance with experimental studies on the SIHUMI model microbiota (Fig 6A). As expected, lactate was mainly produced by the lactic acid bacteria *B. longum* and *L. plantarum*, and consumed by *B. producta*, *C. ramosum*, and *E.coli* (Fig 6B). Butyrate was released by *A. caccae* and *E.coli* and was not part of any cross-feeding interactions (Fig 6C). The remaining butyrate could therefore be potentially absorbed by the host epithelium as a main metabolite for energy conversion [10].

To investigate the impact of alternative optimal FBA solutions on the reproducibility of our results, we randomized the selection of alternative optimal solutions and checked the simulations against each other (S4 Fig). We found that growth curves did not change with differing methods, which we expected since our simulated alternative optimal solutions have the same objective value (in our case the growth rate). Despite some metabolite concentrations variations (S4 Fig), the general trend was consistent and thus we concluded our results to be stable.

The predicted metabolite concentrations and cross-feeding interactions of our model (Fig 6C) give novel insights into how the simultaneous exchange of multiple fermentation products is relevant in shaping the human gut microbiota.

### Conclusion

Following the systems biology paradigm, we presented a novel approach to study cellular communities. BacArena enables the analysis of interaction dynamics on the level of individuals and can therefore contribute to current efforts to move from correlative to functional explanations.

In context of a single-species biofilm of *P. aeruginosa*, we could show how a dynamic series of locally interacting metabolic phenotypes contributed to the emergence of an overall biofilm structure. We found that within species metabolic heterogeneity is an important contributor to community dynamics. The spatial differentiation in biofilms has been shown to have important implication in biofilm stability and integrity since the outer layer can act as protective barrier and the inner core can serve as a seed to initiate a new biofilm by supplying metabolites after antibiotic treatment [11, 13].

Additionally, we used BacArena to study the dynamics of gut microbes interacting within the epithelial mucus layer, which has important implications in inflammatory bowel disease [52]. As multi-scale modeling approaches become more relevant in studying the gut microbiome [57], BacArena provides an important contribution since it allows explore the relevance of metabolic interactions in the dynamics of such communities.

## Methods

In principle, any genome-scale metabolic model in SBML or spreadsheet format can be imported and manipulated via sybil [58] and then directly integrated in BacArena. A hands-on tutorial for BacArena is available to illustrate specific use-cases and to get familiar with the code (S1 Text).

### Concept and basic implementation of BacArena

We combine flux balance analysis (FBA) with individual based modeling. Each metabolic model belongs to an independent individual on a two-dimensional *n* × *m* grid environment (Fig 1) and acts according to biologically relevant rules (Table 2).

**Table 2:** List of rules implemented in BacArena and their corresponding references obtained from experimental studies.

Name	Description	Implementation	Ref
Metabolism	Computation of reactions speeds (fluxes)	Flux balance analysis (FBA)	[16]
Metabolism	Computing fluxes while minimizing enzyme usage	Parsimonious FBA	[59]
Kinetics	Defined metabolite uptake	Michaelis-Menten kinetics	[60]
Movement	Movement of individual cells	Random position change	[61]
Chemotaxis	Directed movement towards concentration gradient	Position change according to concentrations	[61]
Lysis	Cellular lysis after death	Secretion of biomass compounds	[62]
Growth	Biomass increase of each organism	Exponential and linear biomass increase	[63]
Death	Death of each organism	Organism death according to biomass threshold	[63]
Diffusion	Distribution of metabolites	Diffusion by partial differential equation	[64]

Consequently, FBA is a complex rule defined for an individual to compute the flux through all *r* biochemical reactions (flux vector $v\in {\mathbb{R}}^{n}$) by optimization of an objective function *c*^*T*^ *v* (e.g., maximization of biomass yield). The corresponding linear programming problem can be written as follows:

$$\begin{array}{cc} \text{Maximize}\; & c^{T}v\\ \text{Subject}\;\text{to}\; & S\cdot v=0\\ & l\leq v\leq u \end{array}$$ (1)

where $S\in {\mathbb{R}}^{m\times n}$ denotes the stoichiometric matrix (*m* number of metabolites in an individual) and the vectors *l* and *u* represent the lower and upper bounds on *n* reactions respectively. The lower bounds of the external metabolite exchange are constrained according to the metabolite concentrations $[C_{i,j}]\in {\mathbb{R}}^{m}$ available at an individual’s position (*i*, *j*) on the grid. All metabolites are initialized according to a initial concentration. Computed fluxes update the concentrations in every time step. Concentrations could be used as flux constraints because they represent the availability of the metabolites in the environment and therefore represent the uptake limit. Alternatively, if kinetic parameters are defined by the user, the lower bounds can be constrained according to Michaelis-Menten kinetics

$$l=\frac{v_{max}\cdot \left[C_{i,j}\right]}{K_{M}+\left[C_{i,j}\right]}$$ (2)

where *v*_*max*_ represents the maximal uptake rate and *K*_*M*_ the Michaelis-Menten constant, which can be obtained from public databases [65] or experimental data. The lower bound is constrained because exchange reactions are defined from the inside to the outside.

By default, FBA is used to calculate the metabolic fluxes given the metabolite concentrations of the local grid cells. Since most metabolic models are undetermined by having more reactions than metabolites, alternative optimal solutions (different flux distributions with the same objective value) occur during the simulations. To deal with this issue, we devised several alternatives to standard FBA calculations, which can be chosen by the user. For instance, parsimonious FBA can be used to minimize the total flux through all reactions of a metabolic model. In this case, the primary objective (e.g. biomass) is optimized first and afterwards a secondary objective (total flux) is minimized using the first optimal objective value as a constraint. The second optimization acts as a proxy for minimal enzyme usage to simulate a more realistic behavior of cells in the exponential growth phase [59]. Additionally, the secondary objective can be chosen as a single reaction, which is picked randomly for each individual in each optimization, while enforcing the same biomass objective, pre-computed by FBA. The randomization of alternative optimal solutions can also be performed on the level of exchange reactions exclusively to get a better representation of secreted and consumed metabolites. The resulting flux distribution of the respective simulation strategy is then used to calculate and update the secretion or uptake for each individual in each simulation step. The linear programming problems can be solved using different solvers, such as GLPK [66], CLP [67], CPLEX [68], and Gurobi [69].

Based on the resulting FBA solution for each individual, exchange fluxes are used to update metabolite quantities [*C*] in each grid cell. Moreover, the biomass *B*_*t*_ accumulated by an individual at time step *t* is updated according to an exponential growth model utilizing the optimal biomass yield *v*_*biomass*_ computed by FBA with

$$B_{t+1}=B_{t}\cdot v_{biomass}+B_{t}$$ (3)

for each individual in each time step. The initial biomass (*B*_0_) is selected according to the reported and experimentally determined median dry weight of one cell (Table 3). If multiple individuals are inserted in the environment, then a normally distributed random value is assigned to each individual, using the median and cell dry weight deviation (Table 3) as parameters for the normal distribution. When the total biomass of an individual reaches a duplication threshold, a daughter cell is spawned and placed at a free position in the Moore neighborhood (i.e. all surrounding grid positions in the direct neighborhood). The duplication threshold was chosen according to the experimentally determined maximum dry weight (Table 3), which represents the largest observed dry weight of one bacterial cell. To restrict growth to physiological feasible conditions, the accumulation of biomass is limited to 50% above the maximal cell weight. During optimization the upper bound of the objective function is set accordingly. If the biomass of an individual falls below a defined growth threshold, the corresponding individual dies (i.e. it is removed from the grid cell). If lysis is enabled, the biomass components can diffuse to neighboring grind cells. The growth threshold was chosen according to the experimentally determined minimum dry weight (Table 3), which represents the smallest observed dry weight of one bacterial cell.

**Table 3:** Default parameters of BacArena with references and the name of the variable set for the respective function.

Description	Variable	Function/Class	Value	Unit	Bionumber [72]	Ref
Cell space occupation	cellarea	Organism	4.42	*μm*^2^	105026	[73]
Maximal dry weight	cellweigth	Organism	1.172	*pg*	106615	[74]
Minimal dry weight	growthlimit	Organism	0.083	*pg*	106615	[74]
Biomass decrease	deathrate	Organism	0.210	*pg*	-	[74]
Median cell dry weight	cellweight_mean	Organism	0.489	*pg*	-	[74]
Dry weight deviation	cellweight_sd	Organism	0.132	*pg*	-	[74]
Oxygen diffusion (aqueous)	difspeed	Substance	20 × 10^−6^	*cm*^2^ *s*^−1^	104440	[64]
Glucose diffusion (aqueous)	difspeed	Substance	6.7 × 10^−6^	*cm*^2^ *s*^−1^	104089	[64]
Oxygen diffusion (biofilm)	difspeed	Substance	12 × 10^−6^	*cm*^2^ *s*^−1^	-	[64]
Glucose diffusion (biofilm)	difspeed	Substance	1.675 × 10^−6^	*cm*^2^ *s*^−1^	-	[64]
Glucose uptake Km	Km	setKinetics	0.01	*mM*	-	[75]
Glucose uptake Vmax	vmax	setKinetics	7.56	*mmol g*^−1^ *h*^−1^	-	[75]

Movement is implemented as a random walk of individuals using unoccupied grid positions in the Moore neighborhood. Different movement velocities can be imposed by setting the number of grid positions to which an individual can move. Individuals can also perform chemotaxis by moving towards a concentration gradient of a particular metabolite of interest. Diffusion of the metabolite concentration [*C*] in the two-dimensional *x*, *y* environment is implemented using Fick’s second law of diffusion which in two dimensions reads

$$\frac{\partial [C]}{\partial t}=D\cdot \left(\frac{\partial^{2}\left[C\right]}{\partial x^{2}}+\frac{\partial^{2}\left[C\right]}{\partial y^{2}}\right)\;\;\;$$ (4)

where $D\in {\mathbb{R}}^{s}$ is a vector of diffusion constants. Zero-gradient boundary conditions are set to ensure mass conservation. The diffusion model is defined using the R package *ReacTran* [70] and is solved by the integrator *lsodes* (R package *deSolve* [71]). Additional diffusion functionalities, such as advection or different boundary conditions, are available and additional ones can be implemented with *ReacTran*.

To analyze population heterogeneity in terms of the metabolic turn-over, we defined metabolic phenotypes *p* by

$$p=\left\{\begin{array}{cc} \;\;\;1, & \text{if}\;v_{ex}>\;\;\;\theta .\\ -1, & \text{if}\;v_{ex}<-\theta .\\ \;\;\;0, & \text{otherwise}. \end{array}\right.$$ (5)

according to an adjustable threshold *θ* (default value is *θ* = 10^−6^) and considering the exchange reaction flux *v*_*ex*_ of each individual. The metabolic phenotypes represent the metabolic signature of all secreted and consumed metabolites for each individual. The metabolic phenotypes track the metabolism of each individual during each simulation step and thus indicate how each microbial cell changes the environment and interacts with other species. In addition, BacArena provides a range of different data analysis techniques within the R environment to investigate the emergence of complex phenotypes on the population level (see reference manual in S2 Text).

### Parameters, units, and integration of experimental data

BacArena permits fine tuning of simulations through adjustment of parameters which are incorporated in the different classes (S1 Fig). The default parameters of BacArena are taken from various experimental data sets (Table 3). Based on the user defined length of the environment dimensions (in *cm*) and the number of grid cells, these parameters are automatically adjusted to represent physically meaningful results. Given the corresponding size of a grid cell (*cm*^2^) and the occupied space of the organism of interest (*μm*^2^), the maximal number of individuals per grid cell is computed and the maximum possible biomass per grid cell is calculated accordingly. Metabolite concentrations are integrated by converting molar concentrations (in *mM*) into metabolite amounts per grid cell based on the above defined geometry. Fluxes are calculated in *fmol* ⋅ (*pg*_*dryweight*_ *h*)^−1^.

### Syntrophic two-species community model

Manual curated genome-scale metabolic models were retrieved for the hydrogen producing bacterium *Clostridium beijerinckii* [35] and the methanogenic archaeon *Methanosarcina barkeri* [36]. The *M. barkeri* model was modified to ensure methane production with hydrogen and carbon dioxide by blocking the uptake of acetate and only allowing unidirectional uptake of hydrogen, hydrogen sulfide, and sulfur trioxide. The *C. beijerinckii* model was modified to block the secretion of acetate in order to ensure hydrogen production. To model metabolic exchanges between the microbes and compare the results of BacArena, we performed the simulations with our method and COMETS [24]. For both methods, simulations were carried out on a 100 times 100 grid environment for 24 hours. In both setups, a minimal medium was added to the environment with 1 mmol of glucose per grid position, carbon dioxide, and several co-factors (4 aminobenzoate, cobalt, nicotinic acid, water, protons, ammonium, nickel, phosphate, sulfur trioxide, cysteine, and sulfate). To ensure the growth of *M. barkeri* before *C. beijerinckii* produces a sufficient concentration of hydrogen, an initial amount of 10^−10^ mmol hydrogen was added to each grid position. The diffusion of metabolites was calibrated to the standard diffusion of glucose (Table 3). For COMETS the diffusion was executed one time per iteration to create a similar setting as in BacArena.

### *Pseudomonas aeruginosa* single-species biofilm model

Biofilm formation of *Pseudomonas aeruginosa* was simulated using the genome-scale reconstruction iMO1056 [76] retrieved from [25]. The reconstruction was modified to enable lactate fermentation (see S1 Text, S1 File). All growth parameters were set to default values (Table 3). The environment was initiated to represent one individual per grid cell and 100 × 100 grid cells, and therefore defining the spacial extent by 0.025*mm* × 0.025*mm*. Simulations were repeated ten times. For the starting condition, 900 individuals (9% inoculation) were placed into the center of the environment. Minimal medium, as described in [25], was used for each grid cell (S1 Table). 50 *μM* of glucose were added and all other metabolites of the minimal medium were initialized with a concentration of 100*μM*. Glucose uptake of each individual (i.e. *P. aeruginosa metabolic model*) was constrained according to Michaelis-Menten kinetics based on published values (Table 3). All remaining exchange reactions were unconstrained. Metabolites were allowed to diffuse freely with particular diffusion rates for gaseous and organic compounds in biofilms (Table 3). The simulation was performed for 48 time steps totaling to a simulation time of 2 days. The code and the model to reproduce the results of the simulations is provided in S1 and S2 Files. To model the influence of nitrate as additional electron acceptor, we used the results of the first 20 hours to resume the simulation after adding 0.01*mM* of nitrate. All simulations were performed using pFBA to generate the flux distributions of each individual.

### Integrated multi-species model of the human gut

A model for the human gut was assembled using seven recently reconstructed genome-scale metabolic models of human gut bacteria [77]. In this study, the models were manually curated and checked using published experimental data. The bacterial species were selected according to their relevance and abundance within the human gut microbiota to represent a simplified human intestinal microbiota (SIHUMI) [47]. The following microbial reconstructions were used *Anaerostipes caccae* DSM 14662, *Bacteroides thetaiotaomicron* VPI-5482, *Blautia producta* DSM 2950, *Escherichia coli* str. K-12 substr. MG1655, *Clostridium ramosum* VPI 0427, DSM 1402, *Lactobacillus plantarum* subsp. plantarum ATCC 14917, *Bifidobacterium longum* NCC2705, and *Akkermansia muciniphila* ATCC BAA-835. The models used for the simulations are available on vmh.uni.lu as well as S2 File.

Growth parameters and movement were set to the default values (Table 3) and the environment was initialized with a 100 × 100 grid corresponding to a side length of 0.025*mm*. Simulations were repeated five times, each time simulating 16*h* with time steps of 1*h*. In a first condition, the intestinal lumen was initialized with 200 individuals of each species in an environment which was devoid of mucin glycans. All remaining metabolites were set to a concentration of 0.1 *μM* except essential nutrients They were set to 1 *μM* to ensure that all bacteria were able to grow. The essential metabolites were determined using flux variability analysis [78] on all unbounded exchange reactions for each metabolic model, while enforcing a minimal biomass rate of 0.01*h*^−1^. The exact diet definition with the predicted essential metabolites can be found in S2 Table. To investigate the importance of spatial concentration gradients, we devised a second condition, in which mucus glycans (1 *μM*) were added as a linear gradient with decreasing concentrations from the bottom to the middle of the environment. Metabolites were allowed to diffuse according to diffusion rates for gaseous and organic compounds in aqueous solutions [64]. Mucin glycans were not allowed to diffuse, since they are known to be tightly associated with the epithelium in form of a mucous layer [79]. The code and the models to reproduce the results of the simulations are provided in S3 and S4 Files. All simulations were performed using pFBA to generate the flux distributions of each individual.

## Supporting information

S1 Text Tutorial for BacArena. This tutorial includes a basic hands-on description of all main classes and functions of BacArena.
(PDF)

S2 Text Reference manual of BacArena. All methods and parameters are explained with words and example codes in the documentation.
(PDF)

S3 Text *P. aeruginosa* single-species biofilm. Documentation of changes in metabolic model of *P. aeruginosa* and additional figures from replicates.
(PDF)

S1 File R Data file of modified *P. aeruginosa* model. Metabolic model of *P. aeruginosa* used in simulation.
(ZIP)

S2 File R script to reproduce *P. aeruginosa* simulation. This R script reproduces the biofilm simulation of *P. aeruginosa* simulation (to be used with S1 File).
(R) Click here for additional data file.

S3 File R Data file with all 7 species used for the gut simulation. Metabolic models of *A. caccae*, *B. thetaiotaomicron*, *B. producta*, *E. coli*, *C. ramosum*, *L. plantarum*, *B. longum*, and *A. muciniphila* used for the simulation of a simplified human gut model.
(ZIP)

S4 File R script to reproduce gut simulation. The R script can be used to reproduce the gut-community simulation (needs models from S2 file).
(R) Click here for additional data file.

S1 Table Table with the defined diet for *Pseudomonas aeruginosa* biofilm model. Table of all exchange reactions with their respective concentrations that were added to the environment.
(CSV)

S2 Table Table with the defined diet for the gut model. Table of all exchange reactions of the defined essential metabolites, mucus glycans, and remaining metabolites with their respective concentrations.
(CSV)

S1 Fig Class diagram of all main classes, functions, and variables in BacArena. Simplified class diagram displaying the inheritance hierarchy.
(TIF)

S2 Fig Comparison of *P. aeruginosa* phenotypes growth curve. For each phenotype (P2,P3,…,P9) of the *P. aeruginosa* biofilm simulation the time curves for all replicates are shown. While the overall dynamics were stable, the occurrences of P3, P7 and P8 showed some minor variance.
(TIF)

S3 Fig Influence of the addition of nitrate on *P. aeruginosa* biofilm growth. Alternative scenario of *P. aeruginosa* biofilm simulation with 0.1 *mM* nitrate added after 20 hours simulation time. **A** Spatial distribution of phenotypes and nitrate. The presence of nitrate after 20 hours was accomplished by a new nitrate consuming phenotype P11. B Comparison of phenotypes. **C** Time curve of core metabolites. The addition of nitrate after 20 hours lead to further glucose usage and CO_2_ production. The former produced acetate and succinate were used again. **D** Phenotypes growth curve. After the addition of nitrate, the metabolic inactive phenotype P1 vanished and the new nitrate consuming phenotype P11 emerged.
(TIF)

S4 Fig Growth curves and metabolite concentrations for the simplified human microbiota (SIHUMI) under different optimization strategies. The first row represents the species growth and the second row the concentration change of the 25 most variable metabolites. The first columns shows a default flux balance analysis and the second column the optimization of a random exchange reaction as a secondary objective. The curve range shows a standard deviation of 10 replicate simulations each simulating 16 hours.
(TIF)
