# Supplementary material for: BacArena: Individual-based metabolic modeling of heterogeneous microbes in complex communities
Source: PLoS Comput Biol. 2017 May 22;13(5):e1005544. doi: 10.1371/journal.pcbi.1005544 (PMC5460873; doi:10.1371/journal.pcbi.1005544)
Supplement: S1 Text — This tutorial includes a basic hands-on description of all main classes and functions of BacArena. (PDF) [file pcbi.1005544.s001.pdf]

BacArena - An Agent-Based Modeling Framework for Cellular Communities

Eugen Bauer and Johannes Zimmermann

2016-07-20

Introduction to BacArena

Microbial communities are essential for global ecosystems and human health. Computational modeling of microbial consortia is thus a major goal in systems biology and microbial ecology. BacArena is a project to simulate bacterial behavior in communities. A lot of progress is done in the last years to create genome wide metabolic reconstructions of certain organisms, which open a wide field of mathematical analysis. One of this new methods is flux balanced analysis (FBA) to estimate optimal metabolic fluxes under certain constraints. Some of these models are available in an exchangeable format (SBML) and can be found in http://systemsbiology.ucsd.edu/InSilicoOrganisms/OtherOrganisms. The idea of this project is to use this existing reconstructions and put them in a spatial and temporal environment to study their possible interactions. This is achieved by the combination of agent based modeling with FBA. Each bacterium is considered as an agent with individual states, own properties and rules to act. Agents are located on a grid where they can move and interact via metabolic exchanges computed by FBA. The starting point for our project is curiosity of what could be done with this huge models. We just throw those models into an arena to see what kind of actions will evolve.

Getting started

Simple simulations with the *Escherichia coli* core metabolic model

First we have to load the installed package in the workspace with

```
library("BacArena")
```

Next we set-up the *Escherichia coli* core metabolic model

```
data("Ec_core")
```

The model is already integrated in the sybil R package by default. Alternatively you can also load your own model of interest with commands of the sybil and libSBML. After we loaded the model, we convert it into an object of class Bac by calling its constructor

```
bac <- Bac(Ec_core)
```

```
## Loading required package: glpkAPI
## using GLPK version 4.60
```

Now we have to set up an environment in which the organisms can interact

```
arena <- Arena(n=20, m=20)
```

Here, by default we chose 20 times 20 as the grid size of the environment. If not specified, the physical area of the environment is set automatically, in this case it is 0.005cm times 0.005cm. Details could be checked by

```
arena
```

Next we want to put our created organism in its environment by

```
arena <- addOrg(arena,bac,amount=20)
```

With this command we added 20 individuals of our bacterium. Now we can add the substances to the environment

```
arena <- addDefaultMed(arena, bac)
arena <- addSubs(arena, smax=0.5, mediac="EX_glc(e)", unit="mM")
```

We added the default medium which was preset in the model together with 0.5 mM glucose. We can check which substances were added at which amount by calling the *arena* object again

```
arena
```

```
## [1] "substances with 322.5 fmol per gridcell:"
## [1] "EX_glc(e)"
##
## [1] "substances with 1000 fmol per gridcell:"
## [1] "EX_co2(e)" "EX_h(e)"   "EX_h2o(e)" "EX_nh4(e)" "EX_o2(e)"  "EX_pi(e)"
##
## [1] "arena grid cells: 20 x 20"
## [1] "arena grid size [cm]: 0.005 x 0.005"
## [1] "dimension of one grid cell [cm]: 0.00025 x 0.00025"
## [1] "area of one grid cell [cm^2]: 6.25e-08"
## [1] "flux unit: mmol/(h*g_dw)"
## [1] "1 mM in arena correspons to mmol/grid_cell: 6.25e-10"
## [1] "Arena of size 20x20 with 20 organisms of 1 species."
```

Finally, we can start *in silico* experiment simulating 10 hours of *E. coli* growth with

```
eval <- simEnv(arena,time=12)
```

During simulation, some basic statistics about growth will be printed. The object eval stores all 10 simulation steps. After we retrieve the eval object we can start analyzing the data. To get a simple overview about what happened we can look at substances having high variation

```
getVarSubs(eval)
```

```
## EX_h2o(e) EX_co2(e)  EX_o2(e)   EX_h(e) EX_glc(e) EX_nh4(e)  EX_pi(e)
## 2.974e+10 1.726e+10 1.558e+10 1.281e+10 3.833e+09 9.464e+08 4.307e+08
##  EX_ac(e)
## 2.205e+06
```

To pick one substance and check its time series

```
getSubHist(eval, "EX_glc(e)")
```

```
##      1      2      3      4      5      6      7      8      9     10
## 129000 128799 128412 127417 124230 108065  55682   6277      0      0
##     11     12     13
##      0      0      0
```

To start with graphical analysis let's investigate the growth curve in combination with the substance variations

```
par(mfrow=c(1,2))
plotCurves2(eval, legendpos = "topleft")
```

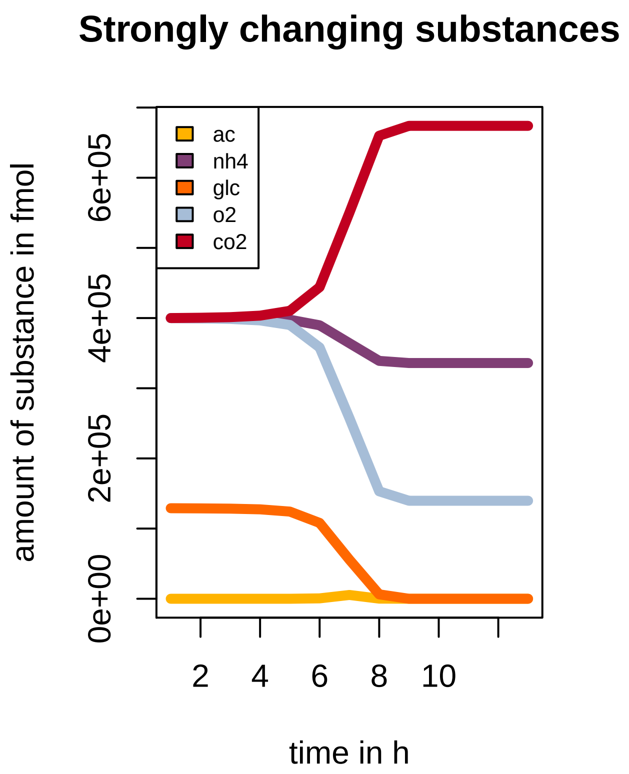

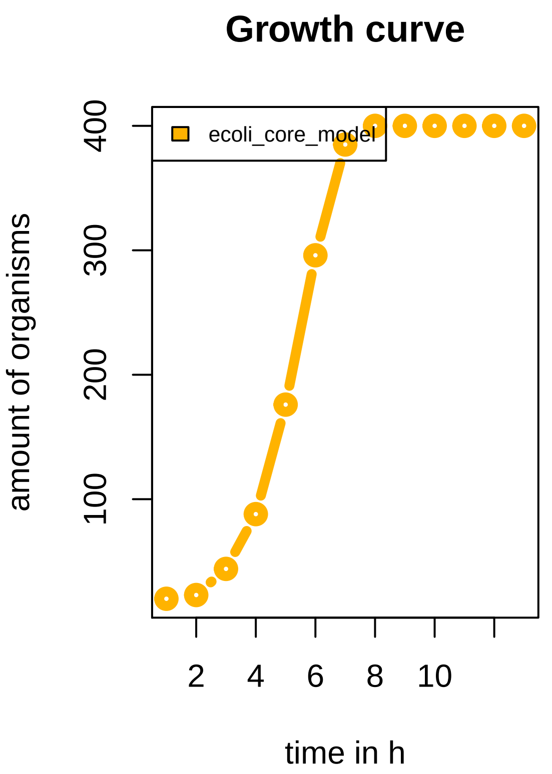

Spatial and temporal changes can be shown via

```
par(mfrow=c(2,5))
evalArena(eval, show_legend = FALSE, time=1:10)
```

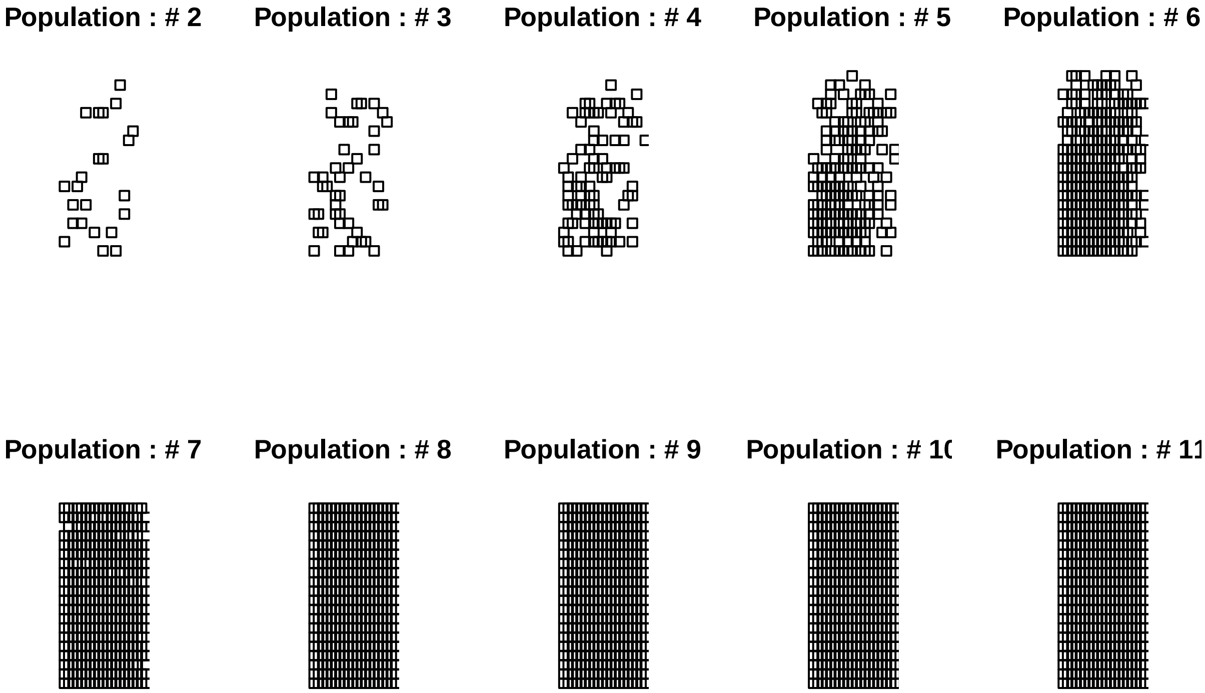

This will produce multiple plots one by one for each simulation step with the spatial structure of the population (black dots represent individuals).

Simulation of multiple organisms

Now we want to multiple organisms or organism types in the environment. For this we create two different types of the *Escherichia coli* core metabolic model: A wild type *E. coli* and an auxotrophic mutant which is unable to use aerobic respiration.

```
bac1 <- Bac(Ec_core,type="ecoli_wt")
```

Now we create the auxotrophic mutant by using basic commands of the sybil package.

```
ecore_aux <- changeBounds(Ec_core, "EX_o2(e)",lb=0)
bac2 <- Bac(ecore_aux,type="ecoli_aux", setExInf=FALSE)
```

Again we set up an environment and insert organisms and substances

```
arena <- Arena(n=30, m=30)
arena <- addOrg(arena,bac1,amount=20)
arena <- addOrg(arena,bac2,amount=20)
arena <- addDefaultMed(arena, bac1)
arena <- addSubs(arena, smax=0.5, mediac="EX_glc(e)", unit="mM")
eval <- simEnv(arena,time=10)
```

Here we put the both organism types we created next to each other (given by their x position) in the environment and then started the simulation for 10 time steps. Next we perform again all evaluation steps

```
plotCurves2(eval)
```

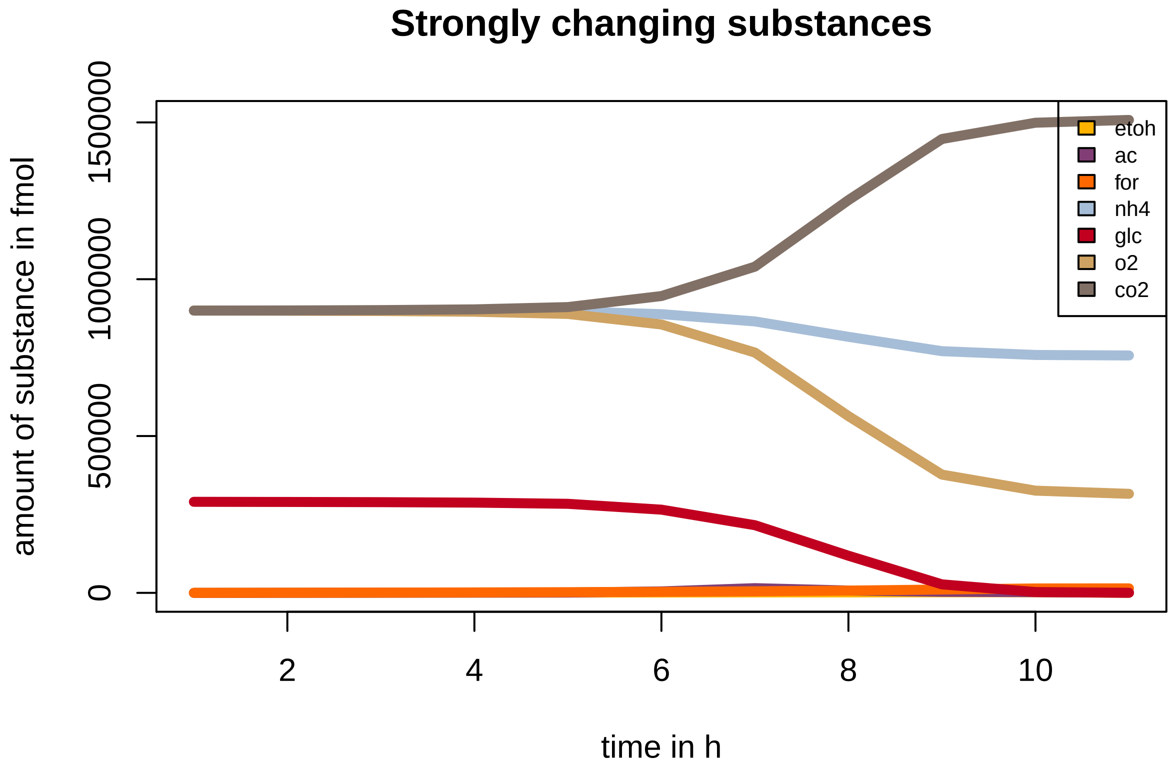

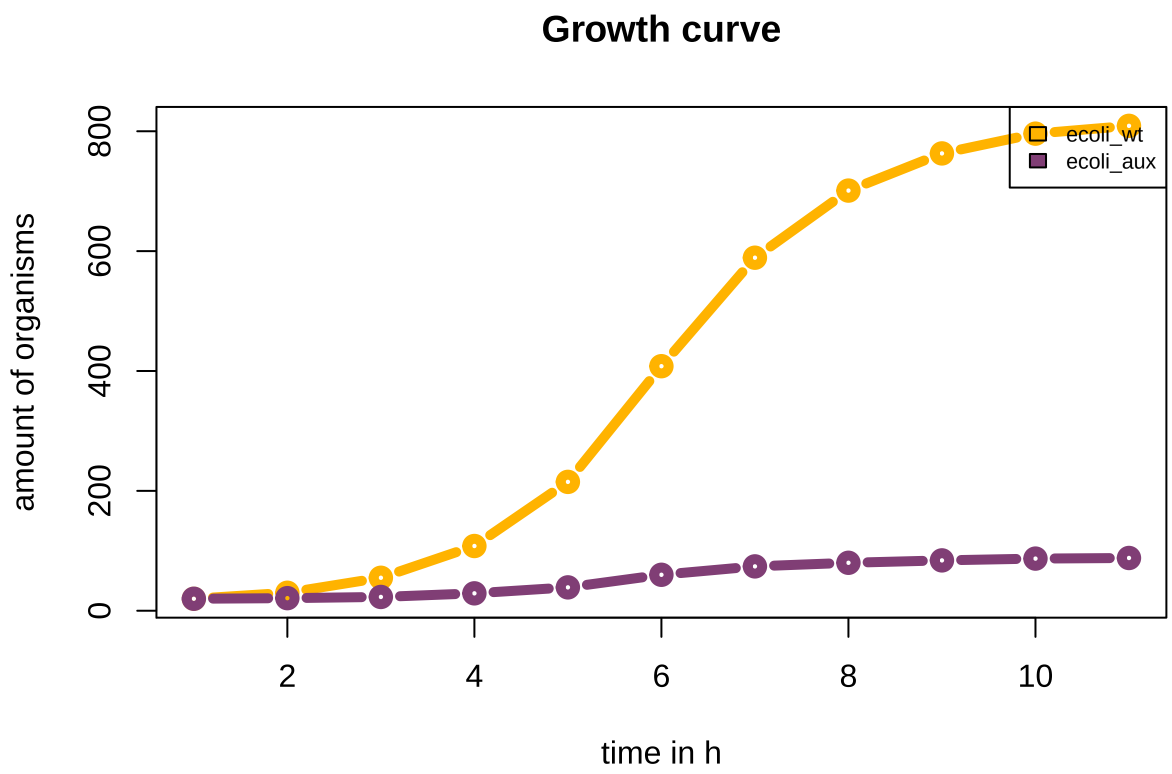

And the spatial pattern of the community with the substances glucose, acetate and oxygen:

```
par(mar=c(1,1,1,1))
evalArena(eval,c("Population","EX_glc(e)","EX_ac(e)","EX_o2(e)"),
          time=7)
```

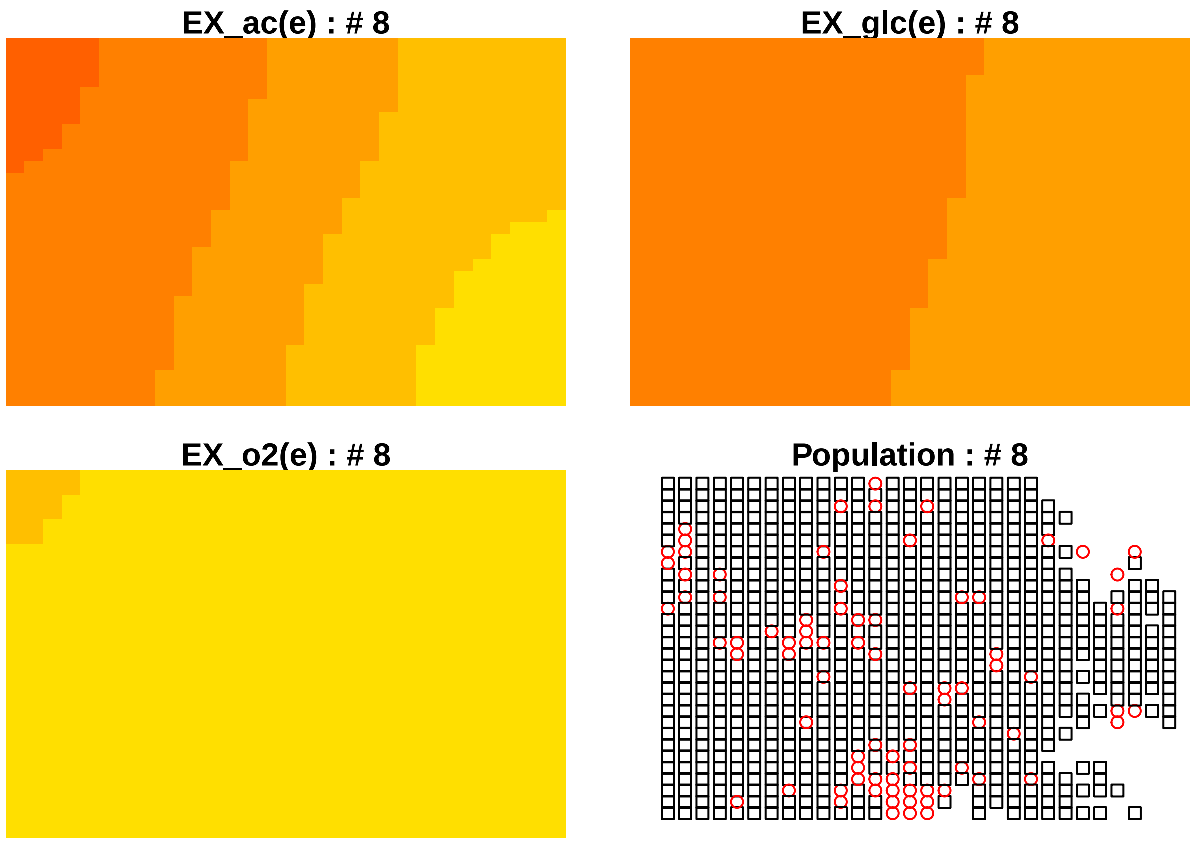

Here different point colors indicate the two different organism types. Finally we can have a look on phenotypes. The command will create a PCA plot with the similarity of the different phenotypes. If we are interested in the definition of the phenotypes we can also retrieve the original phenotype matrix

```
minePheno(eval)
```

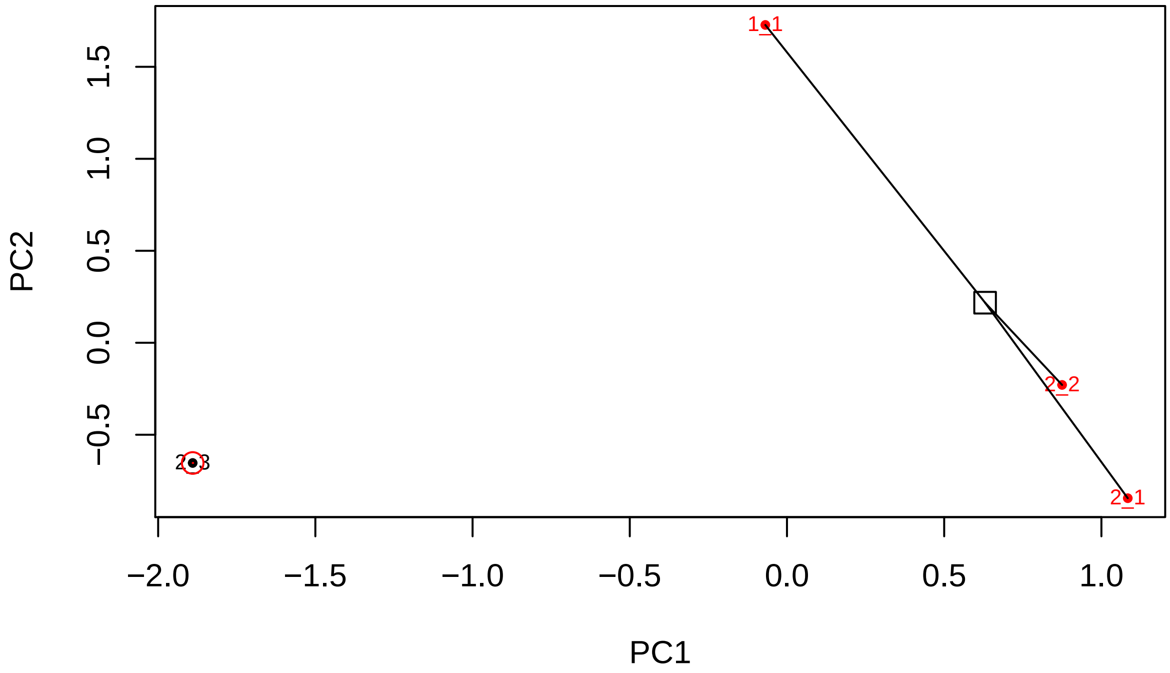

```
pmat <- getPhenoMat(eval)
pmat[,which(colSums(pmat)>0)]
```

```
##              EX_ac(e) EX_co2(e) EX_etoh(e) EX_for(e) EX_glc(e) EX_h(e)
## ecoli_wt.1          0         1          0         0         2       1
## ecoli_wt.2          2         1          2         0         2       1
## ecoli_wt.3          1         1          2         0         2       1
## ecoli_aux.1         1         2          1         1         2       1
##              EX_h2o(e) EX_nh4(e) EX_o2(e) EX_pi(e)
## ecoli_wt.1           1         2        2        2
## ecoli_wt.2           1         2        2        2
## ecoli_wt.3           1         2        2        2
## ecoli_aux.1          2         2        0        2
```

Based on these results we can see, that the auxotrophic organism type grows slower in general and uses just fermentation of glucose, whereas the the wild type can respire glucose with the aid of oxygen. We can also create customized microbial communities or multicellular systems by importing external SBML models using the readSBMLmod function in the sybilSBML package.

Advanved

Simulation with replicates

BacArena supports parallel computing, e.g., there is a parallelized *simEnv_par()*. Additionally, several simulations could be run in parallel to get replicates for better validation of the results. This could be done via the parallel package which supports parallelism in Linux, MacOS and Windows. Therefore, the basic BacArena methods, introduced in the last section, are used within a parallelized lapply

```
library(parallel)
cores <- ifelse(detectCores()>=2, 2, 1)
cl <- makeCluster(cores, type="PSOCK")
clusterExport(cl, "Ec_core")
simlist <- parLapply(cl, 1:cores, function(i){
  bac   <- BacArena::Bac(model=Ec_core)
  arena <- BacArena::Arena(n=20, m=20)
  arena <- BacArena::addOrg(arena, bac, amount=10)
  arena <- BacArena::addDefaultMed(arena, bac)
  sim   <- BacArena::simEnv(arena, time=5)
})
```

We can obtain a growth curve with standard deviation

```
p <- plotGrowthCurve(simlist)
p[[2]]
```

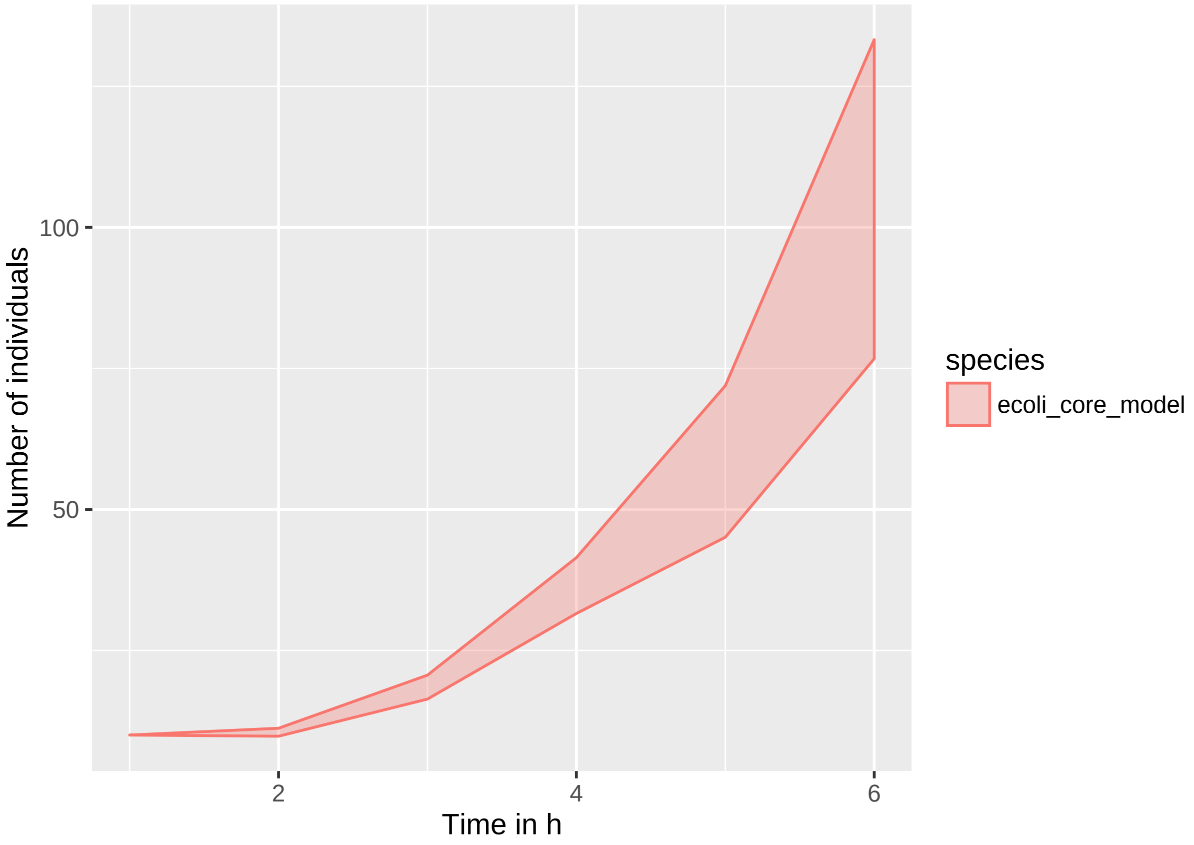

Additionally, there is substance time curve with standard deviation, too

```
p <- plotSubCurve(simlist)
p[[3]]
```

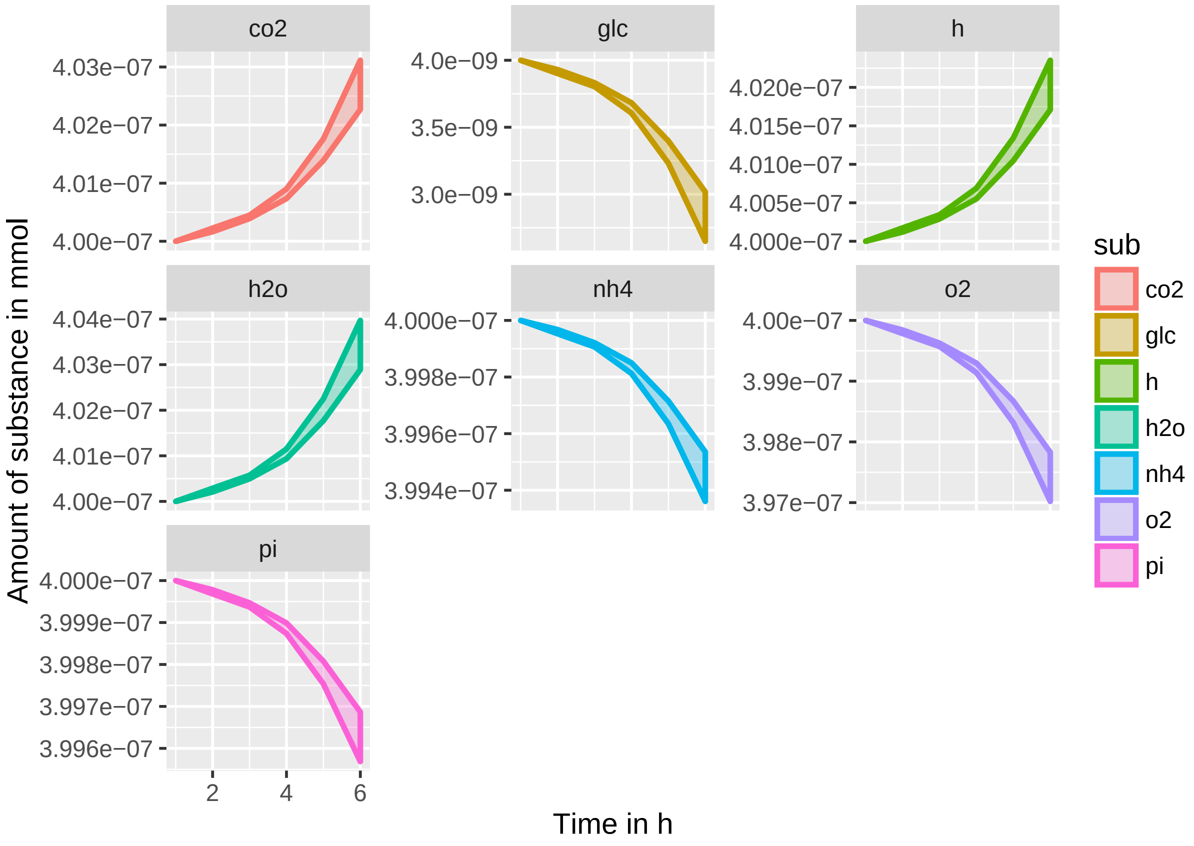

Community analysis

BacArena was developed with focus on microbial communities. Simulation results are stored in object of class *eval*. Therefore, simulation could be separated from analysis and simulation files easily exchanged. To test the available methods for community analysis, BacArena provides a sample simulation file containing a 10 hour simulation of 8 gut microbes (SIHUMI community) fed with a standard diet (Altromin 1310). The test data is called *sihumi_test*

```
data("sihumi_test")
eval <- sihumi_test
```

To get a summary of the growth, we start analysis by looking on the abundances of each species over time

```
p <- plotAbundance(eval)
p + ggplot2::scale_color_manual(values=colpal3)
```

```
## Scale for 'colour' is already present. Adding another scale for
## 'colour', which will replace the existing scale.
```

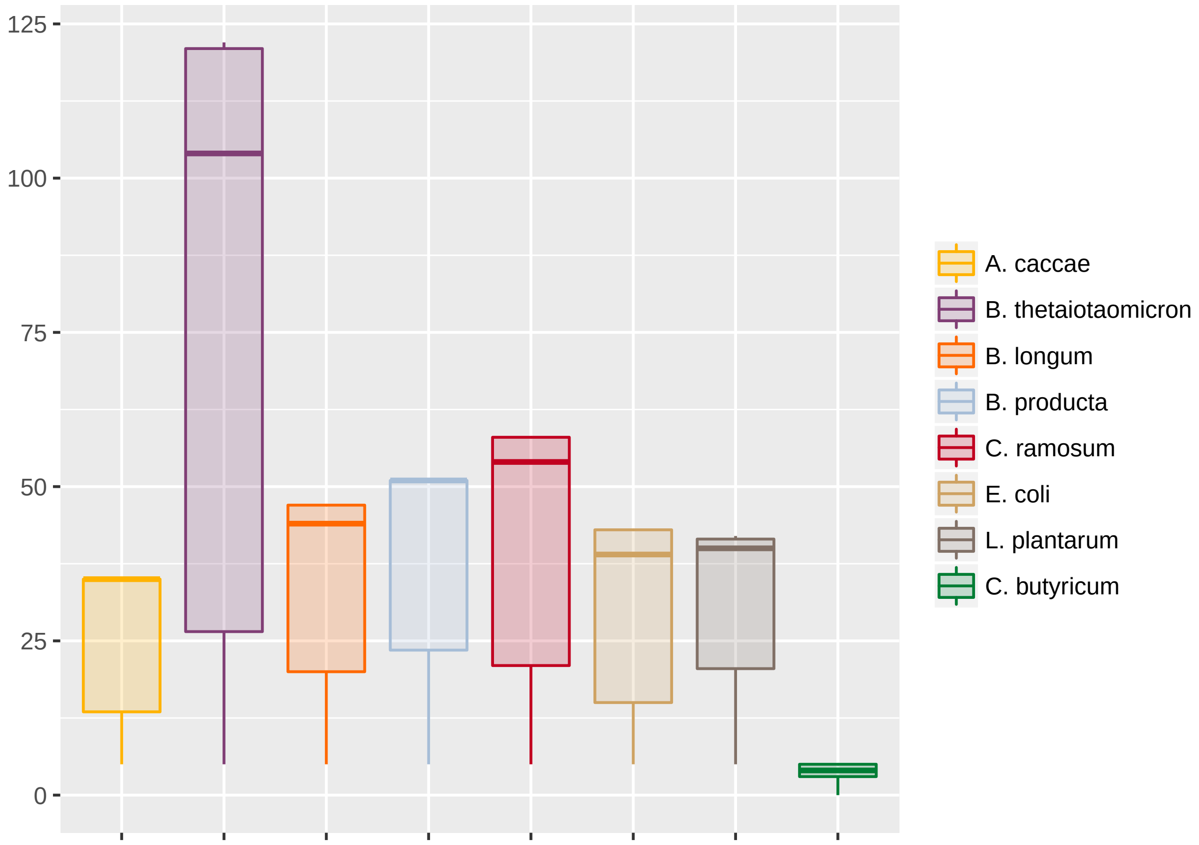

The methods *plotAbundances()* returns a ggplot object which can be modified, e.g., to use different colors.

Quit useful is to investigate the metabolic activity of all organisms. We obtain a figure with uptake (negative values) and production (positive values) patterns of substances which changed most during simulation by

```
p <- plotSpecActivity(eval)
p[[2]]
```

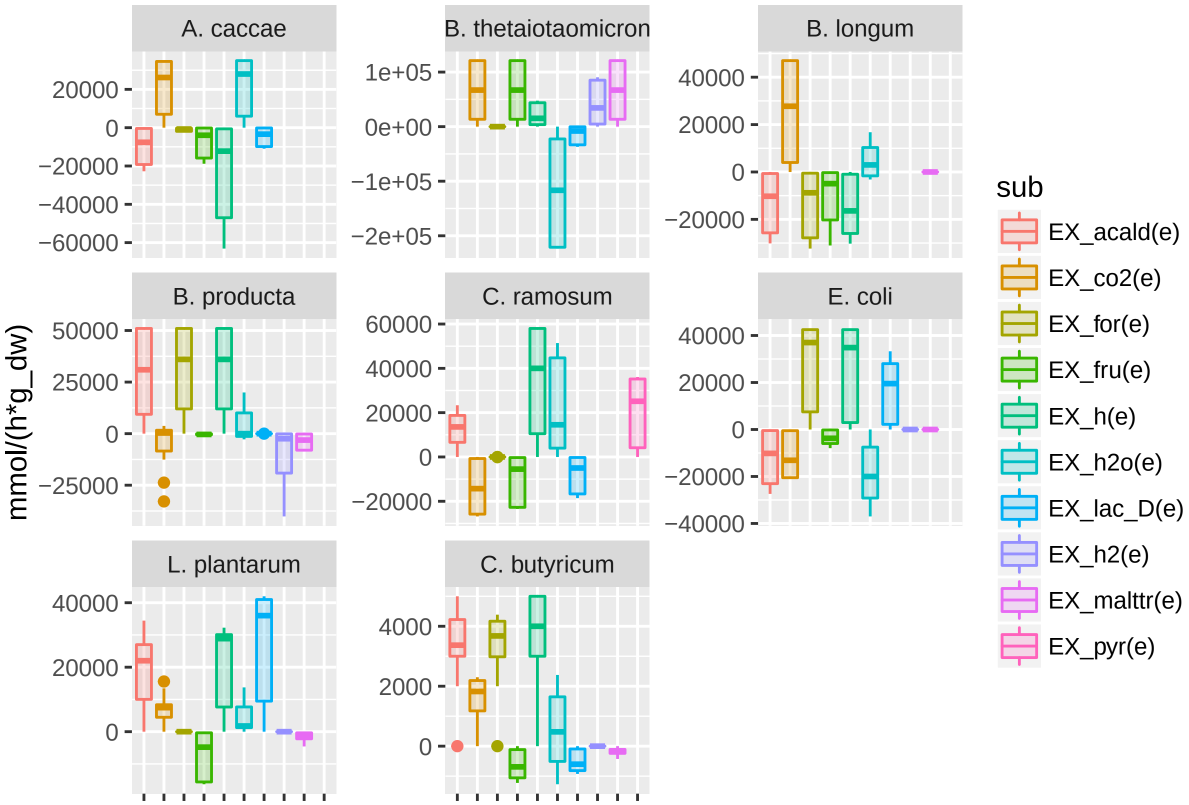

We see for example that acetaldehyde is produced by *B. producta*, *C. ramosum*, and L. *plantarum* and is used by all other species except *B. thetaiotaomicron.* Furthermore, the concentration of lactate is increased by *Lactobacillus plantarum* and again lactate supports growth of other species such as *B. thetaiotaomicon.* Moreover, we can identify *B. producta* and *E. coli* as formate producers which is a short chain fatty acid (SCFA).

We can also try to find cross feeding relationship automatically

```
g <- findFeeding3(eval, time = 10, mets = c("EX_acald(e)", "EX_lac_D(e)") )
```

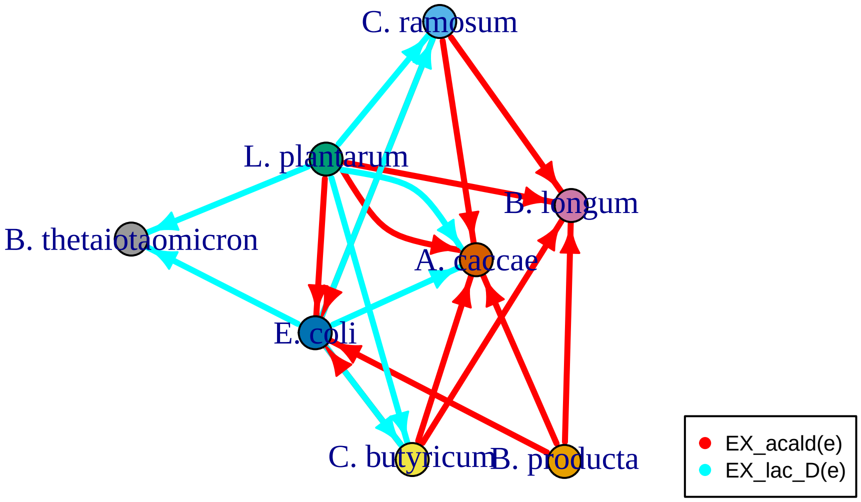

This graph can be interpreted as an ecological food web.

The main components of the diet are protein, starch, sucrose, oil, cellulose. Afterwards, we study the consumption of diet components (without amino acids) per species

```r
p <- plotSubUsage(eval, subs = c("EX_sucr(e)", "EX_amylose300(e)", "EX_amylopect900(e)",
                                 "EX_cellb(e)", "EX_hdca(e)", "EX_ocdca(e)"))
p[[2]]
```

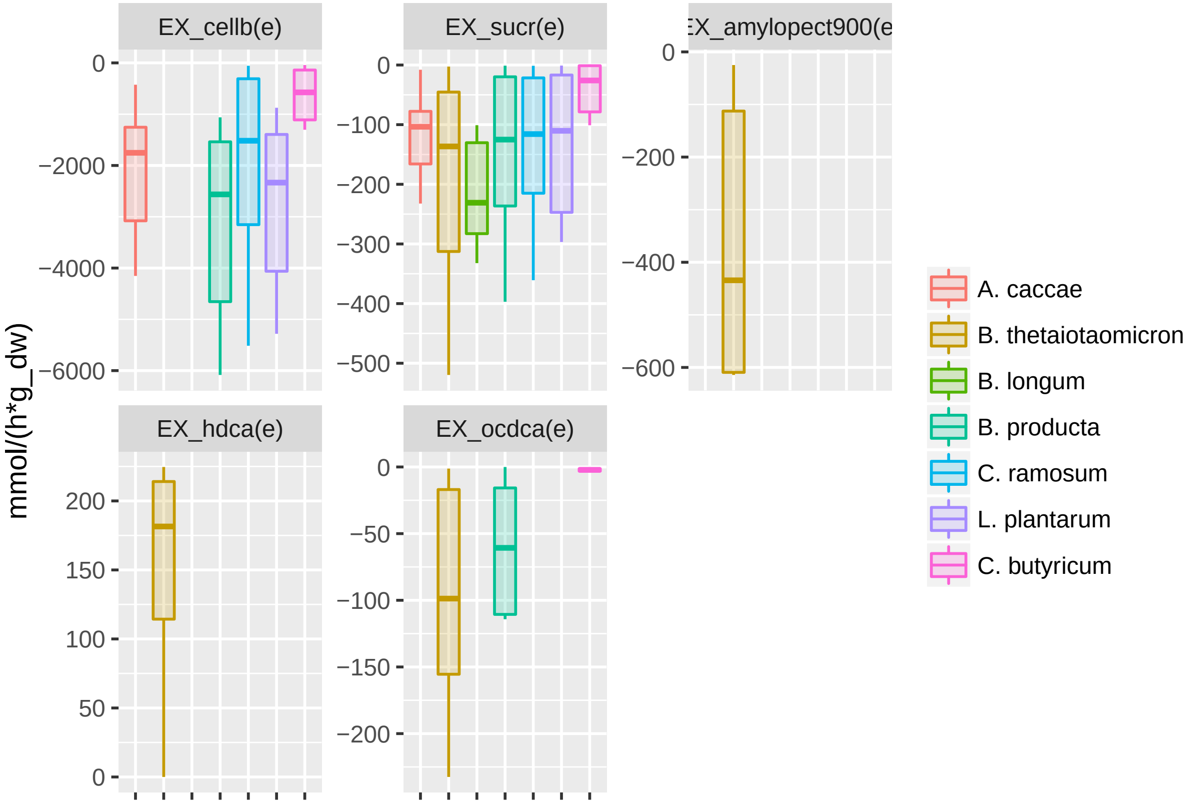

Whereby sucrose can be used by all species, starch is only metabolized by *B. thetaiotaomicron* and fatty acids additionally by *B. producta* and to a minor part also by *C. butyricum*. On the other hand, in case of cellulose all species except *B. thetaiotaomicron* and *B. longum* show absorption.

Due to the underlying method, it is possible to compute substances whose increased availability would contribute most to growth. We can use this to detect possible substrate or cofactor shortage

```r
p <- plotShadowCost(eval, 6)
p[[2]]
```

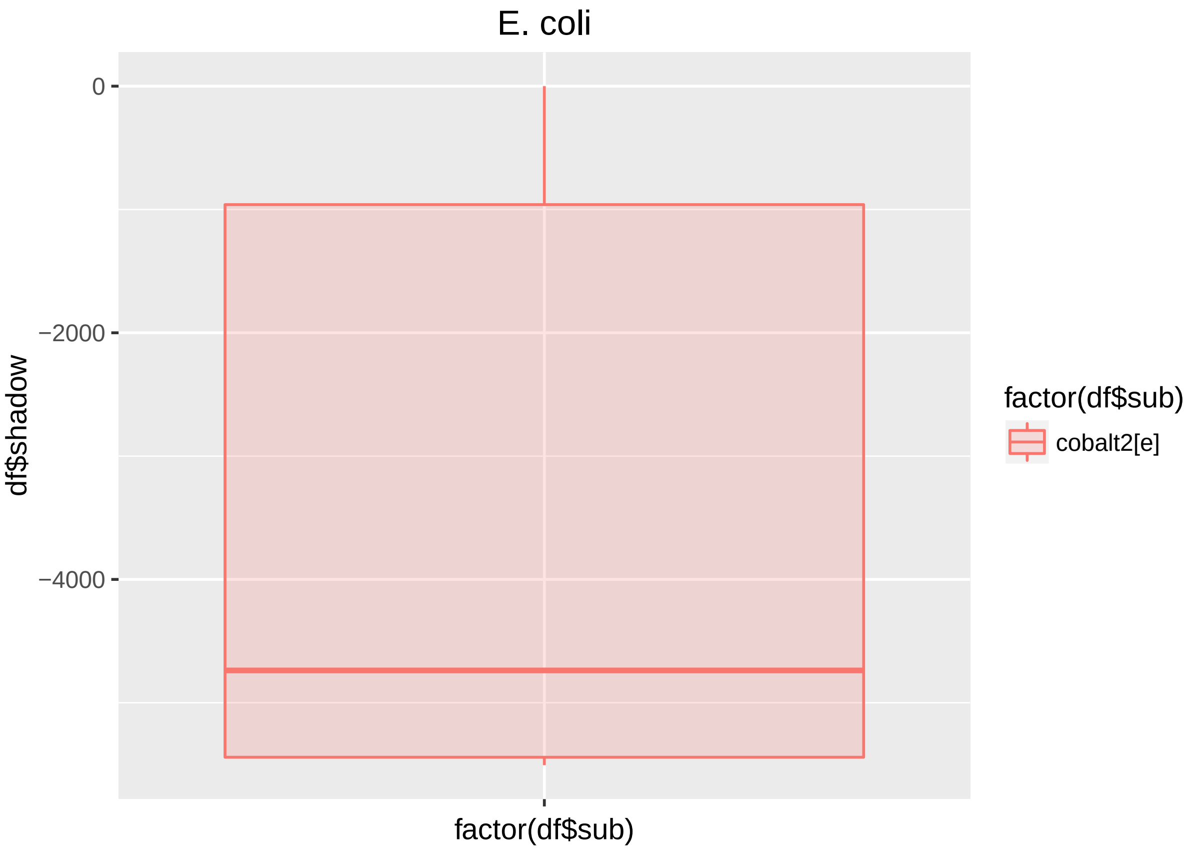

In case of *E. coli*, we find that a lack of cobalt is limiting its growth.
