# Supplementary material for: BacArena: Individual-based metabolic modeling of heterogeneous microbes in complex communities
Source: PLoS Comput Biol. 2017 May 22;13(5):e1005544. doi: 10.1371/journal.pcbi.1005544 (PMC5460873; doi:10.1371/journal.pcbi.1005544)
Supplement: S2 Text — All methods and parameters are explained with words and example codes in the documentation. (PDF) [file pcbi.1005544.s002.pdf]

Package ‘BacArena’

July 14, 2016

Title Modeling Framework for Cellular Communities in their Environments

Version 1.5.0

Author Eugen Bauer [aut],
Johannes Zimmermann [aut, cre]

Maintainer Johannes Zimmermann <j.zimmermann@iem.uni-kiel.de>

Description Can be used for simulation of organisms living in communities. Each organism is represented individually and genome scale metabolic models determine the uptake and release of compounds. Biological processes such as movement, diffusion, chemotaxis and kinetics are available along with data analysis techniques.

URL https://github.com/euba/BacArena

BugReports https://github.com/euba/BacArena/issues

Depends R (>= 3.0.0), sybil (>= 1.3.0), ReacTran (>= 1.4.2), deSolve (>= 1.12), Matrix (>= 1.2)

Imports igraph, methods, utils, stats, graphics, ggplot2, reshape2, glpkAPI, Rcpp

Suggests sybilSBML, knitr, rmarkdown

LinkingTo Rcpp, RcppArmadillo, RcppEigen

License GPL-3

VignetteBuilder knitr

RoxygenNote 5.0.1

LazyData true

NeedsCompilation yes

Repository CRAN

Date/Publication 2016-07-14 20:51:15

R topics documented:

addDefaultMed . . . 4
addEssentialMed . . . 4
addEval . . . 5
addOrg . . . 6
addSubs . . . 7
Arena-class . . . 8
Arena-constructor . . . 9
Bac-class . . . 9
Bac-Constructor . . . 10
BacArena . . . 10
cellgrowth . . . 11
changeDiff . . . 12
changeFobj . . . 13
changeOrg . . . 14
changeSub . . . 15
checkCorr . . . 16
checkPhen . . . 17
checkPhen_par . . . 18
chemotaxis . . . 18
constrain . . . 19
consume . . . 20
createGradient . . . 21
dat2mat . . . 22
diffuse . . . 23
diffusePDE . . . 23
diffuseR . . . 24
diffuse_par . . . 25
emptyHood . . . 26
Eval-class . . . 27
Eval-constructor . . . 27
evalArena . . . 28
extractMed . . . 29
findFeeding . . . 30
findFeeding2 . . . 31
findFeeding3 . . . 32
findInArena . . . 32
flushSubs . . . 33
getArena . . . 34
getCorrM . . . 35
getPhenoMat . . . 36
getPhenotype . . . 37
getSubHist . . . 38
getSublb . . . 38
getVarSubs . . . 39
growExp . . . 40
growLin . . . 40

growth . . . 41
growth_par . . . 42
Human-class . . . 42
Human-constructor . . . 43
lsd . . . 43
lysis . . . 44
minePheno . . . 45
move . . . 46
NemptyHood . . . 47
openArena . . . 48
optimizeLP . . . 48
Organism-class . . . 49
Organism-constructor . . . 50
plotAbundance . . . 51
plotCurves . . . 51
plotCurves2 . . . 52
plotFluxVar . . . 54
plotGrowthCurve . . . 54
plotInterNum . . . 55
plotPhenCurve . . . 55
plotPhenNum . . . 56
plotShadowCost . . . 56
plotSpecActivity . . . 57
plotSubCurve . . . 57
plotSubUsage . . . 58
plotSubVar . . . 59
plotTotFlux . . . 59
redEval . . . 60
reset_screen . . . 61
rmSubs . . . 61
selPheno . . . 61
setKinetics . . . 62
simBac . . . 63
simBac_par . . . 64
simEnv . . . 65
simEnv_par . . . 66
simHum . . . 67
statPheno . . . 68
stirEnv . . . 69
Substance-class . . . 70
Substance-constructor . . . 71
usd . . . 71

Index **72**

addDefaultMed *Add default medium of an organism to arena.*

Description

The generic function `addDefaultMed` uses the lower bounds defined in an organism's model file to compose minimal medium.

Usage

```
addDefaultMed(object, org)

## S4 method for signature 'Arena'
addDefaultMed(object, org)
```

Arguments

`object`	An object of class Arena.
`org`	An object of class Organism

addEssentialMed *Add minimal medium of an organism to arena.*

Description

The generic function `addEssentialMed` uses flux variability analysis to determine a essential growth medium components (eg. cofactors)

Usage

```
addEssentialMed(object, org)

## S4 method for signature 'Arena'
addEssentialMed(object, org)
```

Arguments

`object`	An object of class Arena.
`org`	An object of class Organism

addEval — *Function for adding a simulation step*

Description

The generic function addEval adds results of a simulation step to an Eval object.

Usage

```
addEval(object, arena, replace = F)

## S4 method for signature 'Eval'
addEval(object, arena, replace = F)
```

Arguments

object	An object of class Eval.
arena	An object of class Arena.
replace	A boolean variable indicating if the last simulation step should be replaced by the new simulation step arena.

Details

The function addEval can be used in iterations to manipulate an Arena object and store the results in an Eval object.

See Also

Eval-class and Arena-class

Examples

```
data(Ec_core, envir = environment()) #get Escherichia coli core metabolic model
bac <- Bac(Ec_core,deathrate=0.05,
           minweight=0.05,growtype="exponential") #initialize a bacterium
arena <- Arena(n=20,m=20) #initialize the environment
arena <- addOrg(arena,bac,amount=10) #add 10 organisms
arena <- addSubs(arena,40) #add all possible substances
eval <- simEnv(arena,5)
addEval(eval,arena)
```

addOrg *Add individuals to the environment*

Description

The generic function `addOrg` adds individuals to the environment.

Usage

```
addOrg(object, specI, amount, x = NULL, y = NULL, growth = NA)

## S4 method for signature 'Arena'
addOrg(object, specI, amount, x = NULL, y = NULL,
  growth = NA)
```

Arguments

object	An object of class Arena.
specI	An object of class Organism.
amount	A numeric number giving the number of individuals to add.
x	A numeric vector giving the x positions of individuals on the grid.
y	A numeric vector giving the y positions of individuals on the grid.
growth	A numeric vector giving the starting biomass of the individuals.

Details

The arguments `x` and `y` should be in the same length as the number of organisms added (given by the argument `amount`).

See Also

`Arena-class` and `Bac-class`

Examples

```
data(Ec_core, envir = environment()) #get Escherichia coli core metabolic model
bac <- Bac(Ec_core,deathrate=0.05,
           minweight=0.05,growtype="exponential") #initialize a bacterium
arena <- Arena(n=20,m=20) #initialize the environment
arena <- addOrg(arena,bac,amount=10) #add 10 organisms
```

addSubs — *Add substances to the environment*

Description

The generic function addSubs adds specific substances to the environment.

Usage

```
addSubs(object, smax = 0, mediac = object@mediac, difunc = "pde",
  difspeed = 6.7e-06, unit = "mmol/cell", add = TRUE)

## S4 method for signature 'Arena'
addSubs(object, smax = 0, mediac = object@mediac,
  difunc = "pde", difspeed = 6.7e-06, unit = "mmol/cell", add = TRUE)
```

Arguments

object	An object of class Arena.
smax	A numeric vector indicating the maximum substance concentration per grid cell.
mediac	A character vector giving the names of substances, which should be added to the environment (the default takes all possible substances).
difunc	A character vector ("pde","cpp" or "r") describing the function for diffusion.
difspeed	A number indicating the diffusion speed (given by number of cells per iteration).
unit	A character used as chemical unit to set the amount of the substances to be added (valid values are: mmol/cell, mmol/cm2, mmol/arena, mM)
add	A boolean variable defining whether the amount of substance should be summed or replaced

Details

If nothing but `object` is given, then all possible substrates are initilized with a concentration of 0. Afterwards, changeSub `can be used to modify the concentrations of specific substances.`

See Also

Arena-class and changeSub

Examples

```
data(Ec_core, envir = environment()) #get Escherichia coli core metabolic model
bac <- Bac(Ec_core,deathrate=0.05,
           minweight=0.05,growtype="exponential") #initialize a bacterium
arena <- Arena(n=20,m=20) #initialize the environment
arena <- addOrg(arena,bac,amount=10) #add 10 organisms
arena <- addSubs(arena,20,c("EX_glc(e)","EX_o2(e)","EX_pi(e)")) #add glucose, o2, pi
```

`Arena-class` *Structure of the S4 class "Arena"*

Description

Structure of the S4 class `Arena` to represent the environment in which Organisms and Substances interact.

Slots

`orgdat` A data frame collecting information about the accumulated growth, type, phenotype, x and y position for each individual in the environment.

`specs` A list of organism types and their associated parameters.

`media` A list of objects of class `Substance-class` for each compound in the environment.

`phenotypes` A list of unique phenotypes (metabolites consumed and produced), which occurred in the environment.

`mediac` A character vector containing the names of all substances in the environment.

`tstep` A number giving the time (in h) per iteration.

`stir` A boolean variable indicating if environment should be stirred.

`mflux` A vector containing highly used metabolic reactions within the arena

`shadow` A vector containing shadow prices of metabolites present in the arena

`n` A number giving the horizontal size of the environment.

`m` A number giving the vertical size of the environment.

`Lx` A number giving the horizontal grid size in cm.

`Ly` A number giving the vertical grid size in cm.

`gridgeometry` A list containing grid geometry parameter

`seed` An integer refering to the random number seed used to be reproducible

`scale` A numeric defining the scale factor used for intern unit conversion.

`models` A list containing Objects of class sybil::modelorg which represent the genome scale metabolic models

`occupyM` A matrix indicating grid cells that are obstacles

`sublb` A data matrix containing positions with amounts of substance for all organism

Arena-constructor — *Constructor of the S4 class* Arena-class

Description

Constructor of the S4 class Arena-class

Usage

```
Arena(Lx = NULL, Ly = NULL, n = 100, m = 100, ...)
```

Arguments

Lx	A number giving the horizontal grid size in cm.
Ly	A number giving the vertical grid size in cm.
n	A number giving the horizontal size of the environment.
m	A number giving the vertical size of the environment.
...	Arguments of Arena-class

Bac-class — *Structure of the S4 class "Bac"*

Description

Structure of the S4 class Bac inheriting from class Organism-class representing bacterial cells.

Slots

chem A character vector indicating name of substance which is the chemotaxis attractant. Empty character vector if no chemotaxis.

Bac-Constructor *Constructor of the S4 class* Bac-class

Description

Constructor of the S4 class Bac-class

Usage

```
Bac(model, chem = "", ...)
```

Arguments

model	model
chem	A character vector indicating name of substance which is the chemotaxis attractant. Empty character vector if no chemotaxis.
...	Arguments of Organism-class

Value

Object of class Bac-class

BacArena *BacArena: An Agent-Based Modeling Framework for Cellular Communities*

Description

The BacArena package provides six classes: Arena (subclass Eval), Organism (subclasses Bac, Human) and Substance. Accordingly there are three categories of important functions: Arena, Organism and Substance.

Arena functions

The Arena functions ...

Organism functions

The Organism functions ...

Substance functions

The Substance functions ...

`cellgrowth` *Function implementing a growth model of a human cell*

Description

The generic function `cellgrowth` implements different growth models for an object of class Human.

Usage

```
cellgrowth(object, population, j, occupyM, fbasol)

## S4 method for signature 'Human'
cellgrowth(object, population, j, occupyM, fbasol)
```

Arguments

`object`	An object of class Human.
`population`	An object of class Arena.
`j`	The number of the iteration of interest.
`occupyM`	A matrix indicating grid cells that are obstacles
`fbasol`	Problem object according to the constraints and then solved with `optimizeProb`.

Details

Linear growth of organisms is implemented by adding the calculated growthrate by `optimizeLP` to the already present growth value. Exponential growth of organisms is implemented by adding the calculated growthrate multiplied with the current growth calculated by `optimizeLP` plus to the already present growth value.

Value

Boolean variable of the `j`th individual indicating if individual died.

See Also

`Human-class`, `growLin` and `growExp`

changeDiff *Change substance concentration patterns in the environment*

Description

The generic function `changeDiff` changes specific substance concentration patterns in the environment.

Usage

```
changeDiff(object, newdiffmat, mediac)

## S4 method for signature 'Arena'
changeDiff(object, newdiffmat, mediac)
```

Arguments

`object`	An object of class Arena.
`newdiffmat`	A matrix giving the new gradient matrix of the specific substances in the environment.
`mediac`	A character vector giving the names of substances, which should be added to the environment (the default takes all possible substances).

Details

This function can be used to add gradients of specific substances in the environment. The default conditions in `changeSubs` assumes an equal concentration in every grid cell of the environment.

See Also

`Arena-class` and `changeSub`

Examples

```
data(Ec_core, envir = environment()) #get Escherichia coli core metabolic model
bac <- Bac(Ec_core,deathrate=0.05,
           minweight=0.05,growtype="exponential") #initialize a bacterium
arena <- Arena(n=20,m=20) #initialize the environment
arena <- addOrg(arena,bac,amount=10) #add 10 organisms
arena <- addSubs(arena,30) #add all substances with no concentrations.
gradient <- matrix(1:200,20,20)
arena <- changeDiff(arena,gradient,c("EX_glc(e)","EX_o2(e)","EX_pi(e)"))
# add substances glucose, oxygen and phosphate
```

changeFobj — *Function for changing the objective function of the model*

Description

The generic function changeFobj changes the objective function, which is used for the linear programming in optimizeLP.

Usage

```
changeFobj(object, new_fobj, model, alg = "fba")

## S4 method for signature 'Human'
changeFobj(object, new_fobj, model, alg = "fba")
```

Arguments

object	An object of class Human.
new_fobj	A character vector giving the reaction name of the new objective function.
model	The original model structure which is converted into a problem object used for the next optimization.
alg	A character vector giving the algorithm which should be used for the optimization (default is flux balance analysis).

Details

To avoid the bias to just one particular objective function, the objective can be changed dynamically in this function.

See Also

Human-class and optimizeLP

Examples

```
data(Ec_core, envir = environment()) #get Escherichia coli core metabolic model
human <- Human(Ec_core,deathrate=0.05,
          minweight=0.05,growtype="exponential") #initialize a bacterium
changeFobj(human,'EX_glc(e)',Ec_core)
```

changeOrg *Change organisms in the environment*

Description

The generic function `changeOrg` changes organisms in the environment.

Usage

```
changeOrg(object, neworgdat)

## S4 method for signature 'Arena'
changeOrg(object, neworgdat)
```

Arguments

`object`	An object of class Arena.
`neworgdat`	A data frame with new information about the accumulated growth, type, phenotype, x and y position for each individual in the environment.

Details

The argument `neworgdat` contains the same information as the `orgdat` slot of `Arena-class`. The `orgdat` slot of an `Arena` object can be used to create `neworgdat`.

See Also

`Arena-class` and `addOrg`

Examples

```
data(Ec_core, envir = environment()) #get Escherichia coli core metabolic model
bac <- Bac(Ec_core,deathrate=0.05,
           minweight=0.05,growtype="exponential") #initialize a bacterium
arena <- Arena(n=20,m=20) #initialize the environment
arena <- addOrg(arena,bac,amount=10) #add 10 organisms
neworgdat <- arena@orgdat #get the current orgdat
neworgdat <- neworgdat[-1,] #remove the first individual
arena <- changeOrg(arena,neworgdat)
```

changeSub *Change substances in the environment*

Description

The generic function changeSub changes specific substances in the environment.

Usage

```
changeSub(object, smax, mediac, unit = "mmol/cell")

## S4 method for signature 'Arena'
changeSub(object, smax, mediac, unit = "mmol/cell")
```

Arguments

object	An object of class Arena.
smax	A number or vector of numbers indicating the maximum substance concentration per grid cell.
mediac	A character vector giving the names of substances, which should be added to the environment (the default takes all possible substances).
unit	A character used as chemical unit to set the amount of the substances to be added (valid values are: mmol/cell, mmol/cm2, mmol/arena, mM)

Details

If nothing but `object` is given, then all possible substrates are initilized with a concentration of 0. Afterwards, changeSub can be used to modify the concentrations of specific substances.

See Also

Arena-class and addSubs

Examples

```
data(Ec_core, envir = environment()) #get Escherichia coli core metabolic model
bac <- Bac(Ec_core,deathrate=0.05,
           minweight=0.05,growtype="exponential") #initialize a bacterium
arena <- Arena(n=20,m=20) #initialize the environment
arena <- addOrg(arena,bac,amount=10) #add 10 organisms
arena <- addSubs(arena) #add all substances with no concentrations.
arena <- changeSub(arena,20,c("EX_glc(e)","EX_o2(e)","EX_pi(e)"))
#add substances glucose, oxygen and phosphate
```

checkCorr — *Function to show correlations of a simulated organism or substrate*

Description

The generic function checkCorr returns the correlation matrix of several objects.

Usage

```
checkCorr(object, corr = NULL, tocheck = list())

## S4 method for signature 'Eval'
checkCorr(object, corr = NULL, tocheck = list())
```

Arguments

object	An object of class Eval.
corr	A correlation matrix (getCorrM)
tocheck	A list with substrate, reactions or organism names whose correlations should be shown

Details

Returns correlation matrix which can be used for statistical analysis

See Also

Eval-class and getCorrM

Examples

```
data(Ec_core, envir = environment()) #get Escherichia coli core metabolic model
bac <- Bac(Ec_core,deathrate=0.05,
           minweight=0.05,growtype="exponential") #initialize a bacterium
arena <- Arena(n=20,m=20) #initialize the environment
arena <- addOrg(arena,bac,amount=10) #add 10 organisms
arena <- addSubs(arena,40) #add all possible substances
eval <- simEnv(arena,5)
checkCorr(eval, tocheck="o2")
```

checkPhen *Function for checking phenotypes in the environment*

Description

The generic function checkPhen checks and adds the phenotypes of organisms in the environment.

Usage

```
checkPhen(object, org, cutoff = 1e-06, fbasol)

## S4 method for signature 'Arena'
checkPhen(object, org, cutoff = 1e-06, fbasol)
```

Arguments

object	An object of class Arena.
org	An object of class Organism.
cutoff	A number giving the cutoff for values of the objective function and fluxes of exchange reactions.
fbasol	Problem object according to the constraints and then solved with optimizeProb.

Details

The phenotypes are defined by flux through exchange reactions, which indicate potential differential substrate usages. Uptake of substances are indicated by a negative and production of substances by a positive number.

Value

Returns a number indicating the number of the phenotype in the phenotype list.

See Also

Arena-class and getPhenotype

checkPhen_par *Function for checking phenotypes in the environment*

Description

The generic function checkPhen_par checks and adds the phenotypes of organisms in the environment.

Usage

```
checkPhen_par(object, org, cutoff = 1e-06, fbasol)

## S4 method for signature 'Arena'
checkPhen_par(object, org, cutoff = 1e-06, fbasol)
```

Arguments

object	An object of class Arena.
org	An object of class Organism.
cutoff	A number giving the cutoff for values of the objective function and fluxes of exchange reactions.
fbasol	Problem object according to the constraints and then solved with optimizeProb.

chemotaxis *Function for chemotaxis of bacteria to their prefered substrate*

Description

The generic function chemotaxis implements a bacterial movement in the Moore neighbourhood to the highest substrate concentration.

Usage

```
chemotaxis(object, population, j)

## S4 method for signature 'Bac'
chemotaxis(object, population, j)
```

Arguments

object	An object of class Bac.
population	An object of class Arena.
j	The number of the iteration of interest.

Details

Bacteria move to a position in the Moore neighbourhood which has the highest concentration of the prefered substrate, which is not occupied by other individuals. The prefered substance is given by slot chem in the Bac object. If there is no free space the individuals stays in the same position. If the concentration in the Moore neighbourhood has the same concentration in every position, then random movement is implemented.

See Also

Bac-class and emptyHood

Examples

```
data(Ec_core, envir = environment()) #get Escherichia coli core metabolic model
bac <- Bac(Ec_core,deathrate=0.05, chem = "EX_o2(e)",
           minweight=0.05,growtype="exponential") #initialize a bacterium
arena <- Arena(n=20,m=20) #initialize the environment
arena <- addOrg(arena,bac,amount=10) #add 10 organisms
arena <- addSubs(arena,40) #add all possible substances
chemotaxis(bac,arena,1)
```

---

constrain — *Function for constraining the models based on metabolite concentrations*

Description

The generic function constrain changes the constraints of the model representation of an organism.

Usage

```
constrain(object, reacts, lb, dryweight, time, scale, j)

## S4 method for signature 'Organism'
constrain(object, reacts, lb, dryweight, time, scale, j)
```

Arguments

object	An object of class Organisms.
reacts	A character vector giving the names of reactions which should be constrained.
lb	A numeric vector giving the constraint values of lower bounds (e.g. avaible metabolite concentrations
dryweight	A number giving the current dryweight of the organism.
time	A number giving the time intervals for each simulation step.
scale	A numeric defining the scaling (units for linear programming has to be in certain range)
j	debuging index to track cell

Details

The constraints are calculated according to the flux definition as mmol/(gDW*hr) with the parameters `dryweight` and `time`.

Value

Returns the lower bounds, which carry the constraints and names of relevant reactions.

See Also

Organism-class

Examples

```
data(Ec_core, envir = environment()) #get Escherichia coli core metabolic model
org <- Organism(Ec_core,deathrate=0.05,
           minweight=0.05,growtype="exponential") #initialize an organism
lobnds <- constrain(org,org@medium,org@lbnd[org@medium],1,1)
```

---

consume *Function to account for the consumption and production of substances*

Description

The generic function consume implements the consumption and production of substances based on the flux of exchange reactions of organisms

Usage

```
consume(object, sublb, cutoff = 1e-06, bacnum, fbasol)

## S4 method for signature 'Organism'
consume(object, sublb, cutoff = 1e-06, bacnum, fbasol)
```

Arguments

`object`	An object of class Organisms.
`sublb`	A vector containing the substance concentrations in the current position of the individual of interest.
`cutoff`	A number giving the cutoff value by which value of objective function is considered greater than 0.
`bacnum`	Integer indicating the number of bacteria individuals per gridcell
`fbasol`	Problem object according to the constraints and then solved with `optimizeProb`.

Details

The consumption is implemented by adding the flux of the exchange reactions to the current substance concentrations.

Value

Returns the updated vector containing the substance concentrations in the current position of the individual of interest.

See Also

Organism-class

Examples

```
NULL
```

`createGradient` *Change substance concentration patterns in the environment according to a gradient*

Description

The generic function `createGradient` changes specific substance concentration patterns in the environment.

Usage

```
createGradient(object, mediac, position, smax, steep, add = FALSE,
  unit = "mmol/cell")

## S4 method for signature 'Arena'
createGradient(object, mediac, position, smax, steep,
  add = FALSE, unit = "mmol/cell")
```

Arguments

`object`	An object of class Arena.
`mediac`	A character vector giving the names of substances, which should be added to the environment (the default takes all possible substances).
`position`	A character vector giving the position (top, bottom, right and left) of the gradient.
`smax`	A number giving the maximum concentration of the substance.
`steep`	A number between 0 and 1 giving the steepness of the gradient (concentration relative to the arena size).

add	A boolean variable defining whether the amount of substance should be summed or replaced
unit	A character used as chemical unit to set the amount of the substances to be added (valid values are: mmol/cell, mmol/cm2, mmol/arena, mM)

Details

This function can be used to add gradients of specific substances in the environment.

See Also

Arena-class and changeSub

Examples

```
data(Ec_core, envir = environment()) #get Escherichia coli core metabolic model
bac <- Bac(Ec_core,deathrate=0.05,
           minweight=0.05,growtype="exponential") #initialize a bacterium
arena <- Arena(n=20,m=20) #initialize the environment
arena <- addOrg(arena,bac,amount=10) #add 10 organisms
arena <- addSubs(arena,30) #add all substances with no concentrations.
arena <- createGradient(arena,smax=50,mediac=c("EX_glc(e)","EX_o2(e)","EX_pi(e)"),
             position='top',steep=0.5, add=FALSE)
```

---

dat2mat *Function for transforming the organism data frame to a presence/absence matrix of organisms*

Description

The generic function dat2mat simulates the event of mixing all substrates and organisms in the environment.

Usage

```
dat2mat(object)

## S4 method for signature 'Arena'
dat2mat(object)
```

Arguments

object	An object of class Arena.

Value

Returns the presence/absence matrix of organisms on the grid based on the orgdat slot of the Arena class.

See Also

Arena-class and getSublb

Examples

```
data(Ec_core, envir = environment()) #get Escherichia coli core metabolic model
bac <- Bac(Ec_core,deathrate=0.05,
           minweight=0.05,growtype="exponential") #initialize a bacterium
arena <- Arena(n=20,m=20) #initialize the environment
arena <- addOrg(arena,bac,amount=10) #add 10 organisms
occmat <- dat2mat(arena)
image(occmat)
```

---

diffuse — *Function for diffusion*

Description

The generic function `diffuse` computes the media distribution via diffusion

Usage

```
diffuse(object, lrw, sublb)

## S4 method for signature 'Arena'
diffuse(object, lrw, sublb)
```

Arguments

`object`	An object of class Arena.
`lrw`	A numeric value needed by solver to estimate array size (by default lwr is estimated in the simEnv() by the function estimate_lrw())
`sublb`	A matrix with the substrate concentration for every individual in the environment based on their x and y position.

---

diffusePDE — *Function for diffusion of the Substance matrix*

Description

The generic function `diffusePDE` implements the diffusion by the solving diffusion equation.

Usage

```
diffusePDE(object, init_mat, gridgeometry, lrw = NULL, tstep)

## S4 method for signature 'Substance'
diffusePDE(object, init_mat, gridgeometry, lrw = NULL,
  tstep)
```

Arguments

`object`	An object of class Substance.
`init_mat`	A matrix with values to be used by the diffusion.
`gridgeometry`	A list specifying the geometry of the Arena
`lrw`	A numeric value needed by solver to estimate array size (by default lwr is estimated in simEnv() by the function estimate_lrw())
`tstep`	A numeric value giving the time step of integration

Details

Partial differential equation is solved to model 2d diffusion process in the arena.

See Also

`Substance-class` and `diffuseR`

Examples

```
arena <- Arena(n=100, m=100, stir=FALSE, Lx=0.025, Ly=0.025)
sub <- Substance(n=100,m=100,smax=0,name='test', difspeed=0.1,
                gridgeometry=arena@gridgeometry) #initialize test substance
sub@diffmat[ceiling(100/2),ceiling(100/2)] <- 40
diffusePDE(sub, init_mat=as.matrix(sub@diffmat),
          gridgeometry=arena@gridgeometry, tstep=arena@tstep)
```

---

diffuseR — *Function for naive diffusion (neighbourhood) of the Substance matrix*

Description

The generic function `diffuseR` implements the diffusion in the Moore neighbourhood in R.

Usage

```
diffuseR(object)

## S4 method for signature 'Substance'
diffuseR(object)
```

Arguments

`object`	An object of class Substance.

Details

The diffusion is implemented by iterating through each cell in the grid and taking the cell with the lowest concentration in the Moore neighbourhood to update the concentration of both by their mean.

See Also

Substance-class and diffusePDE

Examples

```
arena <- Arena(n=100, m=100, stir=FALSE, Lx=0.025, Ly=0.025)
sub <- Substance(n=20,m=20,smax=40,name='test',difunc='r',
                gridgeometry=arena@gridgeometry) #initialize test substance
diffuseR(sub)
```

---

diffuse_par *Function for parallelzied diffusion*

Description

The generic function `diffuse_par` computes the media distribution via diffusion in parallel

Usage

```
diffuse_par(object, lrw, cluster_size, sublb)

## S4 method for signature 'Arena'
diffuse_par(object, lrw, cluster_size, sublb)
```

Arguments

`object`	An object of class Arena.
`lrw`	A numeric value needed by solver to estimate array size (by default lwr is estimated in the simEnv() by the function estimate_lrw())
`cluster_size`	Amount of cores to be used
`sublb`	A matrix with the substrate concentration for every individual in the environment based on their x and y position.

emptyHood *Function to check if the there is a free place in the Moore neighbourhood*

Description

The generic function emptyHood gives a free space which is present in the Moore neighbourhood of an individual of interest.

Usage

```
emptyHood(object, pos, n, m, x, y)

## S4 method for signature 'Organism'
emptyHood(object, pos, n, m, x, y)
```

Arguments

object	An object of class Organisms.
pos	A dataframe with all occupied x and y positions
n	A number giving the horizontal size of the environment.
m	A number giving the vertical size of the environment.
x	A number giving the x position of the individual of interest in its environment.
y	A number giving the y position of the individual of interest in its environment.

Value

Returns the free position in the Moore neighbourhood, which is not occupied by other individuals. If there is no free space NULL is returned.

See Also

Organism-class

Examples

```
NULL
```

Eval-class *Structure of the S4 class "Eval"*

Description

Structure of the S4 class `Eval` inheriting from class `Arena-class` for the analysis of simulations.

Slots

- `medlist` A list of compressed medium concentrations (only changes of concentrations are stored) per time step.
- `simlist` A list of the organism features per time step.
- `mfluxlist` A list of containing highly used metabolic reactions per time step.
- `shadowlist` A list of containing shadow prices per time step.
- `subchange` A vector of all substrates with numbers indicating the degree of change in the overall simulation.

Eval-constructor *Constructor of the S4 class* `Eval-class`

Description

Constructor of the S4 class `Eval-class`

Usage

```
Eval(arena)
```

Arguments

`arena`	An object of class Arena.

evalArena — *Function for plotting spatial and temporal change of populations and/or concentrations*

Description

The generic function evalArena plots heatmaps from the simulation steps in an Eval object.

Usage

```
evalArena(object, plot_items = "Population", phencol = F, retdata = F,
  time = (seq_along(object@simlist) - 1), show_legend = TRUE,
  legend_pos = "left")

## S4 method for signature 'Eval'
evalArena(object, plot_items = "Population", phencol = F,
  retdata = F, time = (seq_along(object@simlist) - 1), show_legend = TRUE,
  legend_pos = "left")
```

Arguments

object	An object of class Eval.
plot_items	A character vector giving the items, which should be plotted.
phencol	A boolean variable indicating if the phenotypes of the organisms in the environment should be integrated as different colors in the population plot.
retdata	A boolean variable indicating if the data used to generate the plots should be returned.
time	A numeric vector giving the simulation steps which should be plotted.
show_legend	A boolean variable indicating if a legend shuld be shown.
legend_pos	Position of the legend.

Details

If phencol is TRUE then different phenotypes of the same organism are visualized by varying colors, otherwise different organism types are represented by varying colors. The parameter retdata can be used to access the data used for the returned plots to create own custom plots.

Value

Returns several plots of the chosen plot items. Optional the data to generate the original plots can be returned.

See Also

Eval-class and Arena-class

Examples

```
data(Ec_core, envir = environment()) #get Escherichia coli core metabolic model
bac <- Bac(Ec_core,deathrate=0.05,
           minweight=0.05,growtype="exponential") #initialize a bacterium
arena <- Arena(n=20,m=20) #initialize the environment
arena <- addOrg(arena,bac,amount=10) #add 10 organisms
arena <- addSubs(arena,40) #add all possible substances
eval <- simEnv(arena,5)
evalArena(eval)
## Not run:
## if animation package is installed a movie of the simulation can be stored:
library(animation)
saveVideo({evalArena(eval)},video.name="Ecoli_sim.mp4")

## End(Not run)
```

`extractMed` *Function for re-constructing a medium concentrations from simulations*

Description

The generic function `extractMed` re-constructs a list of vectors of medium concentrations from a simulation step in an `Eval` object.

Usage

```
extractMed(object, time = length(object@medlist), mediac = object@mediac)

## S4 method for signature 'Eval'
extractMed(object, time = length(object@medlist),
  mediac = object@mediac)
```

Arguments

`object`	An object of class Eval.
`time`	A number giving the simulation step of interest.
`mediac`	A character vector giving the names of substances, which should be added to the environment (the default takes all possible substances).

Details

Medium concentrations in slot `medlist` of an object of class `Eval` store only the changes of concentrations in the simulation process. The function `extractMed` reconstructs the original and uncompressed version of medium concentrations.

Value

Returns a list containing concentration vectors of all medium substances.

See Also

Eval-class and Arena-class

Examples

```
data(Ec_core, envir = environment()) #get Escherichia coli core metabolic model
bac <- Bac(Ec_core,deathrate=0.05,
           minweight=0.05,growtype="exponential") #initialize a bacterium
arena <- Arena(n=20,m=20) #initialize the environment
arena <- addOrg(arena,bac,amount=10) #add 10 organisms
arena <- addSubs(arena,40) #add all possible substances
eval <- simEnv(arena,5)
med5 <- extractMed(eval,5)
```

---

findFeeding — *Function for investigation of feeding between phenotypes*

Description

The generic function findFeeding

Usage

```
findFeeding(object, dict = NULL, tcut = 5, scut = NULL, org_dict = NULL,
  legendpos = "topleft", lwd = 1)

## S4 method for signature 'Eval'
findFeeding(object, dict = NULL, tcut = 5, scut = NULL,
  org_dict = NULL, legendpos = "topleft", lwd = 1)
```

Arguments

object	An object of class Eval.
dict	List defining new substance names. List entries are intepreted as old names and the list names as the new ones.
tcut	Integer giving the minimal mutual occurence ot be considered (dismiss very seldom feedings)
scut	substance names which should be ignored
org_dict	A named list/vector with names that should replace (eg. unreadable) IDs
legendpos	A character variable declaring the position of the legend
lwd	Line thickness scale in graph

Value

Graph (igraph)

---

findFeeding2 *Function for investigation of feeding between phenotypes*

Description

The generic function `findFeeding2`

Usage

```
findFeeding2(object, time, mets, rm_own = T, ind_threshold = 0,
  collapse = F)

## S4 method for signature 'Eval'
findFeeding2(object, time, mets, rm_own = T,
  ind_threshold = 0, collapse = F)
```

Arguments

`object`	An object of class Eval.
`time`	A numeric vector giving the simulation steps which should be plotted.
`mets`	Character vector of substance names which should be considered
`rm_own`	A boolean flag indicating if interactions within same species should be plotted
`ind_threshold`	A number indicating the threshold of individuals to be considered as producers/consumers
`collapse`	A boolean flag indicating if all phenotypes for every species should be collapsed to either producers or consumers

Value

Graph (igraph)

findFeeding3 — *Function for investigation of feeding between phenotypes*

Description

The generic function findFeeding3

Usage

```
findFeeding3(object, time, mets)

## S4 method for signature 'Eval'
findFeeding3(object, time, mets)
```

Arguments

object	An object of class Eval.
time	A numeric vector giving the simulation steps which should be plotted.
mets	Character vector of substance names which should be considered

Value

Graph (igraph)

findInArena — *Function for searching a keyword in arena organisms and media*

Description

The generic function findInArena tries to find information (e.g. full names) about a specific keyword

Usage

```
findInArena(object, pattern, search_rea = TRUE, search_sub = TRUE)

## S4 method for signature 'Arena'
findInArena(object, pattern, search_rea = TRUE,
  search_sub = TRUE)
```

Arguments

object	An object of class Arena.
pattern	A pattern for searching
search_rea	Only search for reactions
search_sub	Only search for substances

Examples

```
data(Ec_core)
bac <- Bac(Ec_core)
arena <- Arena(n=20,m=20)
arena <- addOrg(arena,bac,amount=10)
findInArena(arena, "acetate")
```

`flushSubs` *Remove all substances in the environment*

Description

The generic function `flushSubs` removes specific substances in the environment.

Usage

```
flushSubs(object)

## S4 method for signature 'Arena'
flushSubs(object)
```

Arguments

`object`	An object of class Arena.

See Also

`Arena-class` and `addSubs`

Examples

```
data(Ec_core, envir = environment()) #get Escherichia coli core metabolic model
bac <- Bac(Ec_core,deathrate=0.05,
           minweight=0.05,growtype="exponential") #initialize a bacterium
arena <- Arena(n=20,m=20) #initialize the environment
arena <- addOrg(arena,bac,amount=10) #add 10 organisms
arena <- addSubs(arena, smax=40) #add all substances with no concentrations.
arena <- changeSub(arena,20,c("EX_glc(e)","EX_o2(e)","EX_pi(e)"))
#add substances glucose, oxygen and phosphate
arena <- flushSubs(arena) #remove all created substance concentrations
```

`getArena` *Function for re-constructing an Arena object from a simulation step*

Description

The generic function `getArena` re-constructs an `Arena` object from a simulation step within an `Eval` object.

Usage

```
getArena(object, time = (length(object@medlist) - 1))

## S4 method for signature 'Eval'
getArena(object, time = (length(object@medlist) - 1))
```

Arguments

`object`	An object of class Eval.
`time`	A number giving the simulation step of interest.

Details

The function `addEval` can be used to manipulate an `Arena` object from a simulation step to modify the subsequent simulation steps.

Value

Returns an object of class `Arena` containing the organisms and substance conditions in simulation step `time`.

See Also

`Eval-class` and `Arena-class`

Examples

```
data(Ec_core, envir = environment()) #get Escherichia coli core metabolic model
bac <- Bac(Ec_core,deathrate=0.05,
           minweight=0.05,growtype="exponential") #initialize a bacterium
arena <- Arena(n=20,m=20) #initialize the environment
arena <- addOrg(arena,bac,amount=10) #add 10 organisms
arena <- addSubs(arena,40) #add all possible substances
eval <- simEnv(arena,5)
arena5 <- getArena(eval,5)
```

getCorrM — *Function to compute and return correlation matrix*

Description

The generic function `getCorrM` returns the correlation matrix of several objects.

Usage

```
getCorrM(object, reactions = TRUE, bacs = TRUE, substrates = TRUE)

## S4 method for signature 'Eval'
getCorrM(object, reactions = TRUE, bacs = TRUE,
  substrates = TRUE)
```

Arguments

`object`	An object of class Eval.
`reactions`	A boolean indicating whether reactions should be included in correlation matrix
`bacs`	A boolean indicating whether bacteria should be included in correlation matrix
`substrates`	A boolean indicating whether substrates should be included in correlation matrix

Details

Returns correlation matrix which can be used for statistical analysis

Value

correlation matrix

See Also

`Eval-class`

Examples

```
data(Ec_core, envir = environment()) #get Escherichia coli core metabolic model
bac <- Bac(Ec_core,deathrate=0.05,
           minweight=0.05,growtype="exponential") #initialize a bacterium
arena <- Arena(n=20,m=20) #initialize the environment
arena <- addOrg(arena,bac,amount=10) #add 10 organisms
arena <- addSubs(arena,40) #add all possible substances
eval <- simEnv(arena,5)
getCorrM(eval)
```

getPhenoMat *Function for getting a matrix of phenotypes from the dataset*

Description

The generic function getPhenoMat reconstructs a matrix with the usage of exchange reactions of the different organisms in the environment.

Usage

```
getPhenoMat(object, time = "total", sparse = F)

## S4 method for signature 'Eval'
getPhenoMat(object, time = "total", sparse = F)
```

Arguments

object	An object of class Eval.
time	An integer indicating the time step to be used (default value is character "total")
sparse	A boolean indicating whether zero entries should be removed from return matrix

Details

The phenotypes are defined by flux through exchange reactions, which indicate potential differential substrate usages.

Value

Returns a matrix with different phenotypes of the organism as rows and all possible exchange reactions as columns. A value of 1 means secretion, 2 means uptake and 0 means no usage of the substance of interest.

See Also

Eval-class and getPhenotype

Examples

```
data(Ec_core, envir = environment()) #get Escherichia coli core metabolic model
bac <- Bac(Ec_core,deathrate=0.05,
           minweight=0.05,growtype="exponential") #initialize a bacterium
arena <- Arena(n=20,m=20) #initialize the environment
arena <- addOrg(arena,bac,amount=10) #add 10 organisms
arena <- addSubs(arena,40) #add all possible substances
eval <- simEnv(arena,5)
phenmat <- getPhenoMat(eval)
```

getPhenotype *Function to extract the phenotype of an organism object*

Description

The generic function getPhenotype implements an identification of organism phenotypes.

Usage

```
getPhenotype(object, cutoff = 1e-06, fbasol, par = FALSE)

## S4 method for signature 'Organism'
getPhenotype(object, cutoff = 1e-06, fbasol,
  par = FALSE)
```

Arguments

object	An object of class Organisms.
cutoff	A number giving the cutoff value by which value of objective function is considered greater than 0.
fbasol	Problem object according to the constraints and then solved with optimizeProb.
par	A boolean indicating if running in parallel mode.

Details

The phenotypes are defined by flux through exchange reactions, which indicate potential differential substrate usages. Uptake of substances is indicated by a negative and production of substances by a positive number.

Value

Returns the phenotype of the organisms where the uptake of substances is indicated by a negative and production of substances by a positive number

See Also

Organism-class, checkPhen and minePheno

getSubHist — *Function to get timeline of a substance*

Description

The generic function `getSubHist` returns list with amount of substance for each timestep

Usage

```
getSubHist(object, sub)

## S4 method for signature 'Eval'
getSubHist(object, sub)
```

Arguments

`object`	An object of class Eval.
`sub`	Name of a substance.

getSublb — *Function for calculated the substrate concentration for every organism*

Description

The generic function `getSublb` calculates the substrate concentration for every individual in the environment based on their x and y position.

Usage

```
getSublb(object)

## S4 method for signature 'Arena'
getSublb(object)
```

Arguments

`object`	An object of class Arena.

Value

Returns the substrate concentration for every individual in the environment with substrates as well as x and y positions as columns and rows for each organism.

See Also

Arena-class

Examples

```
data(Ec_core, envir = environment()) #get Escherichia coli core metabolic model
bac <- Bac(Ec_core,deathrate=0.05,
           minweight=0.05,growtype="exponential") #initialize a bacterium
arena <- Arena(n=20,m=20) #initialize the environment
arena <- addOrg(arena,bac,amount=10) #add 10 organisms
arena <- addSubs(arena,40) #add all possible substances
sublb <- getSublb(arena)
```

---

getVarSubs *Function to get varying substances*

Description

The generic function getVarSubs returns ordered list of substances that showed variance during simulation

Usage

```
getVarSubs(object, show_products = TRUE, show_substrates = TRUE,
  cutoff = 1e-06, size = NULL)

## S4 method for signature 'Eval'
getVarSubs(object, show_products = FALSE,
  show_substrates = FALSE, cutoff = 1e-06, size = NULL)
```

Arguments

object	An object of class Eval.
show_products	A boolean indicating if only products should be shown
show_substrates	A boolean indicating if only substrates should be shown
cutoff	Value used to define numeric accuracy while interpreting optimization results
size	Maximal number of returned substances (default: show all)

growExp *Function for letting organisms grow exponentially*

Description

The generic function `growExp` implements a growth model of organisms in their environment.

Usage

```
growExp(object, growth, fbasol)

## S4 method for signature 'Organism'
growExp(object, growth, fbasol)
```

Arguments

`object`	An object of class Organisms.
`growth`	A number indicating the current biomass, which has to be updated.
`fbasol`	Problem object according to the constraints and then solved with `optimizeProb`.

Details

Exponential growth of organisms is implemented by adding the calculated growthrate multiplied with the current growth calculated by `optimizeLP` plus to the already present growth value

Value

Returns the updated biomass of the organisms of interest.

See Also

`Organism-class` and `optimizeLP`

growLin *Function for letting organisms grow linearly*

Description

The generic function `growLin` implements a growth model of organisms in their environment.

Usage

```
growLin(object, growth, fbasol)

## S4 method for signature 'Organism'
growLin(object, growth, fbasol)
```

Arguments

`object`	An object of class Organisms.
`growth`	A number indicating the current biomass, which has to be updated.
`fbasol`	Problem object according to the constraints and then solved with `optimizeProb`.

Details

Linear growth of organisms is implemented by adding the calculated growthrate by `optimizeLP` to the already present growth value.

Value

Returns the updated biomass of the organisms of interest.

See Also

`Organism-class` and `optimizeLP`

`growth` *Function implementing a growth model of a bacterium*

Description

The generic function `growth` implements different growth models for an object of class Bac.

Usage

```
growth(object, population, j, occupyM, fbasol)

## S4 method for signature 'Bac'
growth(object, population, j, occupyM, fbasol)
```

Arguments

`object`	An object of class Bac.
`population`	An object of class Arena.
`j`	The index of the organism of interest in orgdat.
`occupyM`	A matrix indicating grid cells that are obstacles
`fbasol`	Problem object according to the constraints and then solved with `optimizeProb`.

Details

Linear growth of organisms is implemented by adding the calculated growthrate by `optimizeLP` to the already present growth value. Exponential growth of organisms is implemented by adding the calculated growthrate multiplied with the current growth calculated by `optimizeLP` plus to the already present growth value

Value

Boolean variable of the jth individual indicating if individual died.

See Also

Bac-class, growLin and growExp

---

growth_par *Function implementing a growth model of a bacterium*

Description

The generic function growth_par implements different growth models for an object of class Bac.

Usage

```
growth_par(object, population, j, fbasol)

## S4 method for signature 'Bac'
growth_par(object, population, j, fbasol)
```

Arguments

object	An object of class Bac.
population	An object of class Arena.
j	The index of the organism of interest in orgdat.
fbasol	Problem object according to the constraints and then solved with optimizeProb.

Value

A list

Human-class *Structure of the S4 class "Human"*

Description

Structure of the S4 class Human inheriting from class Organism-class representing human cells.

Slots

objective A character vector representing the current reaction which should be used as an objective function for the flux balance analysis.

Human-constructor — *Constructor of the S4 class* Human-class

Description

Constructor of the S4 class Human-class

Usage

```
Human(model, objective = model@react_id[which(model@obj_coef == 1)],
  speed = 0, ...)
```

Arguments

model	model
objective	A character vector representing the current reaction which should be used as an objective function for the flux balance analysis.
speed	A integer vector representing the speed by which bacterium is moving (given by cell per iteration).
...	Arguments of Organism-class

Value

Object of class Human-class

lsd — *Computer standard deviation lower bound*

Description

Helper function to get lower error bounds in plotting

Usage

```
lsd(y)
```

Arguments

y	Vector with numbers

`lysis` *Lysis function of organismal cells by adding biomass_compounds to the medium*

Description

The generic function `lysis` implements cell lysis by the stochiometric concentration of the biomass compounds of organisms to the concentration of substances in the environment

Usage

```
lysis(object, sublb, factor = object@minweight)

## S4 method for signature 'Organism'
lysis(object, sublb, factor = object@minweight)
```

Arguments

`object`	An object of class Organisms.
`sublb`	A vector containing the substance concentrations in the current position of the individual of interest.
`factor`	A number given the factor with which the biomass compound concentrations are multiplied to achieve the final concentration which is added to the environment

Details

Lysis is implemented by taking the intersect between biomass compounds and the substances in the environment and adding the normalized stochiometric concentrations of the biomass compounds to the medium.

Value

Returns the updated vector containing the substance concentrations in the current position of the dead individual of interest.

See Also

`Organism-class` and `optimizeLP`

Examples

```
NULL
```

minePheno — *Function for mining/analyzing phenotypes which occured on the arena*

Description

The generic function minePheno mines the similarity and differences of phenotypes reconstructed by getPhenoMat for each simulation step in an Eval object.

Usage

```
minePheno(object, plot_type = "pca", legend = F, time = "total")

## S4 method for signature 'Eval'
minePheno(object, plot_type = "pca", legend = F,
  time = "total")
```

Arguments

object	An object of class Eval.
plot_type	A character vector giving the plot which should be returned (either "pca" for a principle coordinate analysis or "hclust" for hierarchical clustering).
legend	Boolean variable indicating if legend should be plotted
time	An integer indicating the time step to be used (default value is character "total")

Details

The phenotypes are defined by flux through exchange reactions, which indicate potential differential substrate usages.

Value

Returns a plot for each simulation step representing the similarity of phenotypes of organisms within the environment.

See Also

Eval-class and getPhenoMat

Examples

```
data(Ec_core, envir = environment()) #get Escherichia coli core metabolic model
bac <- Bac(Ec_core,deathrate=0.05,
           minweight=0.05,growtype="exponential") #initialize a bacterium
arena <- Arena(n=20,m=20) #initialize the environment
arena <- addOrg(arena,bac,amount=10) #add 10 organisms
arena <- addSubs(arena,40) #add all possible substances
eval <- simEnv(arena,5)
minePheno(eval)
```

move *Function for random movement of organisms*

Description

The generic function move implements a random movement in the Moore neighbourhood of an individual.

Usage

```
move(object, pos, n, m, j, occupyM)

## S4 method for signature 'Organism'
move(object, pos, n, m, j, occupyM)
```

Arguments

object	An object of class Organism.
pos	A dataframe with all occupied x and y positions
n	A number giving the horizontal size of the environment.
m	A number giving the vertical size of the environment.
j	The number of the iteration of interest.
occupyM	A matrix indicating grid cells that are obstacles

Details

Organisms move in a random position the Moore neighbourhood, which is not occupied by other individuals. If there is no free space the individuals stays in the same position.

See Also

Organism-class, emptyHood

Examples

```
data(Ec_core, envir = environment()) #get Escherichia coli core metabolic model
bac <- Bac(Ec_core,deathrate=0.05,
           minweight=0.05,growtype="exponential") #initialize a bacterium
arena <- Arena(n=20,m=20) #initialize the environment
arena <- addOrg(arena,bac,amount=10) #add 10 organisms
arena <- addSubs(arena,40) #add all possible substances
move(bac,n=20,m=20,j=1,pos=arena@orgdat[,c('x','y')], occupyM=arena@occupyM)
```

NemptyHood — *Function to check if the there is a free place in the Moore neighbourhood*

Description

The generic function NemptyHood gives a free space which is present in the Moore neighbourhood of an individual of interest.

Usage

```
NemptyHood(object, pos, n, m, x, y)

## S4 method for signature 'Organism'
NemptyHood(object, pos, n, m, x, y)
```

Arguments

object	An object of class Organisms.
pos	A dataframe with all occupied x and y positions
n	A number giving the horizontal size of the environment.
m	A number giving the vertical size of the environment.
x	A number giving the x position of the individual of interest in its environment.
y	A number giving the y position of the individual of interest in its environment.

Value

Returns the free position in the Moore neighbourhood, which is not occupied by other individuals. If there is no free space NULL is returned.

See Also

Organism-class

Examples

```
NULL
```

openArena *Start simulation*

Description

The function openArena can be used to start a default simulation.

Usage

```
openArena()
```

Value

Returns an object of class Eval which can be used for subsequent analysis steps.

Examples

```
sim <- openArena()
evalArena(sim, time=7, phencol = TRUE,
          plot_items=c("Population", "EX_o2(e)", "EX_for(e)",
          "EX_glc(e)", "EX_for(e)"))
```

optimizeLP *Function for computing the linear programming according to the model structure*

Description

The generic function optimizeLP implements a linear programming based on the problem structure and refined constraints.

Usage

```
optimizeLP(object, lpob = object@lpobj, lb = object@lbnd,
  ub = object@ubnd, cutoff = 1e-06, j, sec_obj = "none")

## S4 method for signature 'Organism'
optimizeLP(object, lpob = object@lpobj,
  lb = object@lbnd, ub = object@ubnd, cutoff = 1e-06, j,
  sec_obj = "none")
```

Arguments

`object`	An object of class Organisms.
`lpob`	A linear programing object encoding the problem to solve.
`lb`	A numeric vector giving the constraint values of lower bounds.
`ub`	A numeric vector giving the constraint values of upper bounds.
`cutoff`	value used to define numeric accuracy while interpreting optimization results
`j`	debuging index to track cell
`sec_obj`	character giving the secondary objective for a bi-level LP if wanted.

Value

Modified problem object according to the constraints and then solved with `optimizeProb`.

See Also

`Organism-class`, `optimizeProb` and `sysBiolAlg`

Examples

```
data(Ec_core, envir = environment()) #get Escherichia coli core metabolic model
org <- Organism(Ec_core,deathrate=0.05,
           minweight=0.05,growtype="exponential") #initialize a organism
org@fbasol <- optimizeLP(org)
```

`Organism-class` *Structure of the S4 class "Organism"*

Description

Structure of the S4 class `Organism` representing the organisms present in the environment.

Slots

- `lbnd` A numeric vector containing the lower bounds of the model structure.
- `ubnd` A numeric vector containing the upper bounds of the model structure.
- `type` A character vector containing the description of the organism.
- `medium` A character vector containing all exchange reactions of the organism.
- `lpobj` A sybil optimization object containing the linear programing problem.
- `fbasol` A list with the solutions of the flux balance analysis.
- `lyse` A boolean variable indicating if the organism should lyse after death.
- `feat` A list containing conditional features for the object (contains at the momement only biomass components for lysis).

`deathrate` A numeric value giving the factor by which the growth should be reduced in every iteration (unit: fg)

`minweight` A numeric value giving the growth limit at which the organism dies.

`growtype` A character vector giving the functional type for growth (linear or exponential).

`kinetics` A List containing Km and v_max values for each reactions.

`speed` A integer vector representing the speed by which bacterium is moving (given by cell per iteration).

`cellarea` A numeric value indicating the surface that one organism occupies (unit: mu cm^2)

`maxweight` A numeric value giving the maximal dry weight of single organism (unit: fg)

`cellweight_mean` A numeric giving the mean of starting biomass

`cellweight_sd` A numeric giving the standard derivation of starting biomass

`model` Object of class sybil::modelorg containging the genome sclae metabolic model

---

`Organism-constructor` *Constructor of the S4 class* `Organism`

Description

The constructor to get a new object of class `Organism`

Usage

```
Organism(model, algo = "fba", ex = "EX_", ex_comp = NA,
  csuffix = "\\[c\\]", esuffix = "\\[e\\]", lyse = F,
  feat = list(), typename = NA, setExInf = TRUE, ...)
```

Arguments

`model`	Object of class sybil::modelorg containging the genome sclae metabolic model
`algo`	A single character string giving the name of the algorithm to use. See SYBIL_SETTINGS
`ex`	Identifier for exchange reactions
`ex_comp`	ex_comp
`csuffix`	csuffix
`esuffix`	esuffix
`lyse`	A boolean variable indicating if the organism should lyse after death.
`feat`	A list containing conditional features for the object (contains at the momement only biomass components for lysis).
`typename`	A string defining the name (set to model name in default case)
`setExInf`	Enable if all lower bounds of exchange reaction which are set to zero (i.e. no uptake possible!) should be set to -infitity
`...`	Arguments of `Organism-class`

Value

Object of class Organism

plotAbundance *Plot abundances of species*

Description

The function plotAbundance takes a list of simulations and return a boxplot with species abundances

Usage

```
plotAbundance(simlist, time = c(NULL, NULL), col = colpal3,
  return_dat = F, use_biomass = F)
```

Arguments

simlist	A list of simulations (eval objects).
time	A vector with start and end time to be considered (default: total time)
col	Vector with color that should be used
return_dat	Should plain text mean abundances be returned? (default false)
use_biomass	If enabled then biomass is used instead of cell number

plotCurves *Function for plotting the overall change as curves*

Description

The generic function plotCurves plots the growth curves and concentration changes of substances from simulation steps in an Eval object.

Usage

```
plotCurves(object, medplot = object@mediac, retdata = F, remove = F,
  legend = F)

## S4 method for signature 'Eval'
plotCurves(object, medplot = object@mediac, retdata = F,
  remove = F, legend = F)
```

Arguments

`object`	An object of class Eval.
`medplot`	A character vector giving the name of substances which should be plotted.
`retdata`	A boolean variable indicating if the data used to generate the plots should be returned.
`remove`	A boolean variable indicating if substances, which don't change in their concentration should be removed from the plot.
`legend`	Boolean variable indicating if legend should be plotted

Details

The parameter `retdata` can be used to access the data used for the returned plots to create own custom plots.

Value

Returns two graphs in one plot: the growth curves and the curves of concentration changes. Optional the data to generate the original plots can be returned.

See Also

`Eval-class` and `Arena-class`

Examples

```
data(Ec_core, envir = environment()) #get Escherichia coli core metabolic model
bac <- Bac(Ec_core,deathrate=0.05,
           minweight=0.05,growtype="exponential") #initialize a bacterium
arena <- Arena(n=20,m=20) #initialize the environment
arena <- addOrg(arena,bac,amount=10) #add 10 organisms
arena <- addSubs(arena,40) #add all possible substances
eval <- simEnv(arena,5)
plotCurves(eval)
```

`plotCurves2` *Function for plotting the overall change as curves with maximally distinct colors*

Description

The generic function `plotCurves2` plots the growth curves and concentration changes of the most changing substances from simulation steps in an `Eval` object using maximally distinct colors.

Usage

```
plotCurves2(object, legendpos = "topleft", ignore = c("EX_h(e)", "EX_pi(e)",
  "EX_h2o(e)"), num = 10, phencol = FALSE, biomcol = FALSE, dict = NULL,
  subs = list(), growthCurve = TRUE, subCurve = TRUE)

## S4 method for signature 'Eval'
plotCurves2(object, legendpos = "topright",
  ignore = c("EX_h(e)", "EX_pi(e)", "EX_h2o(e)"), num = 10,
  phencol = FALSE, biomcol = FALSE, dict = NULL, subs = list(),
  growthCurve = TRUE, subCurve = TRUE)
```

Arguments

`object`	An object of class Eval.
`legendpos`	A character variable declaring the position of the legend
`ignore`	A list of character variables with substance names that sould be omitted in the plot
`num`	An integer defining the number of substrates to be plot
`phencol`	Boolean variable indicating whether phenotypes should be higlighted
`biomcol`	A boolean indicating if biomass should be included in gowth curve
`dict`	List defining new substance names. List entries are intepreted as old names and the list names as the new ones.
`subs`	List of substance names. If empty, substances with highest variance will be used.
`growthCurve`	True if growth curve should be shown (default TRUE)
`subCurve`	True if substance curve should be shown (default TRUE)

Details

The parameter `retdata` can be used to access the data used for the returned plots to create own custom plots.

Value

Returns two graphs in one plot: the growth curves and the curves of concentration changes

See Also

`Eval-class` and `Arena-class`

Examples

```
data(Ec_core, envir = environment()) #get Escherichia coli core metabolic model
bac <- Bac(Ec_core,deathrate=0.05,
           minweight=0.05,growtype="exponential") #initialize a bacterium
arena <- Arena(n=20,m=20) #initialize the environment
arena <- addOrg(arena,bac,amount=10) #add 10 organisms
arena <- addSubs(arena,40) #add all possible substances
```

```
eval <- simEnv(arena,5)
plotCurves2(eval)
```

---

plotFluxVar *Plot population flux variations*

Description

The function `plotFluxVar` takes a list of simulations and metabolites, returning a plot with metabolite fluxes for each species

Usage

```
plotFluxVar(simlist, metsel)
```

Arguments

`simlist`	A list of simulations (eval objects).
`metsel`	A vector with the name of exchange reactions of interest

---

plotGrowthCurve *Plot growth curve for several simulations*

Description

The function `plotGrowthCurve` takes a list of simulations and plots the time course of species with standard deviation.

Usage

```
plotGrowthCurve(simlist, bcol = colpal3, time = c(NULL, NULL))
```

Arguments

`simlist`	A list of simulations (eval objects).
`bcol`	Vector with color that should be used
`time`	Vector with two entries defining start and end time

plotInterNum — *Plot number of variation in number of interactions for several simulations*

Description

The function plotInterNum takes a list of simulations and plots the time course of the number of metabolic interactions with standard deviation.

Usage

```
plotInterNum(simlist, title = "Variation in number of interactions",
  size = 1)
```

Arguments

simlist	A list of simulations (eval objects).
title	Title of the plot
size	A scaling factor for plot text and line size

plotPhenCurve — *Plot growth curve for several simulations*

Description

The function plotPhenCurve takes a list of simulations and plots the time course of species with standard deviation.

Usage

```
plotPhenCurve(simlist, subs, phens = NULL, time = c(NULL, NULL),
  ret_phengroups = FALSE, cluster = TRUE, col = colpal3)
```

Arguments

simlist	A list of simulations (eval objects).
subs	A vector of substance names that are used for phenotype clustering.
phens	If phencurve is given then phens specifies the phenotypes which sould be plotted again.
time	Vector with two entries defining start and end time
ret_phengroups	True if clustered phenotype groups should be returned.
cluster	True phenotypes should be clustered/condensed.
col	Vector with color that should be used

plotPhenNum — *Plot number of phenotypes curve for several simulations*

Description

The function plotPhenNum takes a list of simulations and plots the time course of the number of phenotypes with standard deviation.

Usage

```
plotPhenNum(simlist, title = "Phenotype number variation", size = 1)
```

Arguments

simlist	A list of simulations (eval objects).
title	Title of the plot
size	A scaling factor for plot text and line size

plotShadowCost — *Function to plot substance shadow costs for a specie*

Description

The generic function plotShadowCost plots substances have the highest impact on further growth (shadow cost < 0)

Usage

```
plotShadowCost(object, spec_nr = 1, sub_nr = 10, cutoff = -1)

## S4 method for signature 'Eval'
plotShadowCost(object, spec_nr = 1, sub_nr = 10,
  cutoff = -1)
```

Arguments

object	An object of class Eval.
spec_nr	Number of the specie
sub_nr	Maximal number of substances to be show
cutoff	Shadow costs should be smaller than cutoff

Details

Returns ggplot objects

plotSpecActivity *Function to plot substance usage for every species*

Description

The generic function `plotSpecActivity` displays the input/output substances with the highest variance (could also be defiened manually) for all species

Usage

```
plotSpecActivity(simlist, subs = list(), var_nr = 10, spec_list = NULL,
  ret_data = FALSE)
```

Arguments

`simlist`	An object of class Eval or a list with objects of class Eval.
`subs`	List of substance names
`var_nr`	Number of most varying substances to be used (if subs is not specified)
`spec_list`	List of species names to be considered (default all)
`ret_data`	Set true if data should be returned

Details

Returns ggplot objects

---

plotSubCurve *Plot substance curve for several simulations*

Description

The function `plotSubCurve` takes a list of simulations and plots the time course of substances with standard deviation.

Usage

```
plotSubCurve(simlist, mediac = NULL, time = c(NULL, NULL), scol = NULL,
  unit = "mmol", ret_data = FALSE, num_var = 10)
```

Arguments

simlist	A list of simulations (eval objects).
mediac	A vector of substances (if not specified most varying substances will be taken.)
time	Vector with two entries defining start and end time.
scol	Vector with colors that should be used.
unit	Unit for the substances which should be used for plotting (default: mmol)
ret_data	Set true if data should be returned
num_var	Number of varying substances to be shown (if mediac is not specified)

Value

list of three ggplot object for further formating

---

plotSubUsage *Function to plot usage of substances species wise*

Description

The generic function `plotSubUsage` displays for given substances the quantities of absorption and production for each species

Usage

```
plotSubUsage(simlist, subs = list(), cutoff = 0.01, ret_data = FALSE)
```

Arguments

simlist	An object of class Eval or a list with objects of class Eval.
subs	List of substance names
cutoff	Total values below cutoff will be dismissed
ret_data	Set true if data should be returned

Details

Returns ggplot objects

plotSubVar — *Plot substance variations*

Description

The function `plotSubVar` takes a list of simulations and return a barplot with most varying substances

Usage

```
plotSubVar(simlist, metsel)
```

Arguments

`simlist`	A list of simulations (eval objects).
`metsel`	A vector with the name of exchange reactions of interest

plotTotFlux — *Function for plotting the overall change in reaction activity*

Description

The generic function `plotTotFlux` plots the time course of reactions with high variation in activity for an `Eval` object.

Usage

```
plotTotFlux(object, legendpos = "topright", num = 20)

## S4 method for signature 'Eval'
plotTotFlux(object, legendpos = "topright", num = 20)
```

Arguments

`object`	An object of class Eval.
`legendpos`	A character variable declaring the position of the legend
`num`	An integer defining the number of substrates to be plot

Examples

```
data(Ec_core, envir = environment()) #get Escherichia coli core metabolic model
bac <- Bac(Ec_core,deathrate=0.05,
           minweight=0.05,growtype="exponential") #initialize a bacterium
arena <- Arena(n=20,m=20) #initialize the environment
arena <- addOrg(arena,bac,amount=10) #add 10 organisms
arena <- addSubs(arena,40) #add all possible substances
eval <- simEnv(arena,5)
plotTotFlux(eval)
```

redEval — *Function for reducing the size of an Eval object by collapsing the medium concentrations*

Description

The generic function `redEval` reduces the object size of an `Eval` object.

Usage

```
redEval(object, time = "all")

## S4 method for signature 'Eval'
redEval(object, time = 1:length(object@medlist))
```

Arguments

`object`	An object of class Eval.
`time`	A number giving the simulation step of interest.

Details

The function `redEval` can be used to reduce the size of an `Eval` object from a simulation step.

Value

Returns an object of class `Arena` containing the organisms and substance conditions in simulation step `time`.

See Also

`Eval-class` and `Arena-class`

Examples

```
data(Ec_core, envir = environment()) #get Escherichia coli core metabolic model
bac <- Bac(Ec_core,deathrate=0.05,
          minweight=0.05,growtype="exponential") #initialize a bacterium
arena <- Arena(n=20,m=20) #initialize the environment
arena <- addOrg(arena,bac,amount=10) #add 10 organisms
arena <- addSubs(arena,40) #add all possible substances
eval <- simEnv(arena,5)
eval_reduce <- redEval(eval,5)
```

`reset_screen` *Reset plotting screen*

Description

The function `reset_screen` set plotting window to default

Usage

```
reset_screen()
```

`rmSubs` *Remove substances*

Description

The generic function `rmSubs` removes all amounts of substances available in the arena for given compounds.

Usage

```
rmSubs(object, mediac)

## S4 method for signature 'Arena'
rmSubs(object, mediac)
```

Arguments

`object`	An object of class Arena.
`mediac`	A character vector giving the names of substances, which should be added to the environment (the default takes all possible substances).

`selPheno` *Function for selecting phenotypes which occured on the arena from specific iterations and species*

Description

The generic function `selPheno` selects phenotypes from specific simulation step in an `Eval` object.

Usage

```
selPheno(object, time, type, reduce = F)

## S4 method for signature 'Eval'
selPheno(object, time, type, reduce = F)
```

Arguments

object	An object of class Eval.
time	A numeric vector giving the simulation steps which should be plotted.
type	A names indicating the species of interest in the arena.
reduce	A boolean variable indicating if the resulting matrix should be reduced.

Details

The phenotypes are defined by flux through exchange reactions, which indicate potential differential substrate usages.

Value

Returns a matrix with the substrate usage and the number of individuals using the phenotype.

See Also

Eval-class and getPhenoMat

Examples

```
data(Ec_core, envir = environment()) #get Escherichia coli core metabolic model
bac <- Bac(Ec_core,deathrate=0.05,
           minweight=0.05,growtype="exponential") #initialize a bacterium
arena <- Arena(n=20,m=20) #initialize the environment
arena <- addOrg(arena,bac,amount=10) #add 10 organisms
arena <- addSubs(arena,40) #add all possible substances
eval <- simEnv(arena,5)
selPheno(eval,time=5,type='ecoli_core_model',reduce=TRUE)
```

---

setKinetics *Function to set Michaelis-Menten kinetics for uptake of a substance*

Description

The generic function `setKinetics` provides kinetics for exchange reactions.

Usage

```
setKinetics(object, exchangeR, Km, vmax)

## S4 method for signature 'Organism'
setKinetics(object, exchangeR, Km, vmax)
```

Arguments

object	An object of class Organisms.
exchangeR	Name of an exchange reaction
Km	Parameter Michaelis-Menten-Kinetics (in mM)
vmax	Parameter Michaelis-Menten-Kinetics (in mmol/(g*h))

---

simBac *Function for one simulation iteration for objects of Bac class*

Description

The generic function simBac implements all neccessary functions for the individuals to update the complete environment.

Usage

```
simBac(object, arena, j, sublb, bacnum, sec_obj = "none", cutoff = 1e-06,
  pcut = 1e-06)

## S4 method for signature 'Bac'
simBac(object, arena, j, sublb, bacnum, sec_obj = "none",
  cutoff = 1e-06, pcut = 1e-06)
```

Arguments

object	An object of class Bac.
arena	An object of class Arena defining the environment.
j	The index of the organism of interest in orgdat.
sublb	A vector containing the substance concentrations in the current position of the individual of interest.
bacnum	integer indicating the number of bacteria individuals per gridcell
sec_obj	character giving the secondary objective for a bi-level LP if wanted.
cutoff	value used to define numeric accuracy.
pcut	A number giving the cutoff value by which value of objective function is considered greater than 0.

Details

Bacterial individuals undergo step by step the following procedures: First the individuals are constrained with `constrain` to the substrate environment, then flux balance analysis is computed with `optimizeLP`, after this the substrate concentrations are updated with `consume`, then the bacterial growth is implemented with `growth`, the potential new phenotypes are added with `checkPhen`, finally the additional and conditional functions `lysis`, `move` or `chemotaxis` are performed. Can be used as a wrapper for all important bacterial functions in a function similar to `simEnv`.

Value

Returns the updated enivironment of the `population` parameter with all new positions of individuals on the grid and all new substrate concentrations.

See Also

`Bac-class`, `Arena-class`, `simEnv`, `constrain`, `optimizeLP`, `consume`, `growth`, `checkPhen`, `lysis`, `move` and `chemotaxis`

Examples

```
NULL
```

`simBac_par` *Function for one simulation iteration for objects of Bac class*

Description

The generic function `simBac_par` implements all neccessary functions for the individuals to update the complete environment.

Usage

```
simBac_par(object, arena, j, sublb, bacnum, lpobject, sec_obj = "none",
  cutoff = 1e-06)

## S4 method for signature 'Bac'
simBac_par(object, arena, j, sublb, bacnum, lpobject,
  sec_obj = "none", cutoff = 1e-06)
```

Arguments

`object`	An object of class Bac.
`arena`	An object of class Arena defining the environment.
`j`	The index of the organism of interest in orgdat.
`sublb`	A vector containing the substance concentrations in the current position of the individual of interest.

bacnum	integer indicating the number of bacteria individuals per gridcell
lpobject	linar programming object (copy of organism@lpobj) that have to be a deep copy in parallel due to pointer use in sybil.
sec_obj	character giving the secondary objective for a bi-level LP if wanted.
cutoff	value used to define numeric accuracy

Value

Returns the updated enivironment of the `population` parameter with all new positions of individuals on the grid and all new substrate concentrations.

---

simEnv — *Main function for simulating all processes in the environment*

Description

The generic function `simEnv` for a simple simulation of the environment.

Usage

```
simEnv(object, time, lrw = NULL, continue = FALSE, reduce = FALSE,
  diffusion = TRUE, diff_par = FALSE, cl_size = 2, sec_obj = "none",
  cutoff = 1e-06, pcut = 1e-06)

## S4 method for signature 'Arena'
simEnv(object, time, lrw = NULL, continue = FALSE,
  reduce = FALSE, diffusion = TRUE, diff_par = FALSE, cl_size = 2,
  sec_obj = "none", cutoff = 1e-06, pcut = 1e-06)
```

Arguments

object	An object of class Arena or Eval.
time	A number giving the number of iterations to perform for the simulation
lrw	A numeric value needed by solver to estimate array size (by default lwr is estimated in the simEnv() by the function estimate_lrw())
continue	A boolean indicating whether the simulation should be continued or restarted.
reduce	A boolean indicating if the resulting `Eval` object should be reduced
diffusion	True if diffusion should be done (default on).
diff_par	True if diffusion should be run in parallel (default off).
cl_size	If diff_par is true then cl_size defines the number of cores to be used in parallelized diffusion.
sec_obj	character giving the secondary objective for a bi-level LP if wanted.
cutoff	value used to define numeric accuracy
pcut	A number giving the cutoff value by which value of objective function is considered greater than 0.

Details

The returned object itself can be used for a subsequent simulation, due to the inheritance between Eval and Arena.

Value

Returns an object of class Eval which can be used for subsequent analysis steps.

See Also

Arena-class and Eval-class

Examples

```
data(Ec_core, envir = environment()) #get Escherichia coli core metabolic model
bac <- Bac(Ec_core,deathrate=0.05,
           minweight=0.05,growtype="exponential") #initialize a bacterium
arena <- Arena(n=20,m=20) #initialize the environment
arena <- addOrg(arena,bac,amount=10) #add 10 organisms
arena <- addSubs(arena,40) #add all possible substances
eval <- simEnv(arena,5)
```

---

simEnv_par — *Main function for simulating in parallel all processes in the environment*

Description

The generic function simEnv_par for a simple in parallel all simulation of the environment.

Usage

```
simEnv_par(object, time, lrw = NULL, continue = FALSE, reduce = FALSE,
  cluster_size = NULL, diffusion = TRUE, sec_obj = "none",
  cutoff = 1e-06)

## S4 method for signature 'Arena'
simEnv_par(object, time, lrw = NULL, continue = FALSE,
  reduce = FALSE, cluster_size = NULL, diffusion = TRUE,
  sec_obj = "none", cutoff = 1e-06)
```

Arguments

object	An object of class Arena or Eval.
time	A number giving the number of iterations to perform for the simulation
lrw	A numeric value needed by solver to estimate array size (by default lwr is estimated in the simEnv() by the function estimate_lrw())

continue	A boolean indicating whether the simulation should be continued or restarted.
reduce	A boolean indicating if the resulting Eval object should be reduced
cluster_size	Number of cpu cores to be used.
diffusion	True if diffusion should be done (default on).
sec_obj	character giving the secondary objective for a bi-level LP if wanted.
cutoff	value used to define numeric accuracy

Details

The returned object itself can be used for a subsequent simulation, due to the inheritance between Eval and Arena.

Value

Returns an object of class Eval which can be used for subsequent analysis steps.

See Also

Arena-class and Eval-class

Examples

```
data(Ec_core, envir = environment()) #get Escherichia coli core metabolic model
bac <- Bac(Ec_core,deathrate=0.05,
           minweight=0.05,growtype="exponential") #initialize a bacterium
arena <- Arena(n=20,m=20) #initialize the environment
arena <- addOrg(arena,bac,amount=10) #add 10 organisms
arena <- addSubs(arena,40) #add all possible substances
eval <- simEnv(arena,5)
```

---

simHum   *Function for one simulation iteration for objects of Human class*

Description

The generic function simHum implements all neccessary functions for the individuals to update the complete environment.

Usage

```
simHum(object, arena, j, sublb, bacnum)

## S4 method for signature 'Human'
simHum(object, arena, j, sublb, bacnum)
```

Arguments

`object`	An object of class Human.
`arena`	An object of class Arena defining the environment.
`j`	The number of the iteration of interest.
`sublb`	A vector containing the substance concentrations in the current position of the individual of interest.
`bacnum`	integer indicating the number of bacteria individuals per gridcell

Details

Human cell individuals undergo the step by step the following procedures: First the individuals are constrained with `constrain` to the substrate environment, then flux balance analysis is computed with `optimizeLP`, after this the substrate concentrations are updated with `consume`, then the cell growth is implemented with `cellgrowth`, the potential new phenotypes are added with `checkPhen`, finally the conditional function `lysis` is performed. Can be used as a wrapper for all important cell functions in a function similar to `simEnv`.

Value

Returns the updated enivironment of the `arena` parameter with all new positions of individuals on the grid and all new substrate concentrations.

See Also

`Human-class`, `Arena-class`, `simEnv`, `constrain`, `optimizeLP`, `consume`, `cellgrowth`, `checkPhen` and `lysis`

Examples

```
NULL
```

---

statPheno *Function for investigating a specific phenotype of an organism*

Description

The generic function `statPheno` provides statistical and visual information about a certain phenotype.

Usage

```
statPheno(object, type_nr = 1, phenotype_nr, dict = NULL)

## S4 method for signature 'Eval'
statPheno(object, type_nr = 1, phenotype_nr, dict = NULL)
```

Arguments

`object`	An object of class Eval.
`type_nr`	A number indicating the Organism type of the phenotype to be investigated (from orgdat)
`phenotype_nr`	A number indicating the phenotype to be investigated (from orgdat)
`dict`	A character vector of all substance IDs with names that should be used instead of possibly cryptic IDs

Details

The phenotypes are defined by flux through exchange reactions, which indicate potential differential substrate usages.

See Also

`Eval-class`

Examples

```
data(Ec_core, envir = environment()) #get Escherichia coli core metabolic model
bac <- Bac(Ec_core,deathrate=0.05,
           minweight=0.05,growtype="exponential") #initialize a bacterium
arena <- Arena(n=20,m=20) #initialize the environment
arena <- addOrg(arena,bac,amount=10) #add 10 organisms
arena <- addSubs(arena,40) #add all possible substances
eval <- simEnv(arena,5)
statPheno(eval, type_nr=1, phenotype_nr=2)
```

`stirEnv` *Function for stirring/mixing the complete evironment*

Description

The generic function `stirEnv` simulates the event of mixing all substrates and organisms in the environment.

Usage

```
stirEnv(object, sublb)

## S4 method for signature 'Arena'
stirEnv(object, sublb)
```

Arguments

`object`	An object of class Arena.
`sublb`	A matrix with the substrate concentration for every individual in the environment based on their x and y position.

Details

The stirring is implemented as a random permutation of organism positions and the equalization of of all substrate concentrations.

Value

Returns the substrate concentration for every individual in the environment with substrates as well as x and y positions as columns and rows for each organism.

See Also

`Arena-class` and `getSublb`

Examples

```
data(Ec_core, envir = environment()) #get Escherichia coli core metabolic model
bac <- Bac(Ec_core,deathrate=0.05,
           minweight=0.05,growtype="exponential") #initialize a bacterium
arena <- Arena(n=20,m=20) #initialize the environment
arena <- addOrg(arena,bac,amount=10) #add 10 organisms
arena <- addSubs(arena,40) #add all possible substances
sublb <- getSublb(arena)
stirEnv(arena,sublb)
```

`Substance-class` *Structure of the S4 class "Substance"*

Description

Structure of the S4 class `Substance` representing substances in the environment which can be produced or consumed.

Slots

`smax` A number representing the start concentration of the substance for each grid cell in the environment.

`diffmat` A sparse matrix containing all concentrations of the substance in the environment.

`name` A character vector representing the name of the substance.

`id` A character vector representing the identifier of the substance.

`difunc` A character vector ("pde","cpp" or "r") describing the function for diffusion.

`difspeed` A number indicating the diffusion speed (given by cm^2/s).

`diffgeometry` Diffusion coefficient defined on all grid cells (initially set by constructor).

`pde` R-function that computes the values of the derivatives in the diffusion system

`boundS` A number defining the attached amount of substance at the boundary (Warning: boundary-function must be set in pde!)

`Substance-constructor` *Constructor of the S4 class* `Substance`

Description

The constructor to get a new object of class `Substance`

Usage

```
Substance(n, m, smax, gridgeometry, difspeed = 6.7e-06, ...)
```

Arguments

`n`	A number giving the horizontal size of the environment.
`m`	A number giving the vertical size of the environment.
`smax`	A number representing the start concentration of the substance for each grid cell in the environment.
`gridgeometry`	A list containing grid geometry parameter
`difspeed`	A number indicating the diffusion speed (given by cm^2/s).
`...`	Arguments of `Substance-class`

Value

Object of class `Substance`

`usd` *Computer standard deviation upper bound*

Description

Helper function to get upper error bounds in plotting

Usage

```
usd(y)
```

Arguments

`y`	Vector with numbers

Index

addDefaultMed, 4
addDefaultMed,Arena-method (addDefaultMed), 4
addEssentialMed, 4
addEssentialMed,Arena-method (addEssentialMed), 4
addEval, 5
addEval,Eval-method (addEval), 5
addOrg, 6, *14*
addOrg,Arena-method (addOrg), 6
addSubs, 7, *15*, *33*
addSubs,Arena-method (addSubs), 7
Arena (Arena-constructor), 9
Arena-class, 8, *9*
Arena-constructor, 9

Bac (Bac-Constructor), 10
Bac-class, 9, *10*
Bac-Constructor, 10
BacArena, 10
BacArena-package (BacArena), 10

cellgrowth, 11
cellgrowth,Human-method (cellgrowth), 11
changeDiff, 12
changeDiff,Arena-method (changeDiff), 12
changeFobj, 13
changeFobj,Human-method (changeFobj), 13
changeOrg, 14
changeOrg,Arena-method (changeOrg), 14
changeSub, *7*, *12*, *15*, 15, *22*
changeSub,Arena-method (changeSub), 15
checkCorr, 16
checkCorr,Eval-method (checkCorr), 16
checkPhen, 17, *37*
checkPhen,Arena-method (checkPhen), 17
checkPhen_par, 18
checkPhen_par,Arena-method (checkPhen_par), 18
chemotaxis, 18
chemotaxis,Bac-method (chemotaxis), 18
constrain, 19
constrain,Organism-method (constrain), 19
consume, 20
consume,Organism-method (consume), 20
createGradient, 21
createGradient,Arena-method (createGradient), 21

dat2mat, 22
dat2mat,Arena-method (dat2mat), 22
diffuse, 23
diffuse,Arena-method (diffuse), 23
diffuse_par, 25
diffuse_par,Arena-method (diffuse_par), 25
diffusePDE, 23, *25*
diffusePDE,Substance-method (diffusePDE), 23
diffuseR, *24*, 24
diffuseR,Substance-method (diffuseR), 24

emptyHood, *19*, 26, *46*
emptyHood,Organism-method (emptyHood), 26
Eval (Eval-constructor), 27
Eval-class, *27*, 27
Eval-constructor, 27
evalArena, 28
evalArena,Eval-method (evalArena), 28
extractMed, 29
extractMed,Eval-method (extractMed), 29

findFeeding, 30
findFeeding,Eval-method (findFeeding), 30
findFeeding2, 31
findFeeding2,Eval-method (findFeeding2), 31

findFeeding3, 32
findFeeding3,Eval-method (findFeeding3), 32
findInArena, 32
findInArena,Arena-method (findInArena), 32
flushSubs, 33
flushSubs,Arena-method (flushSubs), 33

getArena, 34
getArena,Eval-method (getArena), 34
getCorrM, *16*, 35
getCorrM,Eval-method (getCorrM), 35
getPhenoMat, 36, *45*, *62*
getPhenoMat,Eval-method (getPhenoMat), 36
getPhenotype, *17*, *36*, 37
getPhenotype,Organism-method (getPhenotype), 37
getSubHist, 38
getSubHist,Eval-method (getSubHist), 38
getSublb, *23*, 38, *70*
getSublb,Arena-method (getSublb), 38
getVarSubs, 39
getVarSubs,Eval-method (getVarSubs), 39
growExp, *11*, 40, *42*
growExp,Organism-method (growExp), 40
growLin, *11*, 40, *42*
growLin,Organism-method (growLin), 40
growth, 41
growth,Bac-method (growth), 41
growth_par, 42
growth_par,Bac-method (growth_par), 42

Human (Human-constructor), 43
Human-class, 42, *43*
Human-constructor, 43

lsd, 43
lysis, 44
lysis,Organism-method (lysis), 44

minePheno, *37*, 45
minePheno,Eval-method (minePheno), 45
move, 46
move,Organism-method (move), 46

NemptyHood, 47
NemptyHood,Organism-method (NemptyHood), 47

openArena, 48
optimizeLP, *13*, *40*, *41*, *44*, 48
optimizeLP,Organism-method (optimizeLP), 48
optimizeProb, *49*
Organism (Organism-constructor), 50
Organism-class, 49
Organism-constructor, 50

plotAbundance, 51
plotCurves, 51
plotCurves,Eval-method (plotCurves), 51
plotCurves2, 52
plotCurves2,Eval-method (plotCurves2), 52
plotFluxVar, 54
plotGrowthCurve, 54
plotInterNum, 55
plotPhenCurve, 55
plotPhenNum, 56
plotShadowCost, 56
plotShadowCost,Eval-method (plotShadowCost), 56
plotSpecActivity, 57
plotSubCurve, 57
plotSubUsage, 58
plotSubVar, 59
plotTotFlux, 59
plotTotFlux,Eval-method (plotTotFlux), 59

redEval, 60
redEval,Eval-method (redEval), 60
reset_screen, 61
rmSubs, 61
rmSubs,Arena-method (rmSubs), 61

selPheno, 61
selPheno,Eval-method (selPheno), 61
setKinetics, 62
setKinetics,Organism-method (setKinetics), 62
simBac, 63
simBac,Bac-method (simBac), 63
simBac_par, 64
simBac_par,Bac-method (simBac_par), 64
simEnv, *64*, 65, *68*
simEnv,Arena-method (simEnv), 65
simEnv_par, 66

simEnv_par,Arena-method *(*simEnv_par*)*, 66
simHum, 67
simHum,Human-method *(*simHum*)*, 67
statPheno, 68
statPheno,Eval-method *(*statPheno*)*, 68
stirEnv, 69
stirEnv,Arena-method *(*stirEnv*)*, 69
Substance *(*Substance-constructor*)*, 71
Substance-class, 70
Substance-constructor, 71
SYBIL_SETTINGS, *50*
sysBiolAlg, *49*

usd, 71
