# Supplementary material for: BacArena: Individual-based metabolic modeling of heterogeneous microbes in complex communities
Source: PLoS Comput Biol. 2017 May 22;13(5):e1005544. doi: 10.1371/journal.pcbi.1005544 (PMC5460873; doi:10.1371/journal.pcbi.1005544)
Supplement: S3 Text — Documentation of changes in metabolic model of P. aeruginosa and additional figures from replicates. (PDF) [file pcbi.1005544.s003.pdf]

Supplement text S3:

P. aeruginosa single-species biofilm model

1. Correction of metabolic model of P. aeruginosa to work with lactate

The genome scale metabolic model reconstructed by Oberhard2008 did not allow lactate production because exchange reactions were defined for S-lactate while intern reactions operated with R-lactate. We refined the model so that lactate production is possible as follows:

- new lactate dehydrogenase working with S-lactate (RR00405b)
- new glutathione hydrolase with S-lactate (IR00961b)
- permit lactate transport into cytoplasma (IR08776)

Oberhardt, Matthew A., Jacek Puchałka, Kimberly E. Fryer, Vítor AP Martins Dos Santos, and Jason A. Papin. "Genome-scale metabolic network analysis of the opportunistic pathogen Pseudomonas aeruginosa PAO1." Journal of bacteriology 190, no. 8 (2008): 2790-2803.

2. Production of acetate and succinate predicted by the metabolic model of *P. aeruginosa*

The production of different amounts of succinate and acetate is a function of available glucose and oxygen. The figure shows formation of acetate and succinate given fixed glucose levels and varying oxygen levels. First, glucose is oxidized using the available oxygen as electron acceptor and second, if oxygen is limiting, fermented with acetate and succinate as products. The production of succinate is highest for lower oxygen levels and decreases with rising availability of oxygen. Succinate is produced via fumarate respiration pathway which regenerates two NADH. When enough oxygen is available, NADH could be regenerated more efficiently via oxidative phsophorylation so that the formation of succinate decreases. The formation of acetate rises with decreasing succinate production until no succinate is formed and decreases for higher oxygen levels afterwards. The reason for the shift is the application of the complete TCA which could be used for higher oxygen

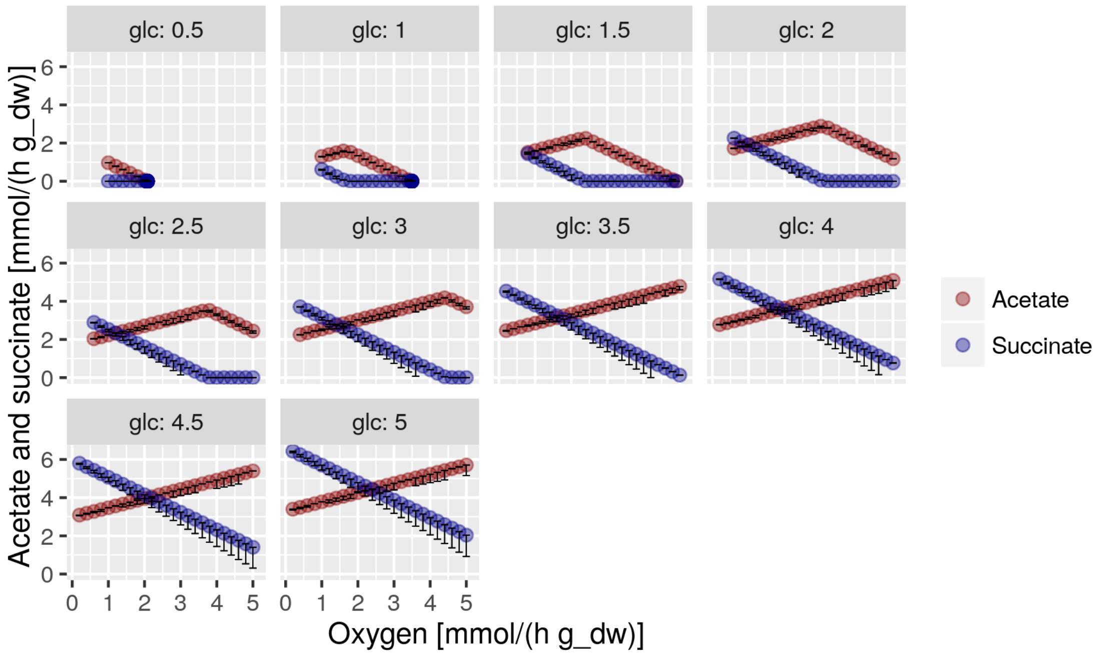

Figure 1: Production of acetate and succinate for varying levels of glucose (glc) and oxygen.

levels and after fumarate reductase activity (fumarate respiration) stopped and could be replaced by the succinate dehydrogenase of the TCA. Computation of FBA was done with the sybil package in R.

3. Influence of acetate and succinate on growth (metabolic model of *P. aeruginosa*)

During the *P. aeruginosa* simulation, we could identify several phenotypes. Acetate and/or succinate were produced by P4, P6 and P7 and were consumed again by P5, P7 and P8. In order to validate the phenotype preference, we compared growth rates of *P. aeruginosa* for different glucose and oxygen levels with and without the addition of acetate and succinate. The addition of acetate only was identical to growth of the control (no acetate and no succinate) except in cases where no glucose was available (acetate was used as carbon source) and for higher oxygen levels where all glucose oxidized with oxygen as terminal electron acceptor before. The addition of succinate showed to have a bigger influence on growth. Succinate alone as well as succinate and acetate together increased the growth rate to the same extent. Unlike acetate, succinate could increase growth rate even for lower oxygen levels. Computation of FBA was done with the sybil package in R.

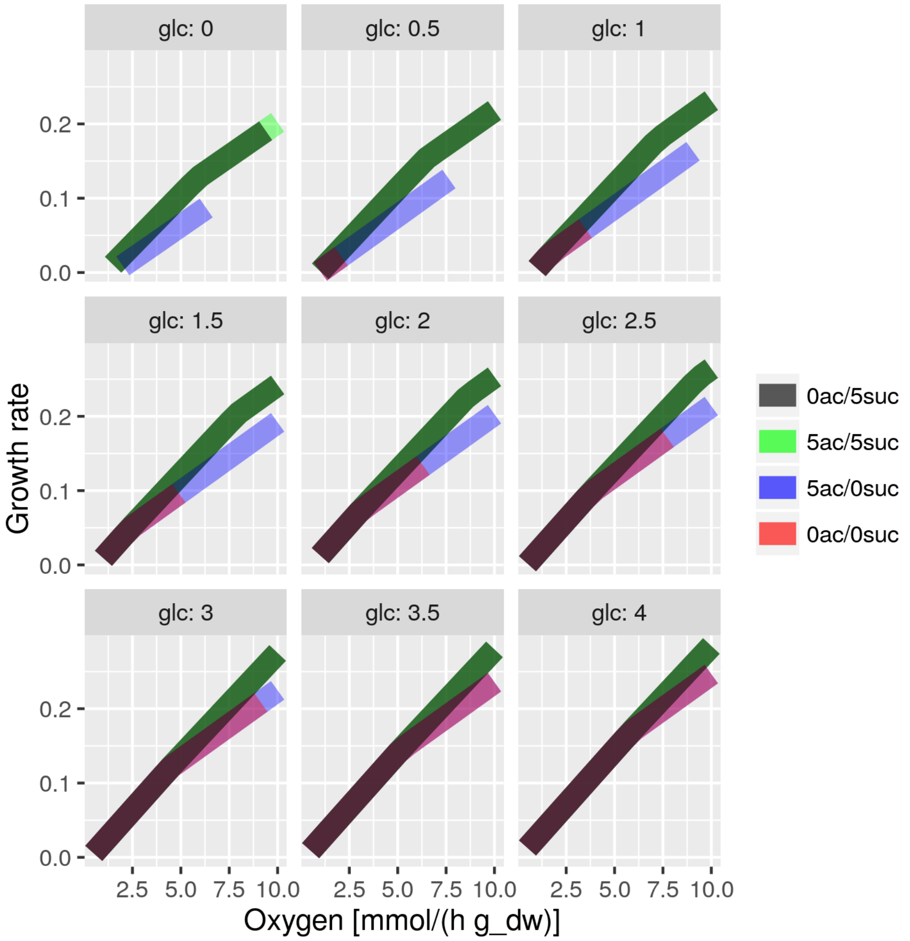

Figure 2: Influence of addition of acetate and succinate on growth rate.

4. Sensitivity analysis of oxygen and glucose initial conditions on phenotypes of *P. aeruginosa*

In case of simulation of *P. aeruginosa*, we discussed the occurrence of several phenotypes during biofilm formation (Figure 1, main text). The phenotypes dynamics could be shown to be stable for

all replicates (Figure S3). Furthermore in former section three and four, we could explain the occurrence of fermenting and oxidizing phenotypes by the underlying metabolic model. Additionally, we repeated the biofilm simulation for 100 times with varying glucose and oxygen levels to estimate the stability of the phenotypes with respect to initial values of oxygen and glucose. In Figure 3 it is shown that the number of phenotypes for oxygen and glucose levels which were close to the ones used during the original simulation are similar or equal. Less phenotypes occurred when oxygen/glucose ratio were higher because succinate related phenotypes disappear for higher oxygen levels (cf. Section 2). Identically, less phenotypes occurred in case of low oxygen levels because only fermenting phenotypes were feasable.

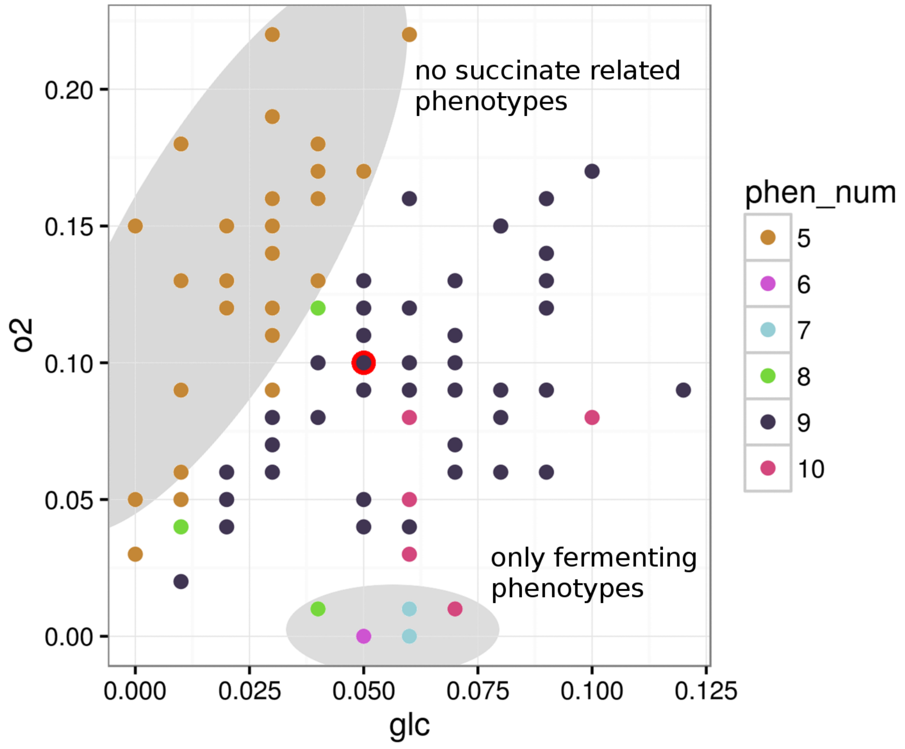

Figure 3: Number of phenotypes for different oxygen and glucose levels. The initial valued used for the main simulation is colored in red.

<u>5. Carbon dioxid fixation of *P. aeruginosa*</u>

So far, no CO_2 fixation has been described for *P. aeruginosa* and an inhibitory effect has been reported for higher *CO_2* concentrations. In contrast, a positive effect on growth has been reported in case of related *Pseudomonas* species so that a contribution of *CO_2* to the growth of *P. aeruginosa* might be possible, too. The observed *CO_2*-fixation is done by no known fixation pathway. CO_2 was converted to bicarbonate and utilized via the pyruvate carboxylase which consumed pyruvate and bicarbonate to form oxaloacetate. The energetics seems to be less favorable because ATP is needed and also pyruvate. This indicates no more than a supportive role of the pathway. The pyruvate carboxylase is an anaplerotic reaction but was shown to be able to fixate CO2 in vitro and is discussed to be part in synthetic CO2 fixation pathways.

King, A. D. & Nagel, C. W.:Influence of carbon dioxide upon the metabolism of Pseudomonas aeruginosa
Journal of Food Science, Wiley Online Library, 1975, *40*, 362-366

Gill, C. & Tan, K.: Effect of carbon dioxide on growth of Pseudomonas fluorescens.
Applied and Environmental Microbiology, Am Soc Microbiol, 1979, *38*, 237-240

Scrutton, M. C. & Utter, M. F.: Pyruvate carboxylase V. Interaction of the enzyme with adenosine triphosphate
Journal of Biological Chemistry, ASBMB, 1965, *240*, 3714-3723

Bar-Even, A.; Noor, E.; Lewis, N. E. & Milo, R.: Design and analysis of synthetic carbon fixation pathways
PNAS, 2010, *107*, 8889-8894
