# Supplementary figures and images for: BacArena: Individual-based metabolic modeling of heterogeneous microbes in complex communities
Source: PLoS Comput Biol. 2017 May 22;13(5):e1005544. doi: 10.1371/journal.pcbi.1005544 (PMC5460873; doi:10.1371/journal.pcbi.1005544)

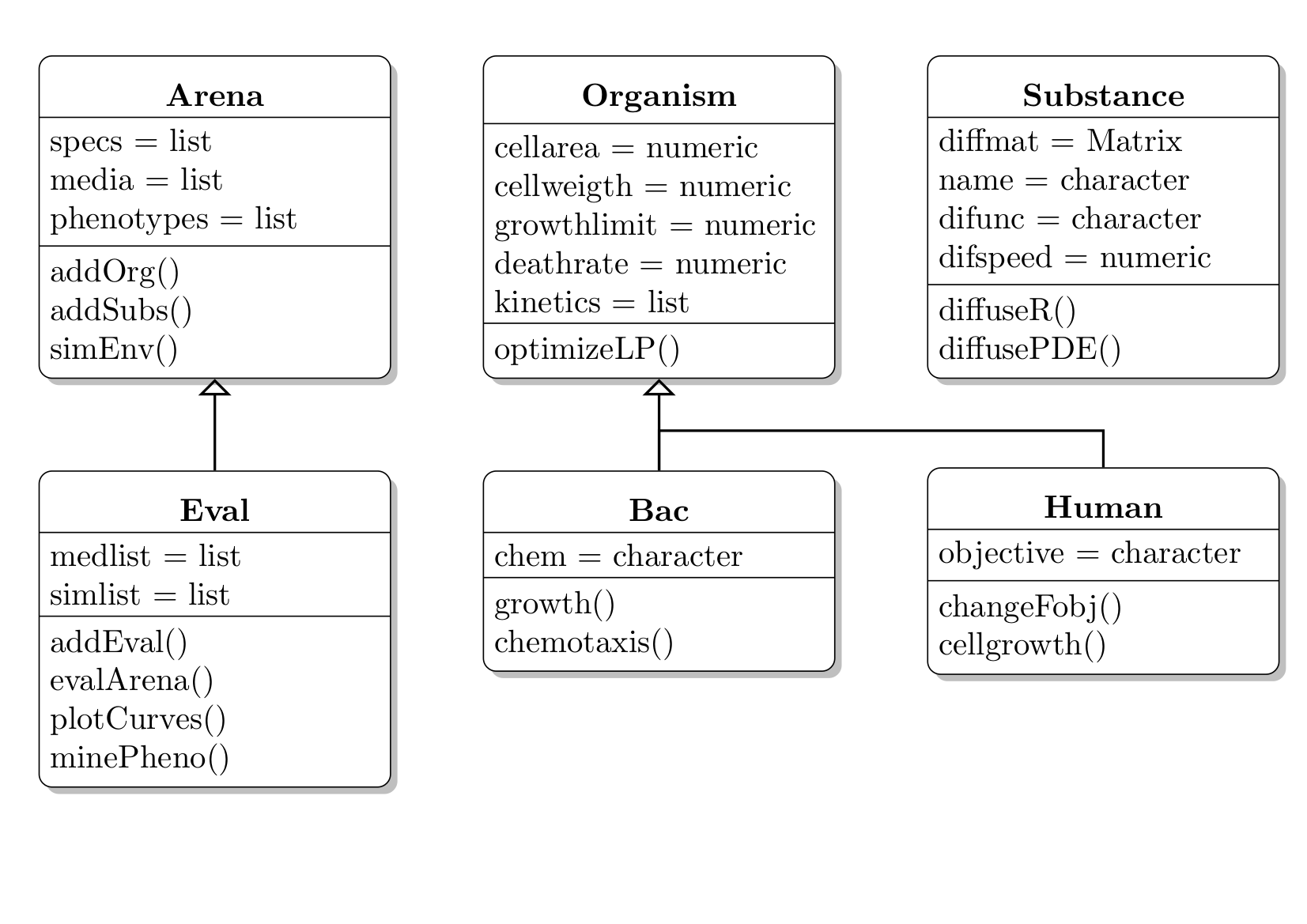

Supplement: S1 Fig — Simplified class diagram displaying the inheritance hierarchy. (TIF) [file pcbi.1005544.s010.tif]

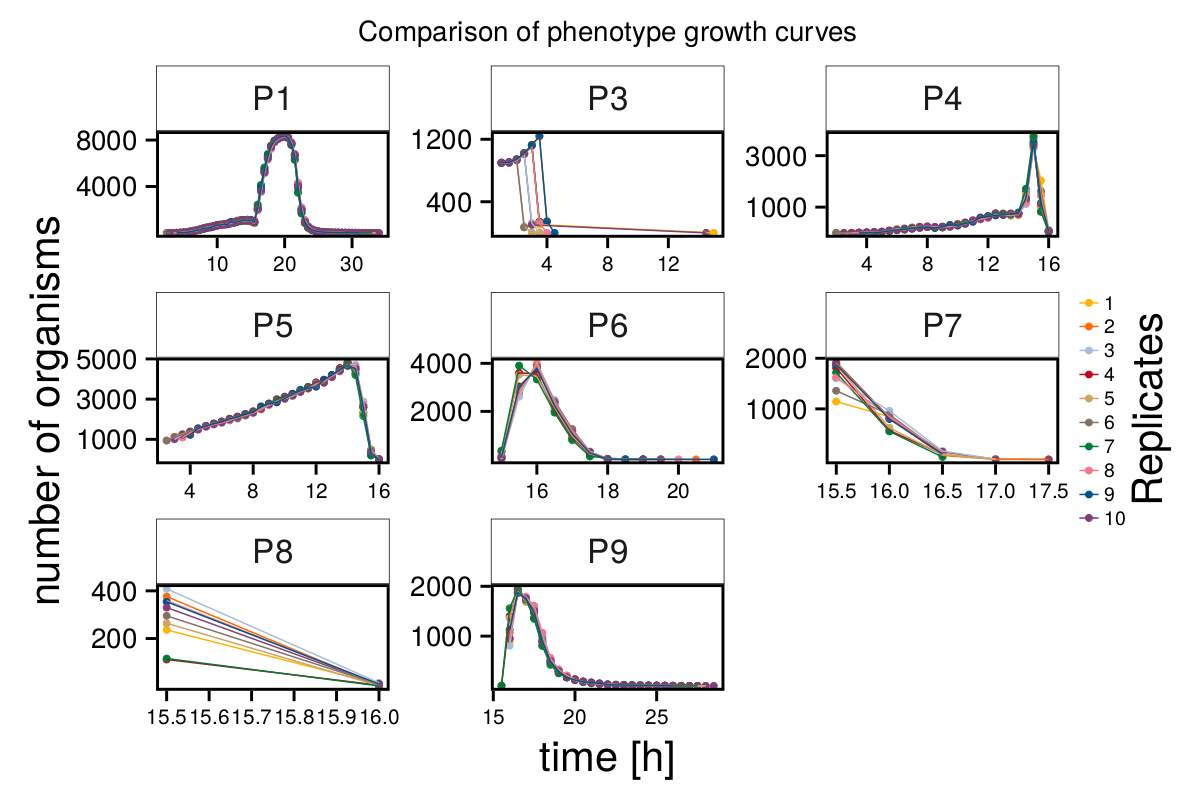

Supplement: S2 Fig — For each phenotype (P2,P3,…,P9) of the P. aeruginosa biofilm simulation the time curves for all replicates are shown. While the overall dynamics were stable, the occurrences of P3, P7 and P8 showed some minor variance. (TIF) [file pcbi.1005544.s011.tif]

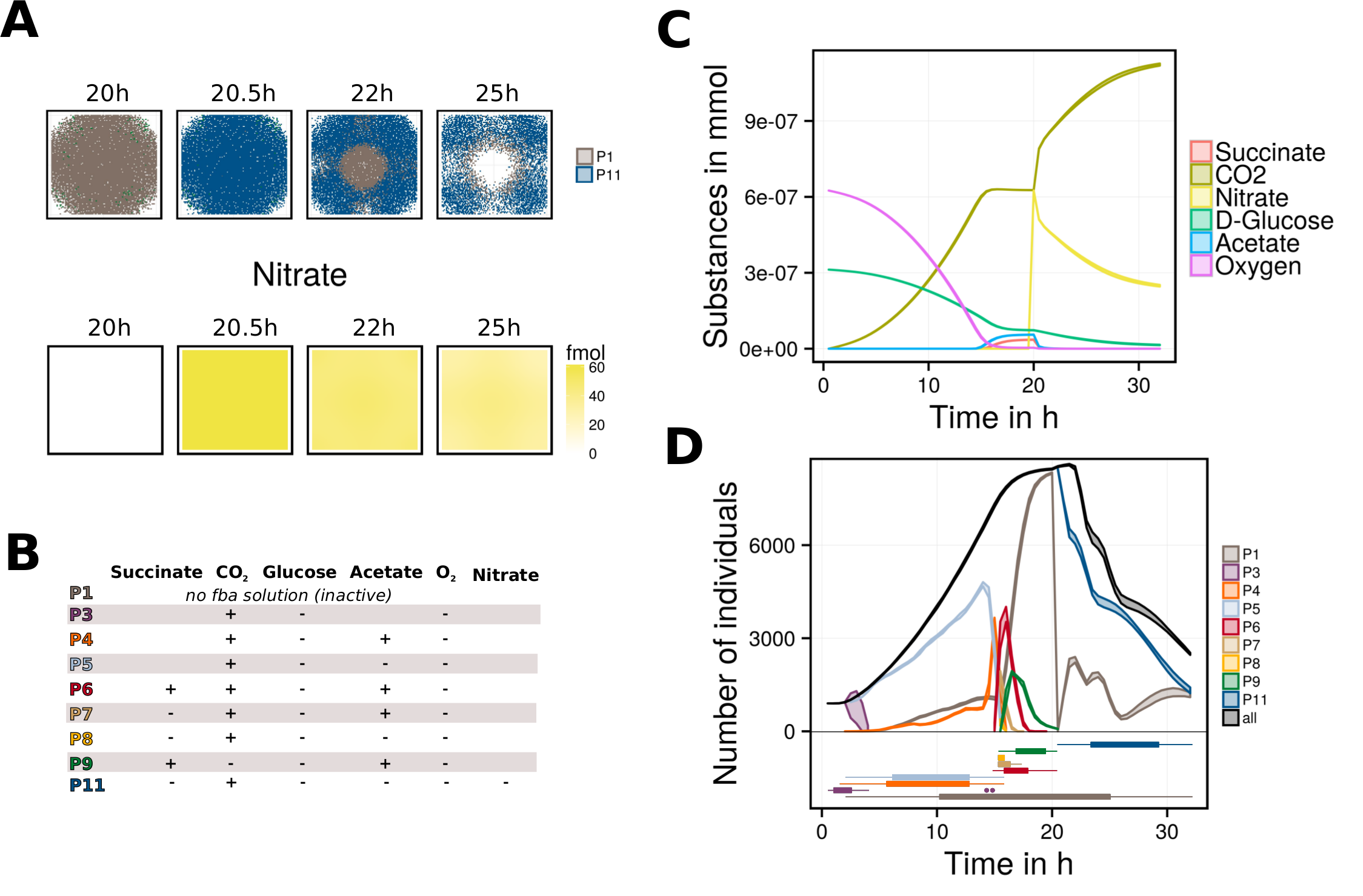

Supplement: S3 Fig — Alternative scenario of P. aeruginosa biofilm simulation with 0.1 mM nitrate added after 20 hours simulation time. A Spatial distribution of phenotypes and nitrate. The presence of nitrate after 20 hours was accomplished by a new nitrate consuming phenotype P11. B Comparison of phenotypes. C Time curve of core metabolites. The addition of nitrate after 20 hours lead to further glucose usage and CO2 production. The former produced acetate and succinate were used again. D Phenotypes growth curve. After the addition of nitrate, the metabolic inactive phenotype P1 vanished and the new nitrate consuming phenotype P11 emerged. (TIF) [file pcbi.1005544.s012.tif]

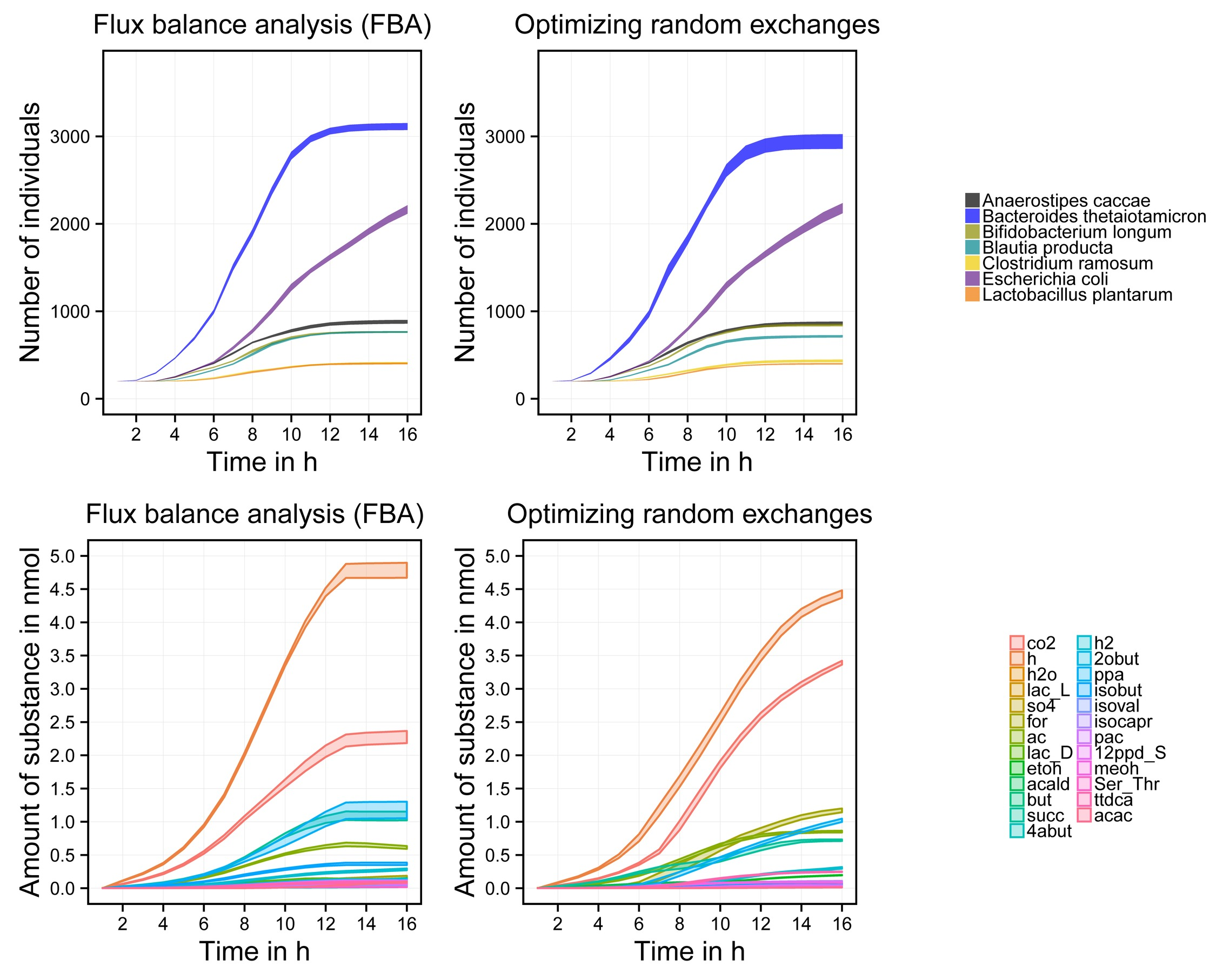

Supplement: S4 Fig — The first row represents the species growth and the second row the concentration change of the 25 most variable metabolites. The first columns shows a default flux balance analysis and the second column the optimization of a random exchange reaction as a secondary objective. The curve range shows a standard deviation of 10 replicate simulations each simulating 16 hours. (TIF) [file pcbi.1005544.s013.tif]
